# Sph-mediated mtDNA release and autophagy-dependent ferroptosis contribute to *Staphylococcus aureus*-induced mastitis

**DOI:** 10.1371/journal.ppat.1014644

**Published:** 2026-09-29

**Authors:** Yunfei Wei, Xinyu Zhao, Ziyi Yin, Yanmin Zhang, Yaxin Zhai, Baihe Zhan, Yeling Wang, Jiakang He, Jianfeng Wang, Xuming Deng, Lei Xu, Haihua Feng

**Affiliations:** 1 State Key Laboratory for Diagnosis and Treatment of Severe Zoonotic Infectious Diseases, Key Laboratory for Zoonosis Research of the Ministry of Education, Institute of Zoonosis, and College of Veterinary Medicine, Jilin University, Changchun, China; 2 Departments of Cardiovascular Medicine, First Hospital, Jilin University, Changchun, China; 3 College of Animal Science and Technology, Guangxi University, Nanning, Guangxi, China; University of Washington, UNITED STATES OF AMERICA

## Abstract

*Staphylococcus aureus* (*S. aureus*), a major pathogen of mastitis, employs a diverse arsenal of virulence factors to invade and disrupt mammary tissues, thereby eliciting a complex and dysregulated inflammatory response. Sphingomyelin phosphodiesterase (Sph) has emerged as a key virulence determinant of *S. aureus*; however, its potential role and underlying mechanisms in mastitis remain elusive. In this study, we demonstrate that Sph deficiency significantly suppresses *S. aureus*-induced ferroptosis. Mechanistically, Sph disrupts mitochondrial cristae through interaction with Mic60, a core component of the mitochondrial contact site and cristae-organizing system (MICOS). Sph-mediated cytosolic mitochondrial DNA (mtDNA) released is sensed by cyclic GMP-AMP synthase (cGAS), leading to activation of the STING signaling pathway, which further triggers the recruitment of GPX4 into the autophagy-lysosome pathway for degradation. Collectively, our study unveils a novel pathogenic mechanism whereby the *S. aureus* virulence factor Sph perturbs mitochondrial homeostasis, and engages the cGAS-STING axis to promote GPX4 degradation and ferroptosis, highlighting Sph as a potential therapeutic target for *S. aureus*-associated mastitis.

## Author summary

*S. aureus* is a major etiological agent of mastitis, a prevalent inflammatory disease of the mammary gland that adversely affects animal health and milk production. During infection, *S. aureus* deploys a diverse repertoire of virulence factors to damage host cells and promote disease progression. Among these factors, sphingomyelin phosphodiesterase (Sph) has been implicated in host cell injury; however, its contribution to mastitis pathogenesis remains poorly understood. Here, we show that Sph perturbs mitochondrial homeostasis by interacting with Mic60 and disrupting mitochondrial cristae architecture, thereby triggering activation of the cGAS-STING signaling pathway. Activated STING subsequently promotes the autophagic degradation of GPX4, a key antioxidant enzyme that protects cells against lipid peroxidation, ultimately driving ferroptotic cell death. Collectively, our findings uncover a previously unrecognized mechanism, and identify Sph as a potential therapeutic target for *S. aureus*-associated mastitis.

## Introduction

Bovine mastitis, a major cause of economic loss in the global dairy industry, is primarily caused by *Staphylococcus aureus* (*S. aureus*), a ubiquitous Gram-positive pathogen with significant implications for public health [1]. Alarmingly, the clinical spectrum of *S. aureus* infection in humans ranges from localized abscesses to severe systemic diseases such as pneumonia, bacteremia, endocarditis, and osteomyelitis [2]. In the livestock and poultry industry, *S. aureus* is a major cause of mastitis, which can progress to severe suppurative inflammation and substantially compromise the structural and functional integrity of the mammary gland, thereby causing considerable economic losses to the dairy industry [3,4]. Therefore, elucidating the underlying mechanisms involved in *S. aureus*-induced host infections and developing novel therapeutic strategies to reduce bacterial pathogenicity or bolster host defense are pivotal for addressing *S. aureus* infections.

The genome of *S. aureus* encodes various virulence factors, including toxins, enzymes, adhesins, and other surface-associated proteins, which coordinate the pathogenic process by facilitating bacterial adhesion, invasion, dissemination, and immune evasion [5]. In contrast to receptor-dependent pore-forming toxins such as α-hemolysin, it was reported that β-hemolysin, also known as sphingomyelin phosphodiesterase (Sph), exerts its hemolytic activity primarily through the enzymatic hydrolysis of sphingomyelin in host cell membranes. Notably, Sph exhibits a characteristic “hot-cold” hemolytic phenotype, with moderate hemolysis at 37°C followed by markedly enhanced lysis after subsequent incubation at 4°C [6,7]. Moreover, Sph functions as a multifunctional effector that subverts host defenses through leukotoxicity and modulation of innate immune responses, while also inducing diverse forms of cell necrosis or apoptosis. The recent identiﬁcation demonstrated that Sph provides a common structural framework belonging to the DNase I superfamily, consistent with its well-documented DNA-binding activity [8,9]. Furthermore, functional domain mapping of Sph identified His150 and His289 as critical residues for sphingomyelinase activity [8], whereas Thr149 and His162 contribute to DNA binding [10], indicating functionally distinct active sites. The sphingomyelinase and DNA-binding activities of Sph are crucial for promoting the formation of vegetations in infectious endocarditis, underscoring its potential role in disease progression. Recent evidence has emerged indicating that Sph knockout or antibody neutralization mitigates *S. aureus*-induced pneumonia in mice, implicating Sph as a key determinant of *S. aureus* pathogenicity [11]. Therefore, elucidating the molecular mechanisms by which Sph modulates host responses and contributes to *S. aureus* pathogenesis may provide new insights into the development of targeted therapeutic strategies.

The pathogenesis and progression of numerous diseases, including mastitis of infectious origin, involve multiple forms of regulated cell death (RCD), such as apoptosis, necroptosis, and ferroptosis [12]. Of particular note, ferroptosis is an iron-dependent form of regulated cell death characterized by excessive accumulation of phospholipid peroxides, ultimately resulting in lethal membrane damage. Glutathione peroxidase 4 (GPX4) acts as the key regulator by the elimination of phospholipid hydroperoxides, thereby preventing ferroptosis [13]. Ferroptosis is closely linked to mitochondrial iron metabolism, and is accompanied by distinct morphological alterations of mitochondria during ferroptosis, including disintegration of mitochondrial cristae and rupture of the outer mitochondrial membrane [14]. Importantly, the integrity of mitochondrial cristae is critical for maintaining mitochondrial homeostasis and limiting the release of mitochondrial DNA (mtDNA), thereby restraining aberrant inflammatory responses [15]. Growing evidence indicates that the bacterial virulence factor can manipulate host mitochondrial dynamics to promote infection. For example, listeriolysin O (LLO) produced by *Listeria monocytogenes* induces mitochondrial fission, highlighting a strategy by which intracellular bacteria subvert mitochondrial function to enhance pathogenicity [16]. Furthermore, bacterial toxin was found to trigger mitochondrial dysfunction and perturb redox homeostasis, thereby facilitating the release of damaged mtDNA into the cytosol and activating the cyclic GMP-AMP synthase-stimulator of interferon genes (cGAS-STING) pathway and downstream type I interferon responses [17]. In addition to canonical inflammatory signaling, STING can initiate autophagy independently of IRF3/NF-κB activation, and STING trafficking-induced autophagy has been proposed to represent a primordial function of the cGAS-STING pathway [18]. Recent studies have further demonstrated that drug-induced mtDNA release can activate STING-dependent, autophagy-associated ferroptosis, which occurs independently of type I interferon responses [19]. Mechanistically, STING signaling has been implicated in promoting ferroptosis by facilitating the autophagic degradation of GPX4, thereby disrupting cellular redox homeostasis. This regulatory mechanism, observed in diverse pathological contexts ranging from neuroinflammation to cardiomyocyte injury, supports the existence of a functional STING-GPX4 axis linking innate immune signaling to ferroptotic cell death [20]. The functional importance of this STING-GPX4 regulatory axis is further supported by mutagenesis studies showing that disruption of the STING-GPX4 interaction through mutations at key residues, including T267 in STING and N146 in GPX4, attenuates GPX4 degradation and ferroptotic cell death [21]. Collectively, these findings prompted us to investigate whether and how Sph contributes to *S. aureus*-induced ferroptosis and to define the underlying molecular mechanisms.

Although *S. aureus* has been reported to induce diverse forms of regulated cell death, the molecular mechanisms by which it triggers mitochondria-associated ferroptosis remain incompletely understood. Herein, we report that *S. aureus* virulence factor Sph acts as a pivotal mitochondrial disruptor by directly interacting with Mic60, a core component of the mitochondrial contact site and cristae organizing system (MICOS), leading to cristae disorganization and subsequent mitochondrial DNA (mtDNA) release. The liberated mtDNA subsequently activates STING signaling, which promotes the autophagic degradation of GPX4, a key suppressor of ferroptosis, thereby driving ferroptotic cell death. Collectively, our study elucidates a novel pathogenic mechanism through which Sph governs autophagy-dependent ferroptosis, thereby laying the theoretical groundwork for future inhibitor development and therapeutic intervention.

## Results

### *S. aureus*-induced mammary epithelial cell death is associated with ferroptosis

To investigate the correlation between *S. aureus*-induced mastitis and ferroptosis, we established an *in vitro S. aureus*-infection model using bovine mammary epithelial MAC-T cells infected with *S. aureus* strain ATCC25923. Infection at a multiplicity of infection (MOI) of 200 for 6 h resulted in approximately 40% cell death (Fig 1A), whereas no significant cell death was observed during the initial 3 h of infection under the same conditions (Fig 1B). Consistently, *S. aureus* triggered MAC-T cell death in an MOI-dependent manner. As a positive control, treatment with the ferroptosis inducer erastin similarly induced substantial cell death in MAC-T cells (Fig 1C, D). Furthermore, to assess the effect of ferroptosis in *S. aureus*-induced MAC-T cell death, we employed five different types of ferroptosis inhibitors, including the lipid peroxidation inhibitor ferrostatin-1, the lipoxygenase inhibitor zileuton, the nitroxide radical TEMPO (a selective mitochondrial ROS scavenger), the ACSL4 inhibitor troglitazone, and the iron chelator deferiprone (Fig 1E). As expected, these inhibitors effectively mitigated *S. aureus*-induced MAC-T cell death, with TEMPO and deferiprone providing the most pronounced protection (Fig 1F-J). Moreover, TEMPO and deferiprone reduced *S. aureus*-induced cell death in a concentration-dependent manner at concentrations of up to 30 μM (Fig 1I, J), further supporting the involvement of oxidative stress and iron-dependent ferroptotic processes in *S. aureus*-induced cytotoxicity. In contrast, troglitazone showed diminished protective effects at concentrations ≥20 μM and tended to increase cell death, potentially owing to cytotoxicity at higher concentrations (Fig 1H).

**Fig 1:**
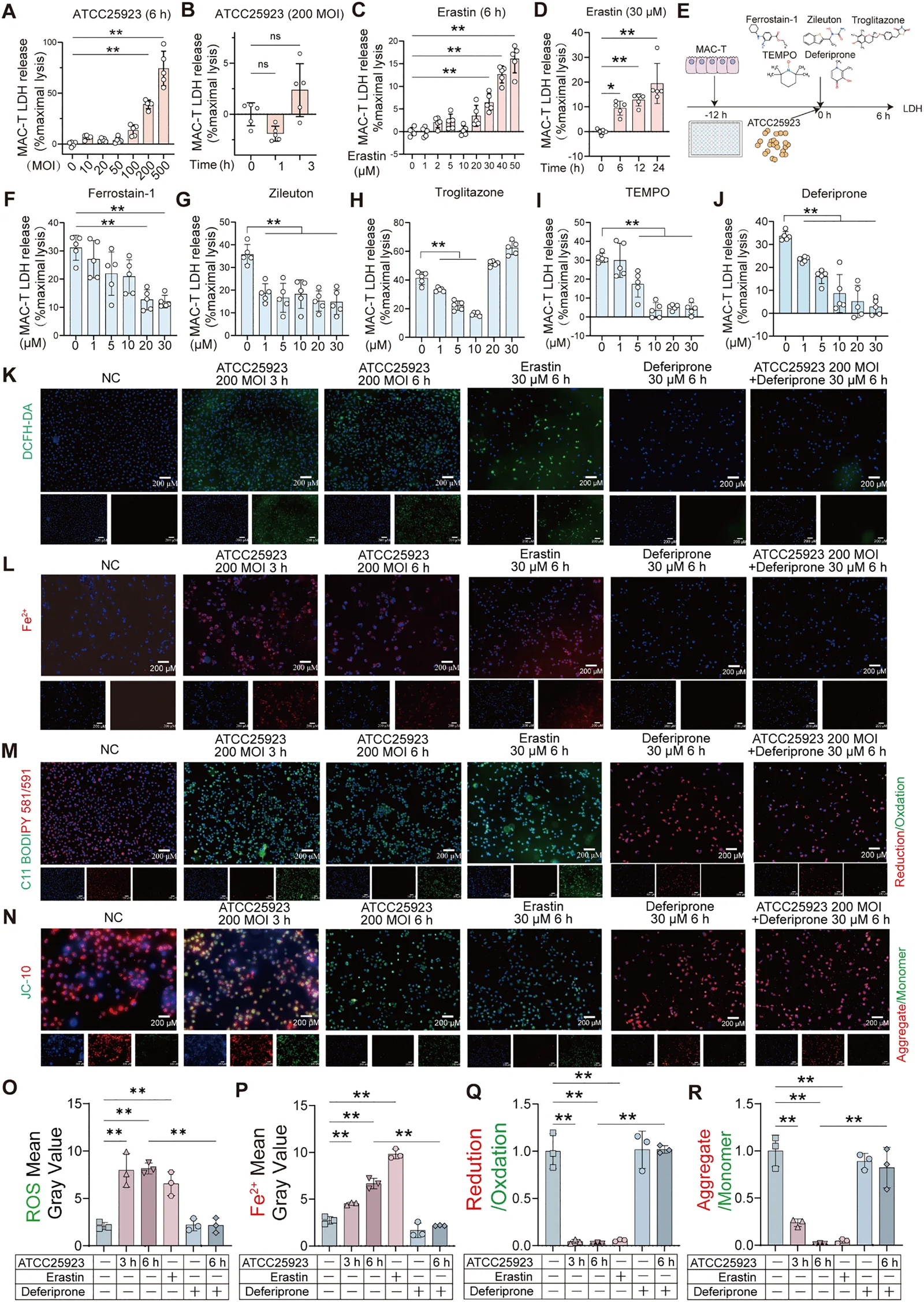
Ferroptosis inhibitors alleviate *S. aureus*-induced cytotoxicity and oxidative damage in MAC-T cells. **(A)** LDH release from MAC-T cells infected with *S. aureus* ATCC25923 strain at different MOIs for 6 h (n = 5). **(B)** LDH release from MAC-T cells infected with ATCC25923 (MOI = 200) for the indicated times (n = 5). **(C)** LDH release from MAC-T cells treated with the indicated concentrations of erastin for 6 h (n = 5). **(D)** LDH release from MAC-T cells treated with 30 μM Erastin for 0, 6, 12, and 24 h (n = 5). **(E)** Schematic diagram of the experimental protocol for ferroptosis inhibitor intervention in *S. aureus*-infected MAC-T cells. **(F-J)** LDH release from MAC-T cells infected with *S. aureus* ATCC25923 strain (MOI = 200) in the presence of the indicated concentrations (0-30 μM) of ferrostatin-1 **(F)**, zileuton **(G)**, troglitazone **(H)**, TEMPO **(I)**, or deferiprone **(J)** for 6 h (n = 5). **(K-N)** Representative fluorescence images and **(O-R)** corresponding quantitative analyses of intracellular ROS (DCFH-DA; **K**, **O)**, Fe^2+^ accumulation **(L, P)**, lipid peroxidation (C11 BODIPY 581/591; **M**, **Q)**, and mitochondrial membrane potential (JC-10; **N, R**) in MAC-T cells subjected to *S. aureus* infection (MOI = 200), erastin treatment, or deferiprone intervention for the indicated durations (n = 3). Scale bars, 200 μm. Data are presented as mean ± SD. *P < 0.05; **P < 0.01.

Moreover, to investigate whether *S. aureus* infection induces ferroptosis, we monitored the key hallmarks of ferroptosis in MAC-T cells using specific fluorescent probes. As shown in Fig 1K and O, both *S. aureus* infection and erastin treatment markedly increased the green fluorescence intensity of the redox-sensitive probe DCFH-DA compared with the negative control (NC), indicating enhanced intracellular ROS accumulation, which was subsequently reversed by deferiprone treatment. Likewise, staining with an Fe^2+^ probe revealed a pronounced elevation in intracellular fluorescence in *S. aureus*-infected and erastin-treated cells, indicating expansion of the labile Fe^2+^ pool, a critical catalyst for ferroptosis (Fig 1L, P). Moreover, the C11 BODIPY 581/591 probe, which exhibits a fluorescence emission shift from red to green upon oxidation, showed a marked increase in green fluorescence in *S. aureus*-infected and erastin-treated cells, which directly visualizes extensive lipid peroxidation within the cellular membranes (Fig 1M, Q). Consistently, a robust loss of mitochondrial membrane potential following either *S. aureus* infection or erastin treatment (Fig 1N, R). Critically, deferiprone effectively attenuated ferroptosis, as demonstrated by the reduced intracellular ROS and labile Fe^2+^ levels, suppressed lipid peroxidation, and restored mitochondrial membrane potential (Fig 1K-R). Together, these findings demonstrate that *S. aureus* infection triggers ferroptosis in mammary epithelial cells and that iron chelation with deferiprone protects against *S. aureus*-induced cellular injury.

### Deletion of *sph* significantly attenuates *S. aureus*-induced ferroptosis in mammary epithelial cells

To elucidate the function of the sphingomyelin phosphodiesterase (Sph), we generated an *sph* deletion mutant using a CRISPR/Cas9-mediated gene-editing strategy. In brief, an *sph*-specific sgRNA directed Cas9 to introduce a double-strand break at the chromosomal *sph* locus, followed by homologous recombination with a donor DNA fragment designed to delete the target gene, thereby generating an isogenic Δ*sph* mutant (S1A Fig). To confirm that the resulting phenotypes were specifically attributable to *sph* deletion, we further constructed a complemented strain by introducing a pLI50-derived plasmid carrying an intact copy of *sph* into the Δ*sph* mutant, thereby generating the Δ*sph*::*sph* complemented strain (S1B Fig).

We further assessed the effects of *sph* deletion on fundamental bacterial physiology and virulence-associated phenotypes. Growth curve analysis revealed that deletion of sph had no detectable effect on the growth kinetics of *S. aureus* ATCC25923 (Fig 2A), indicating that this gene is dispensable for basic bacterial proliferation. In contrast, the Δ*sph* mutant exhibited a markedly reduced capacity for biofilm formation (Fig 2B), consistent with previous reports implicating *Sph* in *S. aureus* biofilm development [9,10]. We next examined the contribution of Sph to *S. aureus*-induced host cell injury using an LDH release assay. Infection with the wild-type (WT) strain induced substantial cytotoxicity in MAC-T cells, whereas deletion of *sph* significantly reduced LDH release; this phenotype was restored in the complemented Δ*sph*::*sph* strain (Fig 2C). Consistently, larvae infected with the Δ*sph* mutant exhibited significantly improved survival compared with those infected with the WT or Δ*sph*::*sph* strains in a *Galleria mellonella* infection model (Fig 2D). Notably, infection with either WT *S. aureus* or the complemented Δ*sph*::*sph* strain markedly reduced GPX4 protein levels in MAC-T cells, whereas GPX4 abundance was largely preserved following infection with the Δ*sph* mutant (Fig 2E, F). Consistent with these findings, deletion of *sph* markedly attenuated multiple ferroptosis-associated alterations induced by *S. aureus*, including intracellular ROS accumulation (Fig 2G, K), a diminished labile Fe^2+^  pool (Fig 2H, L), attenuated lipid peroxidation (Fig 2I, M), and loss of mitochondrial membrane potential (Fig 2J, N). Collectively, these findings demonstrate that Sph contributes to *S. aureus* virulence and promotes ferroptosis-associated injury in mammary epithelial cells.

**Fig 2:**
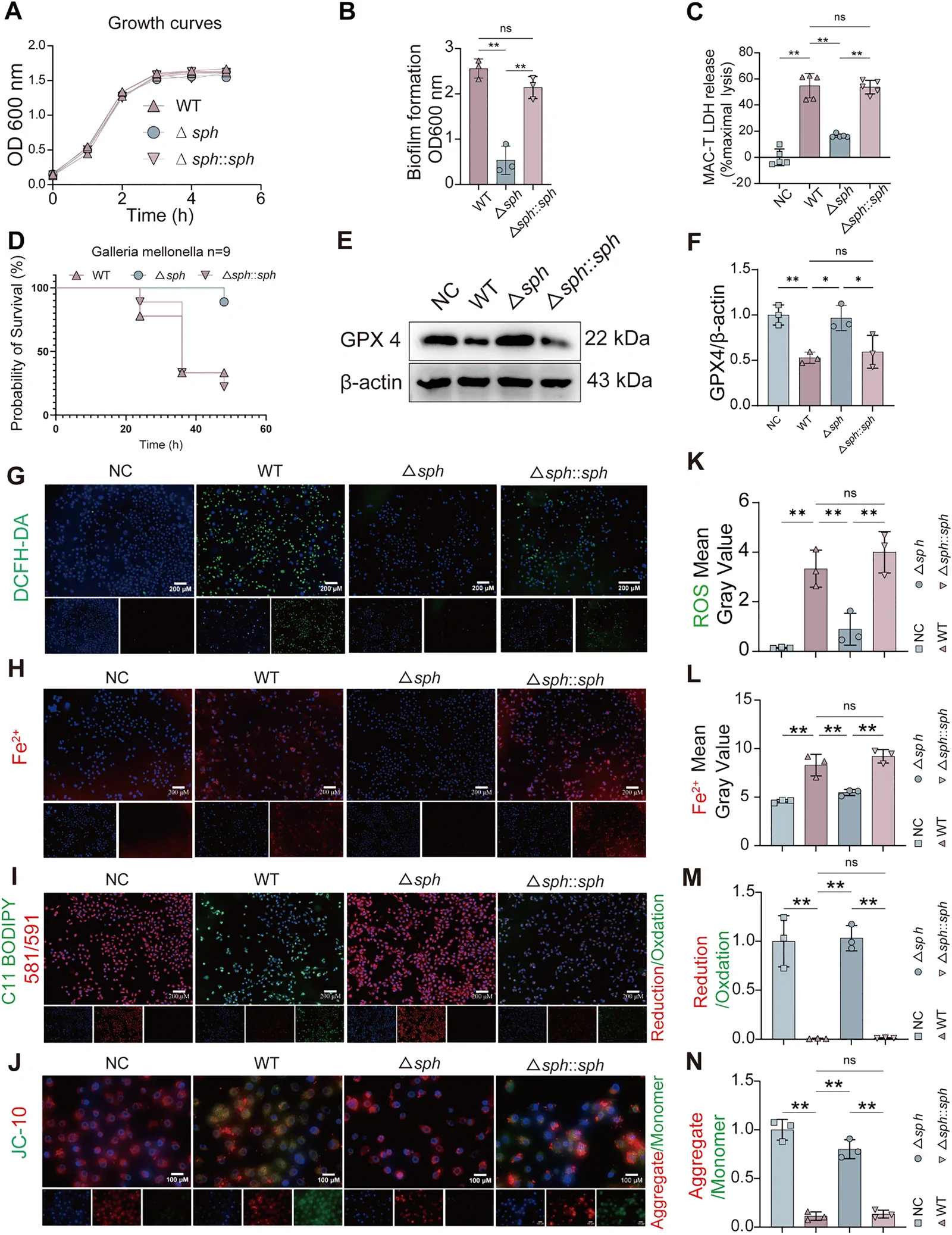
The *sph* deletion mutant attenuates ferroptosis-associated phenotypes and bacterial virulence. **(A)** Growth curves of *S. aureus* WT, Δ*sph*, and Δ*sph*::*sph* strains (n = 3). **(B)** Biofilm formation of *S. aureus* WT, Δ*sph*, and Δ*sph*::*sph* strains (n = 3). **(C)** LDH release from MAC-T cells infected with *S. aureus* WT, Δ*sph*, and Δ*sph*::*sph* strains (n = 5). **(D)** Survival rates of *Galleria mellonella* infected with *S. aureus* WT, Δ*sph*, and Δ*sph*::*sph* strains (n = 9). **(E, F)** Representative immunoblot and quantitative analysis of GPX4 expression in MAC-T cells infected with *S. aureus* WT, Δ*sph*, and Δ*sph*::*sph* strains for 6 h (n = 3). **(G-J)** Representative fluorescence images and **(K-N)** corresponding quantitative analyses of intracellular ROS (DCFH-DA; **G**, **K)**, Fe^2+^ accumulation **(H, L)**, lipid peroxidation (C11 BODIPY 581/591; **I**, **M)**, and mitochondrial membrane potential (JC-10; **J, N**) in MAC-T cells infected with the indicated *S. aureus* strains (n = 3). Scale bars, 200 μm or 100 μm. Data are presented as mean ± SD. *P < 0.05; **P < 0.01; ns, not significant.

### Sph induces ferroptosis in mammary epithelial cells

To functionally characterize the purified Sph protein, we first assessed its sphingomyelinase-associated hemolytic activity using an erythrocyte hemolysis assay. Treatment of erythrocytes with Sph (0.4 μg/mL) at 37°C for 1 h followed by incubation at 4°C for an additional 1 h induced pronounced hemolysis, confirming the biological activity of the purified protein. Critically, Sph-induced hemolysis was markedly suppressed by GW4869, supporting the involvement of sphingomyelinase activity in this process (S2A-F Fig). We next evaluated the cytotoxic potential of the purified Sph protein on MAC-T cells. As expected, treatment with Sph (0.5–32 μg/mL) exerted concentration- and time-dependent cytotoxicity (Fig 3A). Consistent with the characteristic “hot-cold” hemolytic activity of Sph, subsequent incubation at 4°C further enhanced Sph-induced cytotoxicity (Fig 3B). These findings indicate that the sphingomyelinase activity of Sph contributes substantially to membrane damage and cytotoxicity. Moreover, Sph elicited considerable cytotoxicity even in the absence of cold incubation, with this effect being further potentiated by subsequent cold exposure. This observation suggests that, in addition to its classical hot-cold hemolytic activity, Sph may engage additional mechanisms to promote mammary epithelial cell injury.

**Fig 3:**
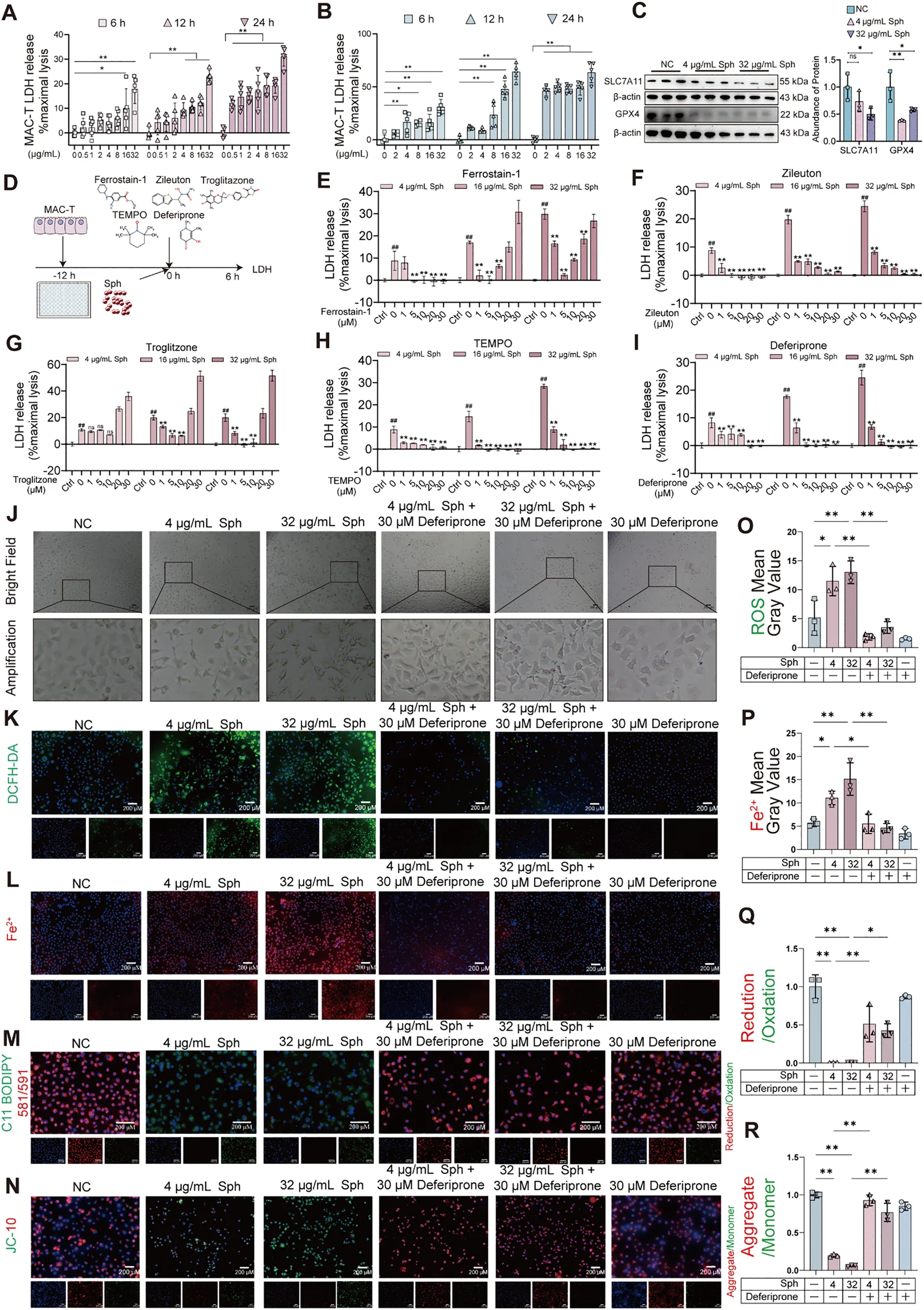
Recombinant Sph protein induces ferroptotic cell death, which is counteracted by ferroptosis inhibitors. **(A)** LDH release from MAC-T cells treated with the indicated concentrations of recombinant Sph for 6, 12, or 24 h under continuous incubation at 37°C (n = 5). **(B)** LDH release from MAC-T cells treated with recombinant Sph under the same conditions, followed by an additional 1-h incubation at 4°C (n = 5). **(C)** Representative immunoblots showing SLC7A11 and GPX4 expression in MAC-T cells treated with recombinant Sph (4 or 32 μg/mL) for 6 h, with corresponding quantitative analyses (n = 3). **(D)** Schematic diagram of the experimental protocol for ferroptosis inhibitor intervention in recombinant Sph-treated MAC-T cells. **(E-I)** LDH release from MAC-T cells treated with recombinant Sph (4, 16, or 32 μg/mL) in the presence of the indicated concentrations (0-30 μM) of ferrostatin-1 **(E)**, zileuton **(F)**, troglitazone **(G)**, TEMPO **(H)**, or deferiprone **(I)** (n = 3). ^##^*P* < 0.01 versus the control group; **P < 0.01 versus the Sph-treated group; ns, not significant. **(J)** Representative bright-field images of MAC-T cells following the indicated treatments. **(K-N)** Representative fluorescence images and **(O-R)** corresponding quantitative analyses of intracellular ROS (DCFH-DA; **K**, **O)**, Fe^2+^ accumulation **(L, P)**, lipid peroxidation (C11 BODIPY 581/591; **M**, **Q)**, and mitochondrial membrane potential (JC-10; **N, R**) in MAC-T cells treated with recombinant Sph (4 or 32 μg/mL) with or without deferiprone (30 μM) for 6 h (n = 3). Scale bars, 200 μm. Data are presented as mean ± SD. *P < 0.05; **P < 0.01; ns, not significant.

The established link between *S. aureus* infection and ferroptosis prompted us to interrogate a potential role for Sph in this form of cell death. Then, we examined the protein expression levels of the SLC7A11 and GPX4. Notably, Sph treatment markedly reduced the expression levels of both SLC7A11 and GPX4 in MAC-T cells (Fig 3C), suggesting disruption of the cellular antioxidant defense against ferroptosis. We next evaluated the cytoprotective effects of five pharmacological inhibitors targeting distinct ferroptosis-associated processes in Sph-treated cells (Fig 3D). Notably, the lipid peroxidation scavenger TEMPO and the iron chelator deferiprone effectively afforded pronounced protection against Sph-induced cytotoxicity, whereas ferrostatin-1, zileuton, and troglitazone exhibited comparatively weaker protective effects (Fig 3E-I). These findings further support the involvement of ferroptotic mechanisms in Sph-induced mammary epithelial cell injury. Notably, at higher concentrations, troglitazone tended to exacerbate Sph-induced cytotoxicity, consistent with the concentration-dependent effects observed in Fig 1H.

Furthermore, morphological assessment provided robust evidence to support these findings. Untreated MAC-T cells exhibited the characteristic polygonal, cobblestone morphology and formed confluent monolayers. In contrast, Sph stimulation triggered pronounced cellular elongation, loss of intercellular contacts, and extensive cell detachment, whereas co-treatment with deferiprone substantially alleviated these morphological alterations (Fig 3J). As expected, Sph treatment recapitulated the hallmark signatures of ferroptosis that were previously observed during *S. aureus* infection. Specifically, the significant accumulation of intracellular ROS (Fig 3K, O), an increase in labile Fe^2+^ pool (Fig 3L, P), elevated lipid peroxidation (Fig 3M, Q), and a loss of mitochondrial membrane potential (Fig 3N, R) induced by Sph treatment were observed. However, a concerted reversal of all these perturbations was markedly attenuated with deferiprone treatment (Fig 3K-R). In summary, our data establish that Sph acts as an inducer of ferroptosis in mammary epithelial cells.

### *S. aureus* Sph induces mtDNA release from mammary epithelial cells and activates cGAS-STING

Ferroptosis is characterized by profound mitochondrial alterations, including membrane densification with virtual disappearance of cristae [22]. To determine whether Sph contributes to these mitochondrial abnormalities, we first examined the ultrastructure of MAC-T cells by transmission electron microscopy. Compared with cells infected with the *S. aureus* Δ*sph* mutant, cells infected with WT *S. aureus* or the complemented Δ*sph*::*sph* strain exhibited pronounced mitochondrial fragmentation and vacuolization, increased electron density, and extensive disruption of cristae architecture, whereas the nuclear envelope remained largely intact (Fig 4A). Consistent with these observations, treatment with purified Sph induced similar mitochondrial fragmentation and cristae disruption (Fig 4C). Together, these findings support a critical role for Sph in *S. aureus*-induced mitochondrial structural damage. Given that severe mitochondrial structural disruption can compromise mitochondrial membrane integrity and facilitate the release of mitochondrial contents, we next investigated whether Sph-mediated mitochondrial injury could lead to the release of mtDNA. As predicted, infection with WT *S. aureus* or the Δ*sph*::*sph* strains, but not the Δ*sph* mutant, induced substantial mtDNA release, confirming the role of Sph in this process (Fig 4B). Consistently, immunofluorescence staining with an anti-DNA antibody revealed a pronounced accumulation of cytosolic DNA in Sph-treated cells (Fig 4D). The functional consequence of this structural instability was further evidenced by a sharp depletion of cellular ATP upon Sph challenge, indicating a critical collapse of mitochondrial function (Fig 4E).

**Fig 4:**
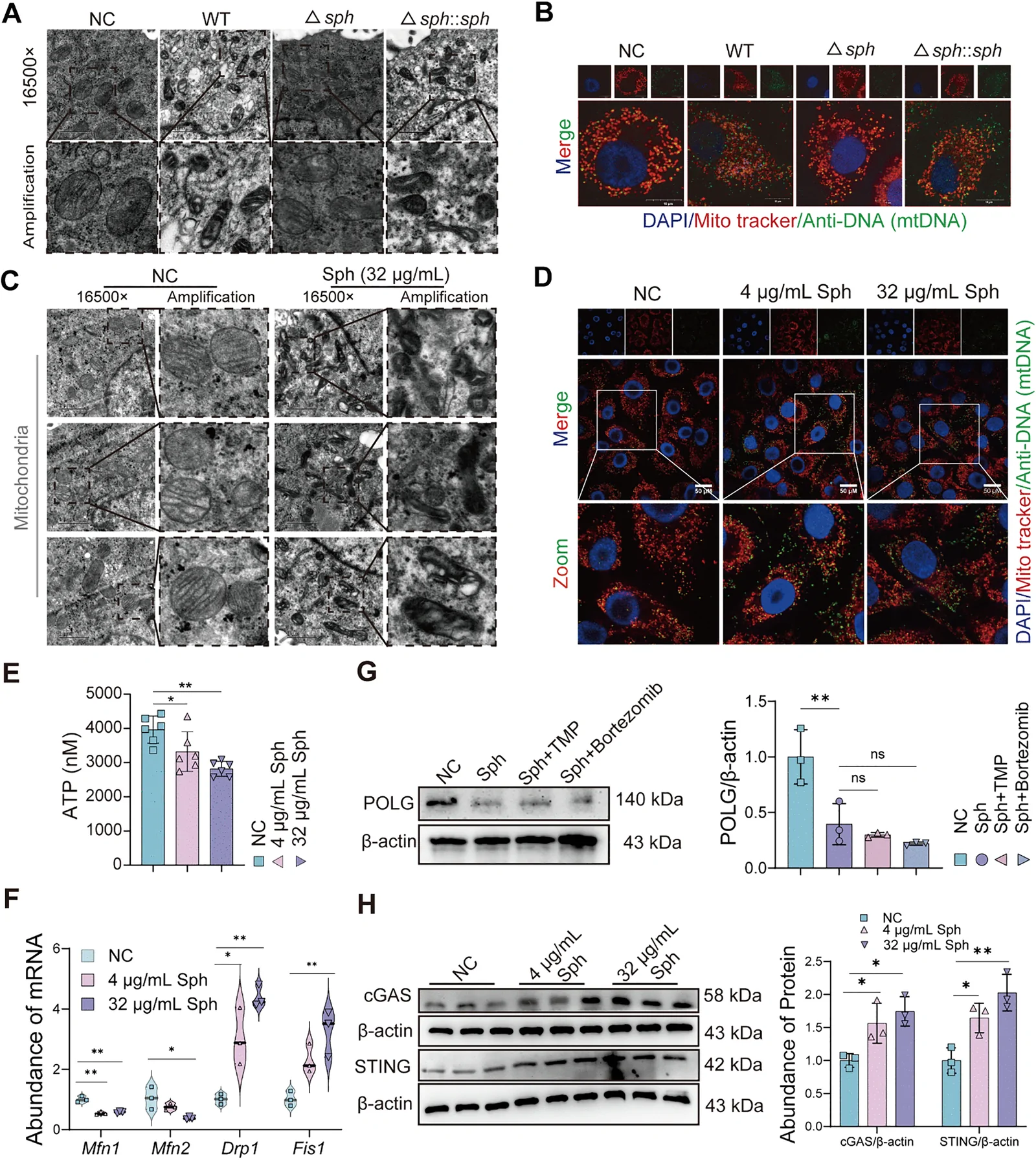
Sph causes mitochondrial fragmentation, cristae disruption, mtDNA release, and cGAS-STING pathway activation. **(A)** Representative transmission electron microscopy images showing mitochondrial ultrastructure in MAC-T cells infected with *S. aureus* WT, Δ*sph*, and Δ*sph*::*sph* (MOI = 200) for 6 h (n = 3). Original magnification, scale bars, 1 μm. **(B)** Representative confocal images showing cytosolic mtDNA release in MAC-T cells infected with the indicated *S. aureus* strains for 6 h (n = 3). Scale bars, 10 μm. **(C)** Representative transmission electron microscopy images of mitochondrial ultrastructure in MAC-T cells treated with recombinant Sph (32 μg/mL) for 6 h (n = 3). Scale bars, 1 μm. **(D)** Representative confocal images showing mtDNA release in MAC-T cells treated with recombinant Sph (4 or 32 μg/mL) for 6 h (n = 3). Scale bars, 50 μm. **(E)** Intracellular ATP levels in MAC-T cells treated with recombinant Sph (4 or 32 μg/mL) for 6 h (n = 6). **(F)** Relative mRNA expression of the mitochondrial fusion genes *Mfn1* and *Mfn2* and the fission-related genes *Drp1* and *Fis1* in MAC-T cells following Sph treatment (4 or 32 μg/mL, 6 h) (n = 3). Violin plots illustrate the distribution of mRNA levels across the experimental groups. **(G)** Expression of POLG protein in MAC-T cells treated with treated with recombinant Sph (32 μg/mL), TMP (2,3,5,6-tetramethylpyrazine; 10 μM), or bortezomib (500 nM) for 6 h (n = 3). **(H)** Representative immunoblots and quantitative analysis of cGAS-STING pathway proteins in MAC-T cells treated with recombinant Sph (4 or 32 μg/mL) for 6 h (n = 3). Data are presented as mean ± SD. *P < 0.05; **P < 0.01; ns, not significant.

To elucidate the mechanisms underlying Sph-induced mitochondrial fragmentation, we analyzed the expression of key regulators of mitochondrial fusion and fission. Sph treatment reduced the mRNA levels of the fusion-associated genes *Mfn1* and *Mfn2*, while concomitantly increasing those of the fission-associated genes *Drp1* and *Fis1* (Fig 4F), thereby shifting the dynamic equilibrium toward mitochondrial fragmentation. The expression level of POLG serves as a critical indicator of the mtDNA replication and repair capacity, and indirectly reflects the functional status of the TFAM-dependent regulatory axis [23]. Strikingly, Sph treatment induced a pronounced downregulation of POLG protein levels (Fig 4G). Notably, this loss of POLG could not be rescued by co-treatment with TMP (2,3,5,6-Tetramethylpyrazine), a known inhibitor of TFAM degradation, or by the proteasome inhibitor bortezomib (Fig 4G). Given that cytosolic mtDNA can activate cGAS and subsequently engage STING-dependent innate immune signaling [24], we next examined this signaling axis and found that Sph stimulation robustly activated the cGAS-STING signaling (Fig 4H). Collectively, these findings support a model in which Sph perturbs mitochondrial dynamics and mtDNA homeostasis, promotes cytosolic mtDNA accumulation, and consequently activates cGAS-STING signaling in mammary epithelial cells.

### Sph targets Mic60 to disrupt the MICOS complex

Mitochondrial cristae disruption has been implicated as an important trigger of cGAS-STING pathway activation. Notably, Mic60 (IMMT, MICOS60), a core component of the mitochondrial contact site and cristae organizing system (MICOS), serves as a critical hub for maintaining cristae structure and mediating substance exchange between the mitochondrial membranes [15]. Furthermore, to investigate the interaction between Sph and the host MICOS complex, Co-IP assays were employed and demonstrated a robust interaction between Mic60 and both wild-type Sph and its enzymatically inactive mutants, T_149_A (defective in DNA-binding) and H_150_N (devoid of sphingomyelinase activity) (Fig 5A-C). This interaction was functionally corroborated by the mitochondrial co-localization of transfected eGFP-Sph in NIH/3T3 and HEK-293T cells (Fig 5J). To further define the Mic60 region responsible for Sph binding, we generated a series of truncated His-Mic60 variants (Fig 5D) and examined their interaction with Sph by Co-IP. Notably, all Mic60 variants except the Mic60_201–327_ (comprising amino acids 201-327) were detected in the immunoprecipitated complexes (Fig 5E-I).

**Fig 5:**
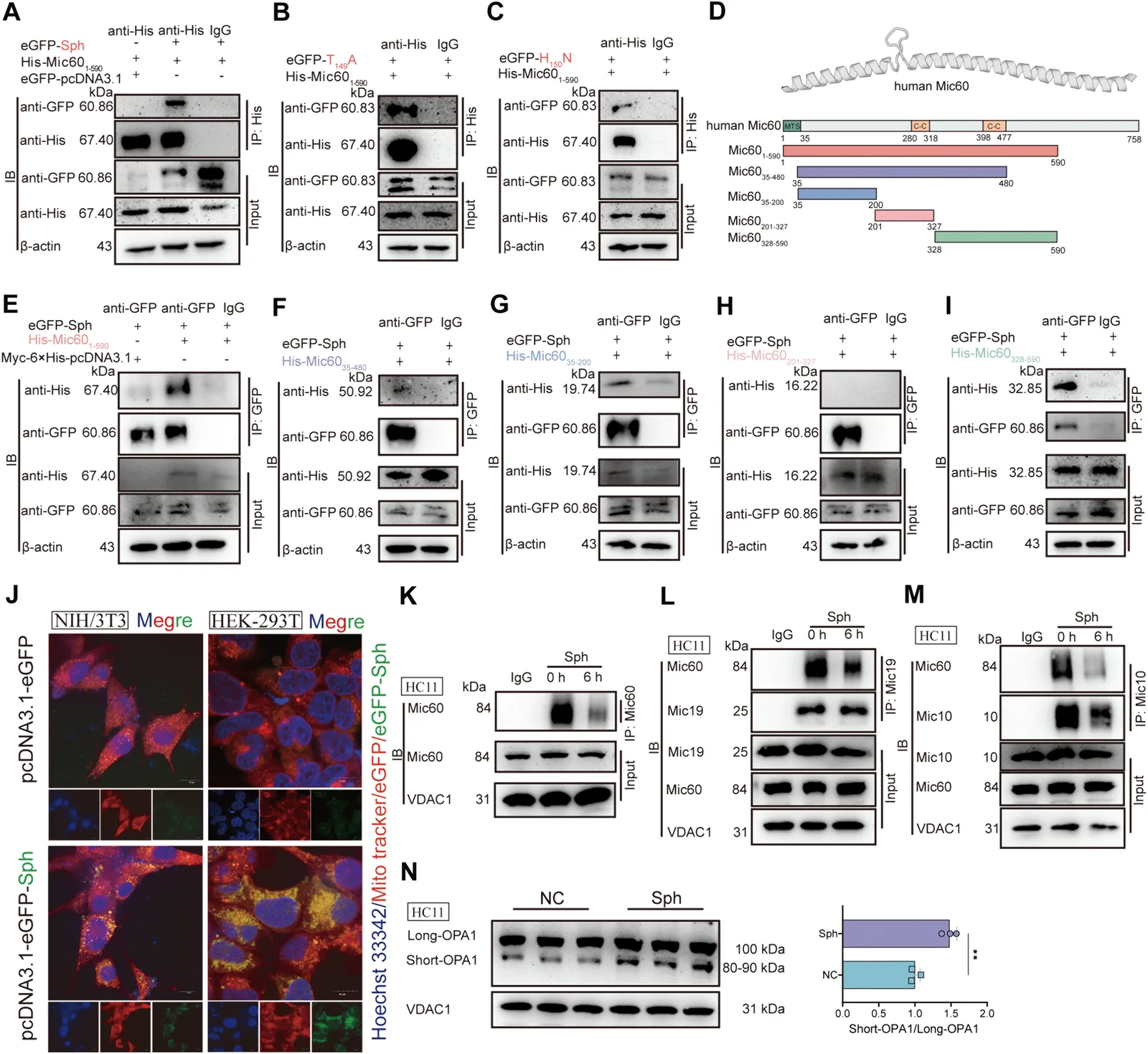
Sph targets Mic60 and disrupts MICOS organization and mitochondrial cristae homeostasis. **(A-C)** Co-immunoprecipitation of His-Mic60_1-590_ with eGFP-Sph, eGFP-T_149_A, or eGFP-H_150_N in co-transfected NIH/3T3 cells using an anti-His antibody. **(D)** Schematic representation of Mic60 and the truncated Mic60 constructs used for domain-mapping analyses. **(E-I)** Co-immunoprecipitation analysis of eGFP-Sph with His-Mic60_1-590_, His-Mic60_35-480_, His-Mic60_35-200_, His-Mic60_201-327_, or His-Mic60_328-590_ in co-transfected NIH/3T3 cells using an anti-GFP antibody. **(J)** Representative fluorescence images showing mitochondrial localization of eGFP-Sph in NIH/3T3 and HEK-293T cells. Scale bars, 10 μm. **(K)** Immunoprecipitation of endogenous Mic60 from HC11 cells treated with recombinant Sph (32 μg/mL) for 6 h using an anti-Mic60 antibody. **(L, M)** Co-immunoprecipitation analysis of Mic60 association with Mic19 **(L)** or Mic10 **(M)** in HC11 cells following recombinant Sph treatment (32 μg/mL, 6 h), using anti-Mic19 or anti-Mic10 antibodies, respectively. **(N)** Representative immunoblot showing the relative abundance of long and short OPA1 isoforms in HC11 cells treated with recombinant Sph (32 μg/mL) for 6 h. Data are presented as mean ± SD. **P < 0.01.

To further test the hypothesis that Sph targets Mic60 to disrupt mitochondrial homeostasis, we overexpressed the full-length human Flag-tagged Mic60 (1–757 aa) protein in NIH/3T3 cells, followed by treatment with recombinant Sph protein. Overexpression of Mic60 alone did not activate cGAS (S3A-C Fig), induce obvious alterations in mitochondrial morphology, or promote mtDNA release (S3D, S3E Fig). Notably, Mic60 overexpression markedly attenuated Sph-induced mitochondrial damage, reduced the cytosolic release of mtDNA, and diminished cGAS activation (S3B-E Fig). These findings support a critical role for Mic60 in mediating Sph-induced mitochondrial dysfunction.

The mitochondrial cristae architecture is critically maintained by the MICOS complex. The core MICOS subunits, including the scaffold protein Mic60, the adapter protein Mic19, and the membrane-shaping protein Mic10, form a stable assembly that is essential for inner membrane integrity. At crista junctions, Mic60 serves as a central organizing hub that recruits Mic19, which in turn facilitates the association of Mic10 with the complex. This hierarchical organization is critical for MICOS stability and function [25]. To investigate whether Sph disrupts mitochondrial integrity by targeting this complex, we first examined its interaction with Mic60. Co-IP assays in HC11 cells revealed that treatment with Sph markedly reduced the recovery of Mic60 when immunoprecipitated with a Mic60-specific antibody (Fig 5K). This finding suggests that Sph binding may alter Mic60 conformation or accessibility to the antibody, although additional mechanisms cannot be excluded. We next examined whether Sph affects the association of Mic60 with other core MICOS components. Notably, following Sph treatment, Co-IP using antibodies against either Mic19 or Mic10 revealed a marked reduction in their association with Mic60 (Fig 5L, M). These results indicate that Sph disrupts the interaction of Mic60 with key MICOS components, thereby destabilizing the organization of the core MICOS complex.

Consistent with MICOS disruption, Sph treatment induced aberrant processing of the dynamin-like GTPase OPA1, characterized by a shift from long OPA1 to short OPA1 isoforms, a molecular event associated with impaired cristae architecture and mitochondrial stress (Fig 5N). In summary, these findings reveal that Sph triggers the loss of mitochondrial cristae and disruption of mitochondrial dynamics through interaction with Mic60, an essential constituent of the MICOS. Specifically, the regions Mic60_1–200_ and Mic60_328–590_ in Mic60 contribute critically to Sph binding. This interaction may therefore represent an important molecular event underlying Sph-induced mitochondrial structural damage and its pathogenic effects.

### *S. aureus* Sph activates STING to promote GPX4 degradation and ferroptosis

The degradation of GPX4 protein, a key suppressor of phospholipid peroxidation, constitutes a pivotal event precipitating ferroptotic cell death. Our investigation revealed that the STING inhibitor C-176 [26] effectively antagonized GPX4 depletion induced by Sph, prompting us to interrogate the mechanistic link between STING signaling and GPX4 protein stability (Fig 6A). We therefore hypothesized that STING signaling may promote GPX4 degradation by engaging the cellular autophagy-lysosomal machinery. Strikingly, we observed that Sph treatment markedly increased LC3B II accumulation (Fig 6B), indicating altered autophagic activity. We next examined whether lysosomal degradation contributes to Sph-induced GPX4 loss. Critically, co-treatment with either NH_4_Cl or chloroquine, both of which impair lysosomal function by disrupting lysosomal acidification, substantially restored GPX4 protein levels following Sph treatment (Fig 6C). These findings implicate the lysosomal degradation pathway in Sph-induced GPX4 depletion.

**Fig 6:**
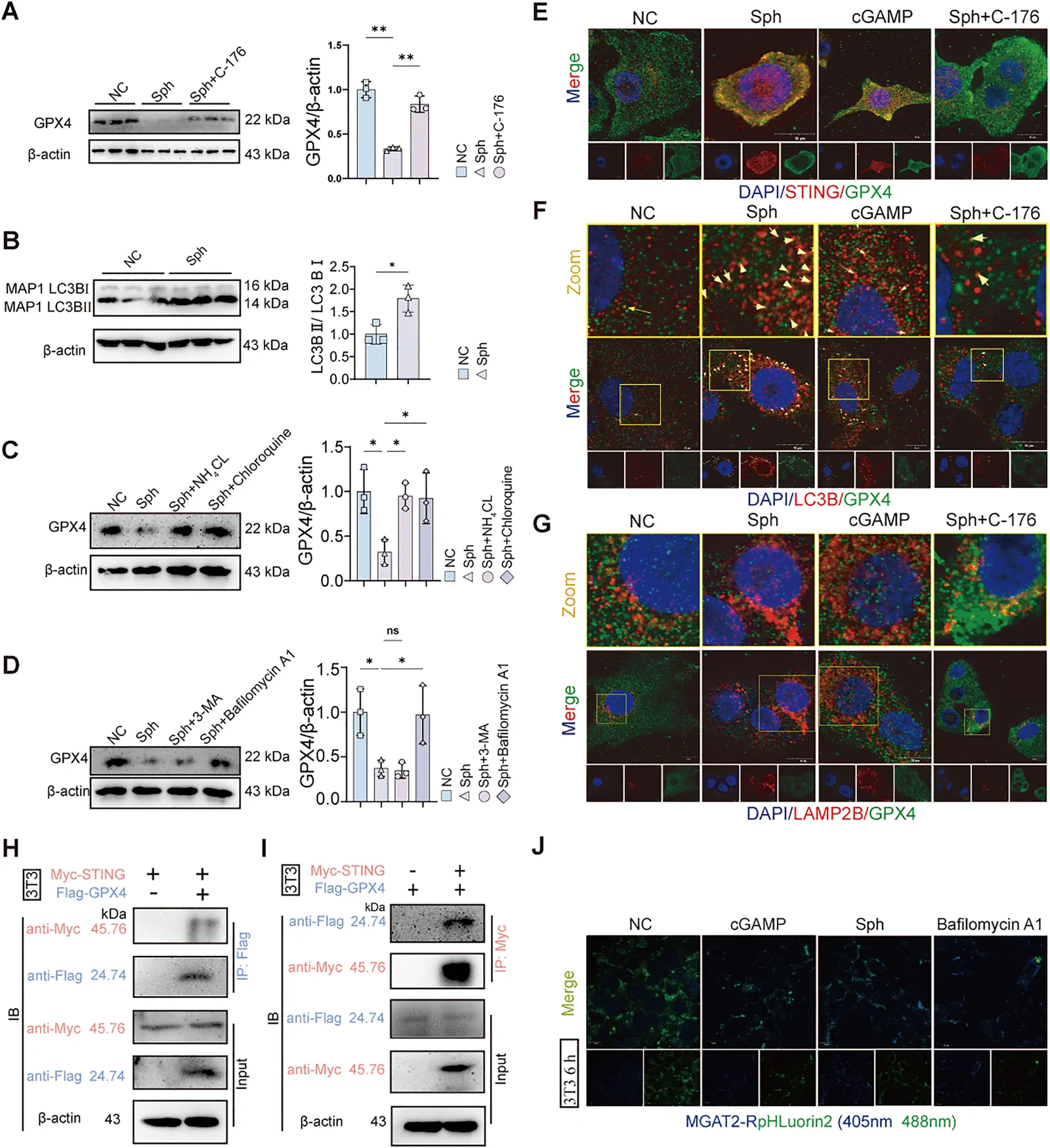
*S. aureus* Sph activates STING to promote autophagy-lysosome-dependent GPX4 degradation and ferroptosis. **(A)** Expression of GPX4 protein in HC11 cells treated with 32 μg/mL recombinant Sph and C-176 (5 μM) for 6 h. **(B)** Expression of of LC3B-I and LC3B-II levels in HC11 cells treated with recombinant Sph (32 μg/mL) for 6 h. **(C)** GPX4 expression in HC11 cells treated with recombinant Sph (32 μg/mL) alone or in combination with NH_4_Cl (10 mM) or chloroquine (20 μM) for 6 h. **(D)** Expression of GPX4 protein in HC11 cells treated with 32 μg/mL recombinant Sph alone or in combination with 3-MA (5 mM) or bafilomycin A1 (100 nM) for 6 h. **(E-G)** Representative confocal images and corresponding colocalization analyses of STING with GPX4 **(E)**, LC3B with GPX4 **(F)**, and LAMP2B with GPX4 **(G)** in HC11 cells treated with recombinant Sph (32 μg/mL), cGAMP (2 μg/mL), or C-176 (5 μM), as indicated, for 6 h. Scale bars, 10 μm. **(H, I)** Reciprocal co-immunoprecipitation analysis of Flag-GPX4 and Myc-STING in co-transfected NIH/3T3 cells using anti-Flag (H) or anti-Myc **(I)** antibodies. **(J)** Ratiometric imaging of Golgi luminal pH in NIH/3T3 cells expressing MGAT2-RpHLuorin2 following treatment with cGAMP (2 μg/mL) or bafilomycin A1 (100 nM). The 405/488 nm fluorescence ratio was used as an indicator of Golgi pH (MGAT2-RpHLuorin2). Data are presented as mean ± SD (n = 3). *P < 0.05; **P < 0.01; ns, not significant.

To further investigate the involvement of autophagic processing, we employed pharmacological inhibitors targeting distinct stages of the autophagy pathway. Bafilomycin A1, which inhibits vacuolar H^+^-ATPase activity and thereby disrupts lysosomal acidification and autophagosome-lysosome fusion, effectively prevented Sph-induced GPX4 loss. In contrast, 3-methyladenine (3-MA), an inhibitor of class III PI3K commonly used to suppress early autophagosome formation, failed to restore GPX4 protein levels (Fig 6D). Together, these findings suggest that Sph-induced STING activation promotes GPX4 degradation through a lysosome-dependent process involving the autophagy machinery, with the late degradative stage playing a particularly important role. Loss of GPX4 would consequently compromise cellular defenses against phospholipid peroxidation and thereby facilitate ferroptotic cell death.

Given that STING can induce autophagy independently of the type I interferon (IFN-I) response [18,27,28], we explored the mechanism by which Sph triggers STING-dependent GPX4 degradation. Upon Sph stimulation, STING exhibited markedly increased colocalization with GPX4, a phenotype similarly observed following treatment with the canonical STING agonist cGAMP. Critically, this induced proximity between STING and GPX4 was effectively attenuated by the STING inhibitor C-176 (Fig 6E), suggesting that STING activation promotes the spatial association between STING and GPX4. Given the established role of activated STING in engaging LC3-associated autophagic machinery [29] and previous evidence supporting the formation of a STING/GPX4/LC3B complex [21], we hypothesized that activated STING may facilitate the recruitment of GPX4 to the autophagic machinery. Supporting this model, both Sph and cGAMP stimulation robustly enhanced the colocalization of GPX4 with LC3B, whereas C-176 substantially attenuated this effect (Fig 6F). Collectively, these results support that STING serves as a critical intermediary linking GPX4 to the LC3B-associated autophagic machinery, thereby facilitating the autophagic degradation of GPX4.

We next sought to trace the ultimate fate of GPX4 following its recruitment to LC3B-associated autophagic machinery. During autophagosome maturation, lipidated LC3B-II associates with autophagosomal membranes, and subsequent fusion of autophagosomes with lysosomes results in the formation of autolysosomes. Notably, Sph treatment markedly increased the colocalization of GPX4 with LAMP2B, a lysosomal membrane-associated protein (Fig 6G), supporting the lysosomal trafficking of GPX4 following Sph stimulation.

To biochemically validate the physical interaction between STING and GPX4, we performed a series of Co-IP assays. We first sought to establish a direct interaction in a controlled overexpression system. In NIH/3T3 cells co-expressing Myc-tagged STING and Flag-tagged GPX4, reciprocal Co-IP experiments confirmed a robust association: immunoprecipitation of Myc-STING efficiently co-precipitated Flag-GPX4, whereas reciprocal immunoprecipitation of Flag-GPX4 co-precipitated Myc-STING (Fig 6H, I). This bidirectional validation unequivocally demonstrates that STING and GPX4 can form a molecular complex in a heterologous expression context.

STING-mediated non-canonical autophagy has been linked to its proton channel activity during trafficking from the endoplasmic reticulum to the ERGIC and Golgi compartments. Following activation and translocation, STING can alter proton homeostasis within these membranes, resulting in an increase in luminal pH. This perturbation in organelle acidity is sensed by the vacuolar H^+^-ATPase (V-ATPase), which undergoes increased assembly of its V1 and V0 sectors and subsequently promotes recruitment of ATG16L1, thereby facilitating LC3B lipidation [30,31]. To determine whether Sph induces a similar alteration in Golgi pH, we transfected NIH/3T3 cells with MGAT2-RpHLuorin2, a genetically encoded ratiometric probe that enables dynamic monitoring of pH within the cis- and medial-Golgi compartments. An increased 405/488-nm fluorescence ratio reflects a higher luminal pH [32]. Notably, Sph stimulation induced marked Golgi deacidification, comparable to that observed following treatment with cGAMP or bafilomycin A1, a V-ATPase inhibitor that increases organellar luminal pH (Fig 6J).

To investigate the role of STING in Sph-induced autophagic degradation of GPX4, we depleted STING in NIH/3T3 cells using siRNA (S4B Fig). STING knockdown significantly attenuated Sph-induced LDH release (S4A Fig) and markedly preserved GPX4 protein levels following Sph treatment (S4B, S4C Fig). Concurrently, we examined ferroptosis-related phenotypes in STING-depleted 3T3 cells. STING knockdown substantially blunted the Sph-induced increases in intracellular ROS levels (S4D, S4E Fig), Fe^2+^ accumulation (S4F, S4G Fig), and lipid peroxidation (S4H, S4I Fig).

Furthermore, to determine the functional role of GPX4 in Sph-induced ferroptotic cell injury, we overexpressed GPX4 in NIH/3T3 cells (S5B, S5C Fig). Upon Sph stimulation, GPX4 overexpression significantly attenuated Sph-induced LDH release (S5A Fig). More importantly, GPX4 overexpression alleviated the Sph-induced increases in intracellular ROS levels (S5D, S5E Fig), Fe^2+^ levels accumulation (S5F, S5G Fig), and lipid peroxidation (S5H, S5I Fig) in 3T3 cells.

Collectively, these data delineate a coherent spatiotemporal pathway in which Sph-induced STING activation promotes the association of GPX4 with STING and LC3B-associated autophagic machinery, thereby facilitating the recruitment of GPX4 into the autophagy-lysosomal degradation pathway. Together, these results establish a mechanistic link between Sph-induced STING activation and autophagy-lysosome-dependent GPX4 degradation, thereby providing a molecular basis for the ensuing ferroptotic response.

### Deficiency of Sph significantly attenuates *S. aureus*-induced mastitis in mice

To investigate the pathophysiological significance of Sph-induced ferroptosis *in vivo*, we established a murine mastitis model and administered the iron chelator deferiprone to assess its protective effect. Macroscopic examination and H&E staining of the mammary glands revealed that infection with wild-type (WT) *S. aureus* and the complemented Δ*sph*::*sph* resulted in pronounced swelling and extensive architectural disruption of the mammary tissue, whereas the Δ*sph* mutant elicited markedly attenuated pathological damage (Fig 7A, B). Concordantly, deferiprone conferred effective protection against infections caused by both the *S. aureus* WT and *S. aureus* Δ*sph*::*sph* strains. Additionally, bacterial burden in the mammary glands was significantly elevated in mice infected with the WT and Δ*sph*::*sph* strains, an effect that was reversed by the Δ*sph* mutant (Fig 7C), indicating that Sph promotes bacterial persistence or colonization within mammary tissue. Furthermore, the levels of TNF-α and IL-6 were substantially increased in the WT and Δ*sph*::*sph* infection groups (Fig 7D, E), underscoring a more robust systemic inflammatory response.

**Fig 7:**
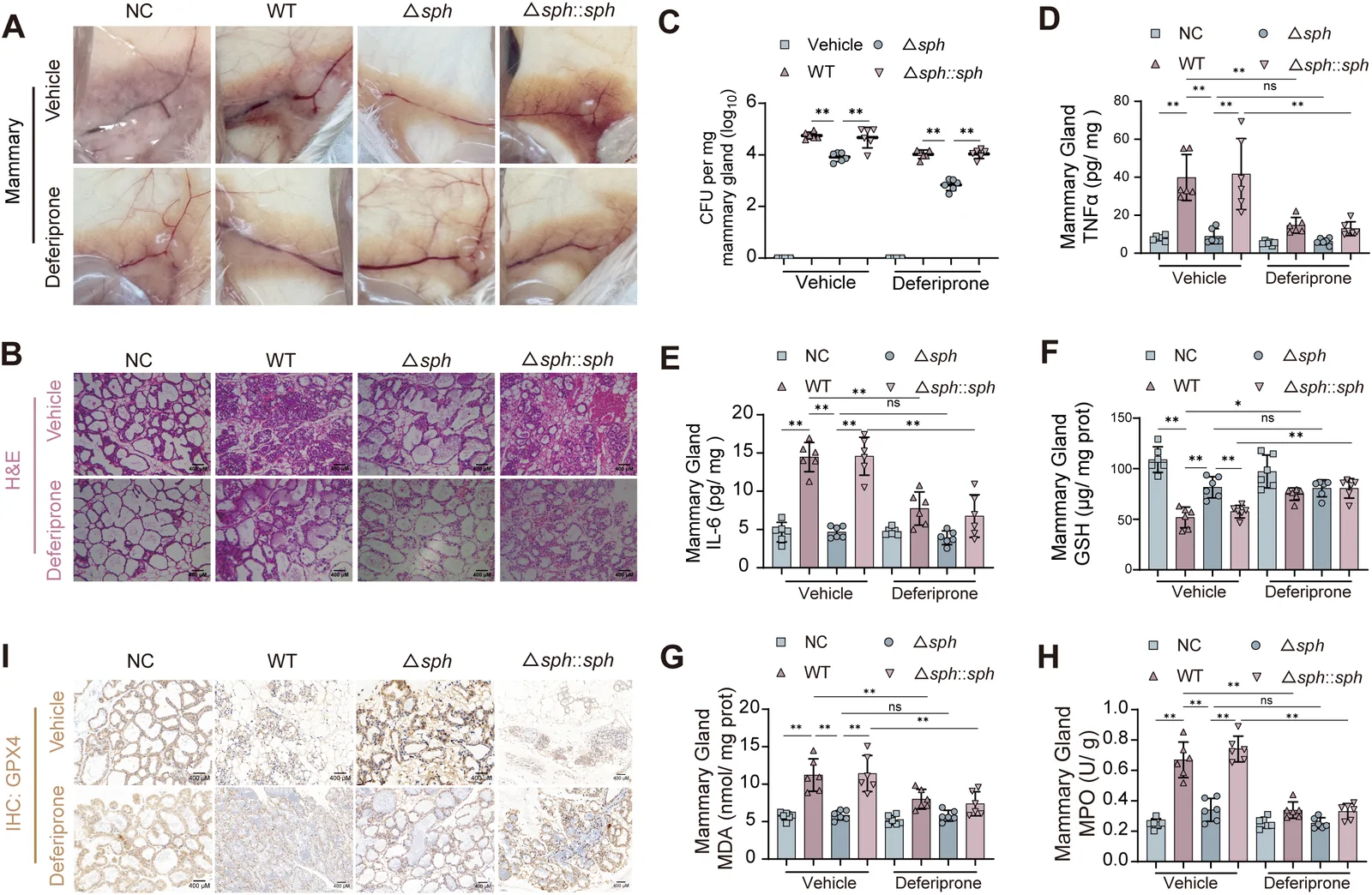
Sph deficiency attenuates *S. aureus*-induced mastitis and ferroptotic tissue injury in mice. Mice were intramammarily inoculated through mammary gland with 5 × 10⁶ CFU of *S. aureus* WT, Δ*sph*, or Δ*sph::sph* strains. Infected mice received deferiprone (50 mg/kg) or vehicle by oral gavage every 8 h for 48 h (n = 6 per group). **(A)** Gross morphology of mammary glands. **(B)** Representative H&E-stained sections of mammary tissues. Scale bars, 400 μm. **(C)** Bacterial burdens in mammary tissues. **(D, E)** TNF-α **(D)** and IL-6 **(E)** levels in mammary tissue homogenates. **(F)** GSH levels in mammary tissues. **(G)** MDA levels in mammary tissues. **(H)** MPO activity in mammary tissues. **(I)** Representative immunohistochemical staining of GPX4 in mammary tissues. Scale bars, 400 μm. Data are presented as mean ± SD. *P < 0.05; **P < 0.01; ns, not significant.

To establish a causal link between these pathologies and ferroptosis, we examined several biochemical and molecular markers associated with ferroptosis. Compared with infection by WT *S. aureus* or the complemented Δ*sph*::*sph* strain, infection with the Sph-deficient Δ*sph* mutant markedly attenuated GSH depletion and reduced the accumulation of the lipid peroxidation product malondialdehyde (MDA) (Fig 7F, G). Myeloperoxidase (MPO) was also markedly reduced in the *S. aureus* Δ*sph* group compared to the WT and Δ*sph*::*sph* infection groups (Fig 7H). Critically, immunohistochemical (IHC) analysis demonstrated reduced expression levels of GPX4 in mammary tissues infected with either WT *S. aureus* or Δ*sph*::*sph* strain; whereas GPX4 expression was largely preserved in tissues infected with the Δ*sph* mutant (Fig 7I). Collectively, our data delineate a critical role for Sph in driving ferroptosis within the host mammary gland, as indicated by the depleted GPX4 levels and elevated lipid peroxidation. These findings support an important contribution of Sph-induced ferroptotic injury to the pathogenesis of *S. aureus* mastitis.

## Discussion

Investigating the dissemination pathways of pathogens is as critical as studying their intracellular survival for a complete understanding of microbial pathogenesis. Ferroptosis, an iron-dependent form of regulated cell death driven by excessive lipid peroxidation, has increasingly been implicated in tissue injury and, in certain infectious contexts, may facilitate pathogen dissemination [33–35]. However, the specific microbial effectors that manipulate host ferroptotic pathways and the molecular mechanisms by which they contribute to tissue damage and pathogen spread remain poorly understood. Emerging evidence also implicates ferroptosis in the pathogenesis of mastitis, particularly during *S. aureus* infection. Upon *S. aureus* invasion, bacterial virulence factors and the ensuing inflammatory response can promote excessive ROS production, perturb iron homeostasis, and impair cellular antioxidant defenses. Concurrent depletion of GSH and dysfunction of the GPX4-dependent antioxidant system favor the accumulation of phospholipid peroxides, thereby promoting ferroptotic death of mammary epithelial cells and aggravating tissue injury [36,37]. Here, we identify the *S. aureu*s toxin Sph as a previously unrecognized effector that promotes ferroptotic injury during mastitis. Previous study revealed that Sph promotes *S. aureus* biofilm formation through its DNA-binding activity, while its enzymatic activities as a sphingomyelinase and phosphocholine-specific phosphodiesterase contribute to pathogenesis by modulating host inflammation and increasing infection severity [10,11]. In the present study, we reveal a novel mechanism that *S. aureus* toxin Sph functions as a mitochondrial disruptor by targeting the mitochondrial cristae-organizing protein Mic60, and promotes mtDNA release into the cytosol. This liberated mtDNA activates the cGAS-STING pathway, leading to STING-dependent autophagy-lysosomal degradation of GPX4, a key suppressor of ferroptosis, and ultimately promoting ferroptotic cell death.

Mitochondria are important regulators of ferroptosis, particularly under conditions in which mitochondrial metabolism and redox homeostasis are actively engaged, such as erastin-induced ferroptosis [22]. Recent research highlights that mitochondrial dysfunction, triggered by cellular stress, initiates the release of mtDNA, and cytosolic mtDNA serves as a potent ligand for the cGAS-STING pathway [38]. Beyond its canonical role in inducing type I interferon and inflammatory responses, cGAS-STING signaling is increasingly recognized as an important regulator of cellular homeostasis, including autophagy [39,40]. Accumulating evidence indicates that bacterial toxins can exploit mitochondrial vulnerability through distinct mechanisms. For example, bacterial phospholipase C has been reported to impair mtDNA repair and alter mtDNA methylation, thereby driving mitochondrial energy metabolism dysfunction [41]. Recent study showed that *Salmonella* genotoxin CdtB can enter the mitochondrial matrix and interact with mtDNA, and then disrupt mitochondrial DNA integrity and activates STING, thereby promoting a proinflammatory senescence-associated secretory phenotype (SASP) [17]. Furthermore, *Helicobacter hepaticus* CdtB translocates to the mitochondrial inner membrane via Hsp90 and interacts with ATP5A1, thereby impairing mitochondrial function and promotes the release of mtDNA [42], identifying a toxin‑driven mtDNA release pathway independent of direct mtDNA interaction. Together, these studies illustrate that bacterial toxins can trigger mtDNA release either by directly damaging the mitochondrial genome or by disrupting mitochondrial structure and function.

*S. aureus* Sph exhibits both sphingomyelinase and phosphodiesterase activities. Although Sph belongs to the DNase I superfamily and exhibits DNA-binding capacity, it lacks appreciable nuclease activity toward double-stranded DNA [9]. We therefore investigated whether Sph disrupts mitochondrial homeostasis by targeting the MICOS complex. This concept is supported by recent evidence showing that the *Listeria monocytogenes* pore-forming toxin listeriolysin O (LLO) interacts with Mic60 and promotes mitochondrial fragmentation, highlighting Mic60 as a host target exploited by bacterial pathogens to perturb mitochondrial dynamics [16]. Intriguingly, our results identify Mic60 as a host target of Sph and further suggest that regions outside Mic60_201–327_ contribute to Sph binding. Functionally, Sph impaired the association of Mic60 with Mic19 and Mic10, indicating destabilization of MICOS organization. Because Mic19 participates in coupling Mic60 to the Mic10-containing membrane-shaping module, disruption of Mic60-Mic19 association is expected to compromise Mic10 incorporation and thereby impair MICOS-dependent cristae organization [25,43,44]. Consistent with this model, Sph induced pronounced cristae disruption accompanied by mtDNA release into the cytosol. This signifies that the architectural integrity of the cristae is not only critical for energy conversion but also acts as a vital barrier sequestering mtDNA. The liberated mtDNA is subsequently sensed by the cytosolic cGAS-STING pathway, thereby igniting a potent innate immune response [15]. Our work thus positions Sph-induced MICOS disruption as an upstream pathogenic event that directly bridges mitochondrial structural catastrophe to the initiation of STING-dependent inflammation, a mechanism with broad implications for infection and sterile inflammatory diseases. Gram-positive bacterial infections can activate both canonical autophagy and STING-induced type I-IFN responses [45]. Subsequent studies have revealed an evolutionarily conserved role of cGAS-STING signaling in autophagy that is independent of type I interferon production. Upon activation, STING traffics from the endoplasmic reticulum to the ER-Golgi intermediate compartment (ERGIC) and Golgi, where STING-containing membranes can support LC3 lipidation and autophagy-related membrane remodeling [18]. GPX4, a central suppressor of ferroptosis, is subject to multiple modes of protein turnover, including proteasomal degradation and lysosome-dependent degradation under specific stress conditions [13,46]. In our study, Sph-induced STING activation was accompanied by GPX4 loss and increased LC3-II accumulation. Notably, pharmacological inhibition of lysosomal acidification or late autophagic processing with bafilomycin A1, NH_4_Cl, or chloroquine markedly preserved GPX4 levels, whereas 3-MA failed to provide comparable protection. These findings support a model in which Sph-activated STING promotes GPX4 degradation through a lysosome-dependent autophagic pathway, with the late degradative stage playing a particularly important role.

The functional interplay between STING and GPX4 has recently garnered significant attention. Initial studies established that GPX4-maintained redox homeostasis facilitates STING activation [47], while subsequent work revealed that activated STING can promote autophagy-dependent GPX4 degradation and thereby facilitate ferroptosis under mitochondrial stress and in neuronal models [19,20]. A seminal study by Wang et al. provided mechanistic insight into this process, showing that mitochondrial stress promotes STING-GPX4 association and trafficking through ERGIC-associated vesicular compartments toward LC3-positive autophagic structures, ultimately resulting in lysosomal GPX4 degradation [21]. In this study, we observed that Sph-induced STING activation promoted LC3-dependent trafficking of GPX4 to lysosomes for degradation. We therefore propose a model in which Sph-mediated disruption of the Mic60/MICOS complex induces mitochondrial cristae damage and mtDNA release, leading to activation of the cGAS-STING pathway. Activated STING subsequently facilitates the engagement of GPX4 with LC3-associated autophagic machinery and its delivery to lysosomal compartments for degradation. Together, these findings uncover a previously unrecognized virulence mechanism by which *S. aureus* couples mitochondrial structural damage to STING-dependent GPX4 degradation and ferroptotic tissue injury.

### Conclusions

In summary, during *S. aureus*-induced mastitis, Sph targets Mic60 to disrupt the MICOS complex stability, leading to mitochondrial cristae disorganization and impaired mitochondrial dynamics. The ensuing release of mtDNA activates the cGAS-STING pathway, which promotes autophagy-lysosome-dependent degradation of GPX4. Loss of GPX4 compromises cellular defenses against phospholipid peroxidation, thereby driving lipid oxidative damage and ferroptotic death of mammary epithelial cells. This pathogenic cascade contributes to *S. aureus*-induced mammary tissue injury (Fig 8). Collectively, these findings define a previously unrecognized Sph-driven pathogenic mechanism and identify Sph as a potential therapeutic target for disease intervention.

**Fig 8:**
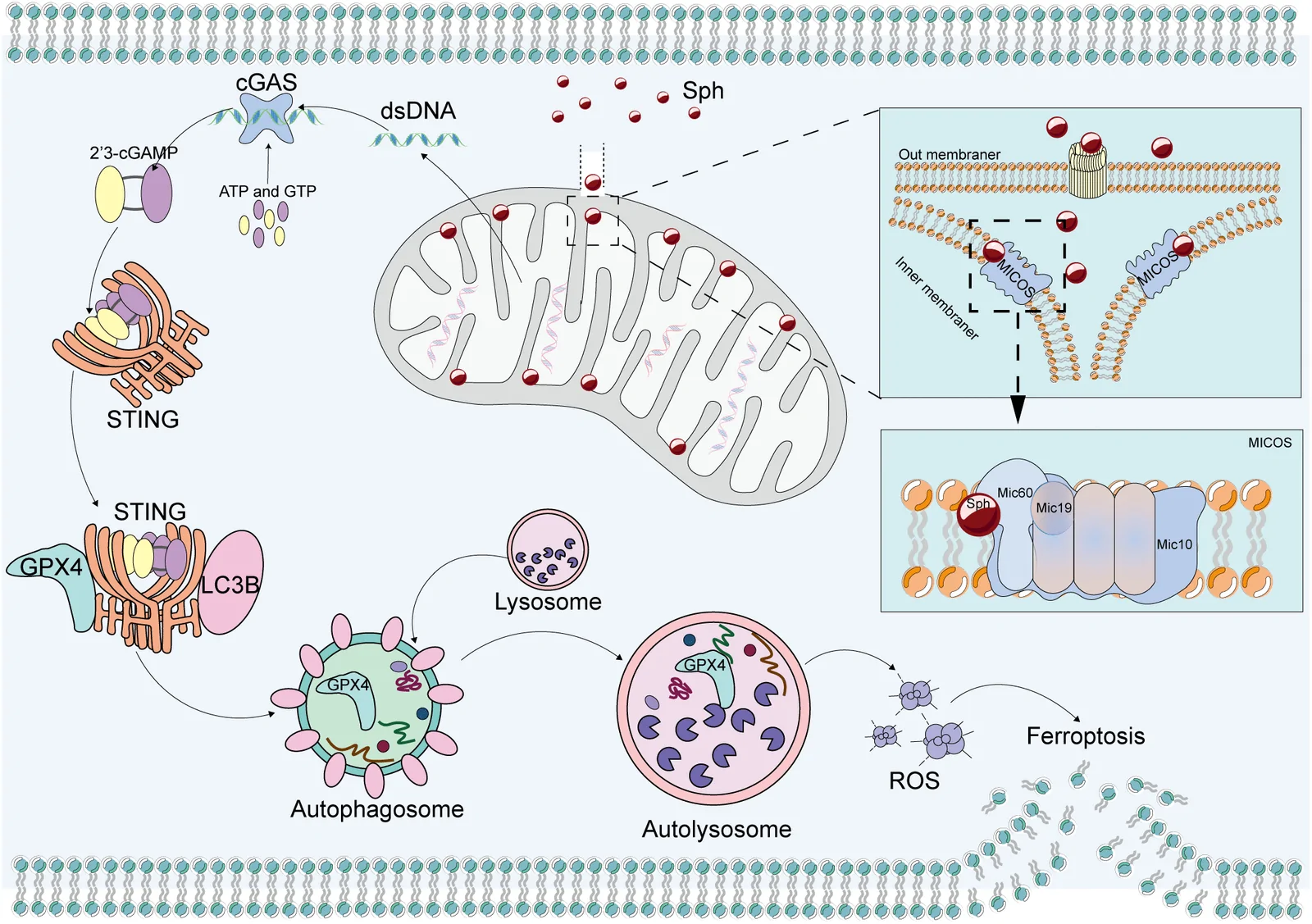
Proposed model of Sph-induced ferroptosis in mammary epithelial cells. The figure was created entirely from scratch by the authors using Adobe Illustrator. Sph targets Mic60 and destabilizes MICOS organization, thereby disrupting mitochondrial cristae architecture and mitochondrial dynamics and promoting mtDNA release. Cytosolic mtDNA activates the cGAS-STING pathway, after which activated STING facilitates recruitment of GPX4 to LC3-associated autophagic machinery and its subsequent lysosomal degradation. Loss of GPX4 drives lipid peroxidation, and culminates in ferroptotic death of mammary epithelial cells.

## Materials and methods

### Ethics statement

All animal procedures were approved by the Institutional Animal Care and Use Committee of Jilin University (Approval No: SY202508044) and conducted in accordance with the guidelines of the U.S. National Institutes of Health (NIH) for the humane care and use of laboratory animals.

### Experimental reagents, plasmids and antibodies

Ferrostatin-1 (Aladdin, Shanghai CN); Zileuton (TargetMol; Shanghai CN); TEMPO (Aladdin; Shanghai CN); Troglitazone (Bepharm; Shanghai CN); Deferiprone (Bepharm; Shanghai CN); GW4869 (Aladdin; Shanghai CN); Tetramethylpyrazine (Yuanye; Shanghai CN); Bortezomib (MERYER; Shanghai CN); NH_4_Cl (TargetMol; Shanghai CN); Chloroquine (Chemxyz; Shanghai CN); 3-MA (MERYER; Shanghai CN); Bafilomycin A1 (TargetMol; Shanghai CN); cGAMP (Bepharm; Shanghai CN); C-176 (Adamas; Shanghai CN).

SLC7A11 (Abclonal; Wuhan CN); GPX4 (Rabbit: Immunoway; Jiangsu CN) (Mouse: Boster; CA USA); β-actin (Immunoway; Jiangsu CN); STING (Affinity; Jiangsu CN); cGAS (Abclonal; Wuhan CN); POLG (Affinity; Jiangsu CN); VDAC1 (Affinity; Jiangsu CN); Mic60 (Affinity; Jiangsu CN); Mic19 (Immunoway; Jiangsu CN); Mic10 (Immunoway; Jiangsu CN); OPA1 (Immunoway; Jiangsu CN); Mouse His-Tag (Abmart; Shanghai CN); Mouse GFP-Tag (Abmart; Shanghai CN); Mouse Myc-Tag (Abmart; Shanghai CN); Mouse Flag-Tag (Abmart; Shanghai CN); Rabbit His-Tag (Epizyme; Shanghai CN); Rabbit GFP-Tag (Epizyme; Shanghai CN); Rabbit Myc-Tag (Epizyme; Shanghai CN); Rabbit Flag-Tag (Epizyme; Shanghai CN); Anti-DNA mouse monoclonal (Progen; HDB GER); LC3B (CST; MA USA); LAMP2B (Abcam; Shanghai CN). HRP Rabbit IgG (Fc specific) secondary antibody (Epizyme; Shanghai CN); Rabbit IgG HRP (Affinity; Jiangsu CN); Mouse IgG HRP (Affinity; Jiangsu CN). Multicolor prestained protein Ladder (Epizyme; Shanghai CN). ECL Protein Marker (Bioss; Beijing CN).

pET-28a (+) (Laboratory Stock). pCasSA [48]. pLI50, pCMV-GPX4–3 × FLAG-SECIS-Neo, pCMV-T7-MCS-3 × FLAG-Neo, pCMV-STING1–3 × Myc-Neo, pCMV-T7-IMMT-3 × Flag-Neo were purchased from MIAOLING PLASMID. pcDNA3.1-Myc-6 × His and pcDNA3.1-eGFP were purchased from Comate Bioscience Co.,Ltd (Jilin, CN). pcDNA3.1-eGFP-sph, pcDNA3.1-eGFP-T_149_A and pcDNA3.1-eGFP-H_150_N synthesized by Comatebio Bioscience Co.,Ltd (Jilin, CN). pcDNA3.1-Myc-6 × His-Mic60_1–590_ and pcDNA3.1-Myc-6 × His-Mic60_35–480_ were synthesized by Sangon Biotech (Shanghai, CN) Co., Ltd. pcDNA3.1-Myc-6 × His-Mic60_35–200_, pcDNA3.1-Myc-6 × His-Mic60_201–327_ and pcDNA3.1-Myc-6 × His-Mic60_328–590_ each was constructed using primers “Mic60_35-200_F/R, Mic60_201-327_F/R and Mic60_328-590_F/R”. MGAT2-RpHLorin2 (Addgene).

The molecular weight of the protein was then predicted using the NovoPro online protein molecular weight calculation tool.

### Cell culture

MAC-T and HC11 cells were obtained from American Type Culture Collection (ATCC; CRL-10274 and CRL-3062, respectively). The cells were cultured in F-12 medium (Gibco, CN) supplemented with 10% fetal bovine serum (FBS; Umedium, Hefei, CN), 1% insulin-transferrin-selenium (ITS; M&C Gene Technology, Shanghai, CN) and 1% penicillin/streptomycin (NEST Biotechnology, Wuxi, CN). NIH/3T3 (ATCC; CRL-1658) and HEK-293T (ATCC; CRL-3216) cells were cultured in Dulbecco’s modified Eagle’s medium (DMEM) supplemented with 10% FBS and 1% penicillin–streptomycin. All cells were maintained at 37°C in a humidified atmosphere containing 5% CO_2_.

### Bacterial strains and culture conditions

All bacterial strains used in this study are listed in Table A in S1 Appendix. For routine cultivation, *S. aureus* ATCC25923 (*S. aureus* WT strain) was grown in Tryptic Soy Broth (TSB; Hopebio HB4114–19).

The *S. aureus* Δ*sph* mutant was constructed using a CRISPR/Cas9-based gene-editing system as previously described [48]. For genetic complementation, the native *sph* gene was cloned into the shuttle vector pLI50 and introduced into the Δ*sph* mutant by electroporation to generate the complemented strain, Δ*sph*::*sph*. Appropriate antibiotics, including ampicillin, kanamycin, and chloramphenicol, were used as required for plasmid selection and maintenance.

In addition, *E. coli* strains DH5α and BL21(DE3) were employed for plasmid propagation and recombinant Sph protein expression, respectively. For recombinant Sph production, the *sph* coding sequence was cloned into the pET-28a expression vector and expressed in *E. coli* BL21(DE3) as previously described [49]. All primers used in this study are presented in Table B in S1 Appendix.

### LDH assay

MAC-T cells were seeded into 96-well plates at a density of 5 × 10⁴ cells per well and allowed to adhere before treatment. Cells were then infected with *S. aureus* WT, Δ*sph*, or Δ*sph*::*sph* strains at the indicated multiplicities of infection (MOIs), or treated with recombinant Sph protein for the indicated durations. Additionally, to assess the protective effects of ferroptosis inhibitors, cells were treated with the indicated inhibitors in the presence of *S. aureus* or recombinant Sph for 6 h. Positive and negative controls were included in parallel. Cytotoxicity was quantified by measuring lactate dehydrogenase (LDH) release using a commercial LDH Cytotoxicity Assay Kit (Beyotime; Shanghai CN) according to the manufacturer’s instructions.

### Assessment of ferroptosis-associated cellular changes

After infection with *S. aureus* strains or stimulation with recombinant Sph protein at 37°C for 6 h, cells were incubated with fluorescent probes to assess ferroptosis-associated oxidative stress, intracellular Fe^2+^ accumulation, lipid peroxidation, and mitochondrial membrane potential. Intracellular ROS were detected using DCFH-DA (1 μM; TargetMol), Fe^2+^ levels were assessed using a live-cell fluorescent probe FerroOrange (1:1000; Meilunbio), lipid peroxidation was evaluated using C11 BODIPY 581/591 (10 μM; Maokangbio), and mitochondrial membrane potential was monitored using JC-10 (1:1000; Meilunbio). All probes were diluted in serum-free medium and applied according to the manufacturers’ instructions. Fluorescence images were acquired using a fluorescence microscope under identical imaging conditions for all experimental groups.

### Hemolytic activity assay

Fresh erythrocytes (2.5% v/v) were treated with various concentrations of recombinant Sph protein at 37°C for 1 h, followed by incubation at 4°C for an additional 1 h. Double-distilled water (ddH_2_O) and phosphate-buffered saline (PBS) were used as positive and negative controls, respectively. After incubation, samples were centrifuged to remove intact cells and cellular debris, and the absorbance of the supernatants was measured at the appropriate wavelength. Hemolytic activity was calculated as: Hemolysis (%) = [(A_sample_ - A_negative_)/(A_positive_ - A_negative_)] × 100. A_sample_, A_negative_, and A_positive_ represent the absorbance values of the Sph-treated sample, PBS control, and ddH_2_O control, respectively.

### Western blot analysis

Protein extraction was performed with RIPA Lysis Buffer containing phosphatase inhibitor cocktail and phenylmethylsulfonyl fluoride (PMSF). After centrifugation, the supernatant protein concentration was quantified using a BCA assay. Equal amounts of protein (20 µg) were separated by 10% or 12.5% SDS-PAGE and transferred onto polyvinylidene difluoride (PVDF) membranes (Merck Millipore; IPVH00010/ISEQ00010). Membranes were blocked with 5% non-fat milk in TBST and incubated with the indicated primary antibodies overnight at 4°C. After washing with TBST, membranes were probed with horseradish peroxidase (HRP)-conjugated secondary antibodies. Protein signals were visualized using an enhanced chemiluminescence (ECL) substrate and captured with a Tanon 4200 imaging system. Band intensities were quantified by densitometric analysis and normalized to the corresponding loading control.

### Growth curve analysis

Overnight cultures of *S. aureus* WT, Δ*sph*, and Δ*sph*::*sph* strains were diluted into fresh tryptic soy broth (TSB) and incubated at 37°C with shaking. Then, bacterial growth was monitored at 600 nm at 1-h intervals using a spectrophotometer (UNIC UV-2000).

### Biofilm formation assay

The bacterial suspension (1 × 10^6^ CFU per well) was incubated for 24 h. After incubation for 24 h at 37°C under static conditions, non-adherent cells were removed and the wells were gently washed with PBS. The attached biofilms were stained with 0.1% crystal violet for 1 h. The retained crystal violet was then solubilized in ethanol, and biofilm biomass was quantified by measuring the absorbance at 600 nm.

### *Galleria mellonella* survival assay

Overnight cultures of *S. aureus* WT, Δ*sph*, and Δ*sph*::*sph* strains were harvested by centrifugation, washed, and resuspended in sterile PBS. The bacterial suspensions were adjusted to the same concentration, and 10 μL containing 1 × 10^6^ CFU was injected into the last left proleg of each *Galleria mellonella* larva using a microsyringe. Subsequently, the larvae were placed at 37°C to monitor and record the survival of larvae every 12 h.

### ATP levels determination

Intracellular ATP levels were quantified using a commercial ATP Assay Kit (Beyotime; Shanghai, CN). Briefly, following treatment with the recombinant Sph protein, MAC-T cells were lysed on ice with ATP lysis buffer, and the clarified supernatants were then harvested for ATP determination. The luminescence (RLU) of different samples was measured using a luminometer (Biotek Synergy LX).

### Quantitative reverse transcription-PCR

Total RNA was extracted using TRIzol reagent and reverse-transcribed into complementary DNA (cDNA) using the M5 HiPer First Strand cDNA Synthesis Kit (Mei5bio, CN). Quantitative reverse transcription PCR (qRT-PCR) was performed using 2 × M5 HiPer Real-Time PCR Mix (Mei5bio, China). Relative mRNA expression levels were normalized to β-actin and calculated using the 2^(–ΔΔCt) method. Primer sequences used in this study are listed in Table B in S1 Appendix.

### Transmission electron microscopy

MAC-T cells were collected and fixed with 4% glutaraldehyde for 12 h. Following fixation, samples were dehydrated through a graded ethanol series, infiltrated, and embedded in resin. Ultrathin sections (50 nm in thickness) were prepared and stained with uranyl acetate and lead citrate to enhance ultrastructural contrast. The ultrastructural features of mitochondria were examined using transmission electron microscopy (TEM) (Hitachi High-Tech HT7800).

### Immunofluorescence staining

For immunofluorescence staining, cells grown on coverslips or in confocal dishes were fixed with 4% paraformaldehyde and permeabilized using 0.1% Triton X-100. After blocking non-specific binding sites with 5% goat serum, samples were incubated overnight at 4°C with primary antibodies, followed by incubation with appropriate fluorophore-conjugated secondary antibodies (1:1000; Abbkine) for 1 h at room temperature in the dark. Where indicated, mitochondria were pre-labeled with MitoTracker (Beyotime; Shanghai, CN) before fixation and immunostaining. After washing with PBS, samples were mounted in antifade medium and imaged using a laser scanning confocal microscope.

### Mitochondrial isolation

Mitochondria were isolated from treated cells using a Cell Mitochondria Isolation Kit (Beyotime; Shanghai, CN). In short, cells were harvested and homogenized in the provided mitochondrial isolation buffer. The homogenates were first subjected to low-speed centrifugation to remove nuclei, unbroken cells, and cellular debris. The resulting supernatants were subsequently centrifuged at higher speed to pellet the mitochondrial fraction. The mitochondrial pellets were collected and resuspended in the appropriate buffer for further analyses.

### Cell transfection

The NIH/3T3 or HEK-293T cells (1 × 10^6^ cells per well) were used for transfection at the appropriate confluence using Lipofectamine 3000 together with P3000 reagent (Thermo Fisher Scientific; Shanghai, CN), according to the manufacturer’s instructions. For GPX4 expression, the transfection medium was supplemented with ITS (containing selenium; M&C Gene Technology; Shanghai, CN). After 6 h, the medium was replaced with DMEM to further culture for 24 h.

### CO-immunoprecipitation (Co-IP) Assay

Co-immunoprecipitation assays were performed using a commercial Co-IP kit (Beyotime; Shanghai, CN). Briefly, treated cells were lysed in the recommended immunoprecipitation buffer and clarified by centrifugation. An aliquot of each lysate was retained as the input control, while the remaining lysate was incubated with the indicated primary antibody, followed by Protein A/G magnetic beads to capture the immune complexes. After extensive washing to remove nonspecifically bound proteins, the bead-bound complexes were eluted by boiling in SDS sample buffer and analyzed by Western blotting. To ensure blotting specificity, species-mismatched and/or Fc-specific secondary antibodies were employed.

### Animal experiments

Female BALB/c mice aged 8–10 weeks were obtained from Liaoning Changsheng Biotechnology (Liaoning, CN) and housed under SPF conditions with a 12-h light/dark cycle, with free access to standard chow and water. Following a one-week acclimatization period, the mice were randomly assigned into experimental groups (n = 6 per group).

For establishment of the lactating murine mastitis model, female mice were housed with males for mating and provided with a breeding diet. Pregnant females were subsequently housed individually, and lactating dams approximately 7 days postpartum were used for infection experiments. Mice were anesthetized, and the distal teat region was gently exposed. Bacterial suspension containing 5 × 10⁶ CFU of *S. aureus* was slowly inoculated into the designated mammary gland using a microsyringe. Deferiprone was administered by oral gavage at 50 mg/kg every 8 h.

At 48 h post-infection, mice were anesthetized and subsequently euthanized. Mammary tissues were collected. Portions of each tissue sample were fixed for histopathological and immunohistochemical analyses, processed for biochemical assays, or snap-frozen and stored at −80°C for subsequent analyses.

### H&E staining and immunohistochemical staining

Mammary tissues were fixed in 4% paraformaldehyde, paraffin-embedded, and sectioned at 8 μm. For histological analysis, tissue sections were deparaffinized, rehydrated, and stained with hematoxylin and eosin (H&E) according to standard procedures. For immunohistochemistry (IHC) analysis, paraffin sections were deparaffinized and rehydrated, followed by antigen retrieval in citrate buffer and blocking of nonspecific binding. Sections were then incubated with the indicated primary antibodies, followed by appropriate horseradish peroxidase (HRP)-conjugated secondary antibodies. Immunoreactivity was visualized using 3,3′-diaminobenzidine (DAB) as the chromogenic substrate, and then examined using a light microscope.

### Bacterial colonization determination

Freshly excised mammary tissues were weighed and homogenized in sterile buffer. After diluting the homogenate sample in a series of steps, it was inoculated onto TSB medium. After 24 h of incubation at 37°C, viable bacteria were enumerated by colony counting and expressed as colony-forming units (CFU) per gram of tissue.

### Measurement of tissue GSH, MDA and MPO levels

Mammary tissues were weighed and homogenized in the appropriate assay buffers. According to the manufacturers’ instructions, the levels of glutathione (GSH; Solarbio; Beijing, CN), malondialdehyde (MDA; Jiancheng Bioengineering Institute; Nanjing, CN), and myeloperoxidase (MPO; Jiancheng Bioengineering Institute; Nanjing, CN) were measured using respective commercial assay kits.

### Measurement of TNF-α and IL-6 levels

Inflammatory cytokines in mouse mammary tissue homogenates were quantified using commercial ELISA kits (Biolegend, Beijing, CN) according to the manufacturer’s instructions. Absorbance was measured using a microplate reader, and cytokine concentrations were calculated from standard curves.

### Data and statistical analysis

All experiments were independently repeated at least three times. Data are presented as the mean ± standard deviation. Statistical analyses were performed using GraphPad Prism 10 software. Comparisons between two groups were performed using a two-tailed unpaired Student’s *t*-test. For comparisons involving three or more groups, one-way analysis of variance (ANOVA) was conducted, followed by Tukey’s post hoc test for multiple comparisons. For datasets with two independent variables, two-way ANOVA was performed followed by Tukey’s post hoc test for multiple comparisons. Differences were considered statistically significant (* or ^#^) at *P* < 0.05 and highly significant (** or ^##^) at *P* < 0.01.

## Supporting information

S1 Fig Construction and validation of the *S. aureus* Δ*sph* mutant and Δ*sph*::*sph* complemented strain. (A) PCR identification of the *sph* locus and 16S rRNA in WT and Δ*sph* strains using the indicated primer sets. (B) PCR confirmation of *sph* in *S. aureus* WT, Δ*sph*, and Δ*sph*::*sph* strains. (TIF)

S2 Fig Expression, purification, and functional validation of recombinant Sph protein. (A) PCR amplification of the *sph* coding sequence from *S. aureus* ATCC25923. (B) PCR verification of *sph* in *E. coli* DH5α (pET-28a(+)-*sph*)*.* (C) Double-digestion analysis confirming correct construction of pET-28a(+)-*sph* in *E. coli* DH5α. (D) Imidazole-gradient elution of recombinant Sph protein following affinity purification. (E) Validation of the hemolytic activity of recombinant Sph protein. (F) Inhibitory effect of GW4869 on the hemolytic activity of recombinant Sph. Data are presented as mean ± SD. (TIF)

S3 Fig Mic60 overexpression attenuates Sph-induced mitochondrial damage and cGAS activation. (A) Verification of Flag-tagged Mic60 overexpression in NIH/3T3 cells. (B, C) Representative immunoblot and quantitative analysis of cGAS expression in NIH/3T3 cells treated with recombinant Sph (32 μg/mL) for 6 h in the presence or absence of Mic60 overexpression (n = 3). (D) Representative transmission electron microscopy images showing mitochondrial ultrastructure in NIH/3T3 cells following recombinant Sph treatment (32 μg/mL, 6 h) with or without Mic60 overexpression (n = 3). Scale bars, 1 μm. (E) Representative confocal images showing mtDNA release in NIH/3T3 cells treated with recombinant Sph for 6 h in the presence or absence of Mic60 overexpression (n = 3). Scale bars, 10 μm. Data are presented as mean SD. **P < 0.01. (TIF)

S4 Fig Knockdown of STING attenuates Sph-induced GPX4 depletion and ferroptotic injury. NIH/3T3 cells were transfected with control or STING-targeting siRNA (si-STING) and subsequently treated with recombinant Sph for 6 h. (A) LDH release from the indicated treatment groups (n = 3). (B, C) Representative immunoblots and quantitative analyses of STING and GPX4 protein levels (n = 3). (D-I) Representative fluorescence images and corresponding quantitative analyses of intracellular ROS (DCFH-DA; D, E), Fe^2+^ accumulation (F, G), and lipid peroxidation (C11 BODIPY 581/591; H, I) in the indicated treatment groups (n = 3). Scale bars, 200 μm. Data are presented as mean ± SD. (TIF)

S5 Fig Overexpression of GPX4 to validate its role in Sph-induced ferroptosis. NIH/3T3 cells were transfected with Flag-GPX4 or the corresponding control vector and subsequently treated with recombinant Sph for 6 h. (A) LDH release from the indicated treatment groups (n = 3). (B, C) Representative immunoblot and quantitative analysis of GPX4 protein expression (n = 3). (D-I) Representative fluorescence images and corresponding quantitative analyses of intracellular ROS (DCFH-DA; D, E), Fe^2+^ accumulation (F, G), and lipid peroxidation (C11 BODIPY 581/591; H, I) in the indicated treatment groups (n = 3). Scale bars, 200 μm. Data are presented as mean SD. **P < 0.01. (TIF)

S1 Appendix Table A. Bacterial strains used in this study. Table B. Primer Sequence Information. (DOCX)

S1 Rawgel Original uncropped and unadjusted blot and gel images. (PDF)

S1 File Source date. (XLSX)

S2 File Graphical abstract. (DOCX)
