# Supplementary material for: Sph-mediated mtDNA release and autophagy-dependent ferroptosis contribute to Staphylococcus aureus-induced mastitis
Source: PLoS Pathog. 2026 Sep 29;22(9):e1014644. doi: 10.1371/journal.ppat.1014644 (PMC13645233; doi:10.1371/journal.ppat.1014644)

Fig 2 E

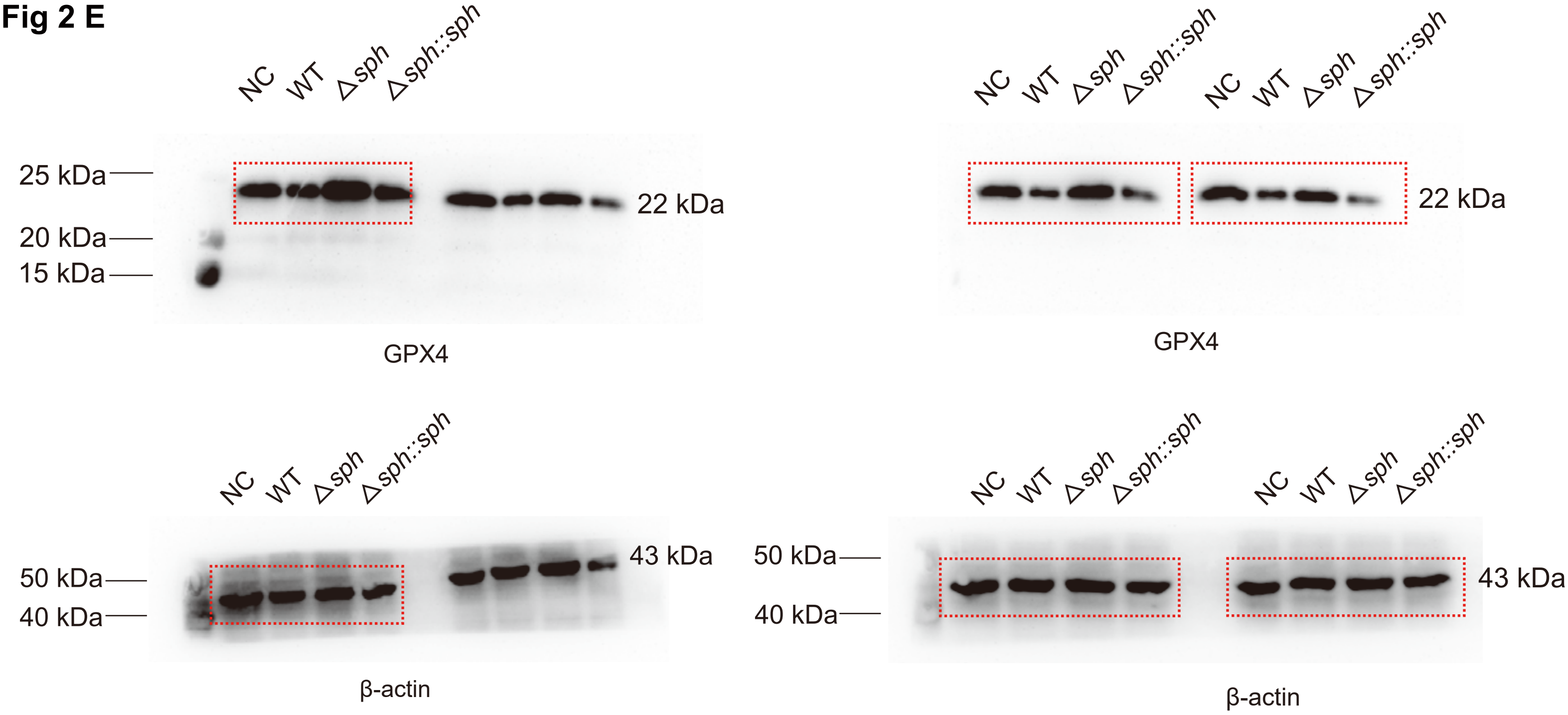
NC
WT
△sph
△sph::sph
25 kDa
20 kDa
15 kDa
22 kDa
GPX4
NC
WT
△sph
△sph::sph
NC
WT
△sph
△sph::sph
22 kDa
GPX4
NC
WT
△sph
△sph::sph
50 kDa
40 kDa
43 kDa
β-actin
NC
WT
△sph
△sph::sph
NC
WT
△sph
△sph::sph
50 kDa
40 kDa
43 kDa
β-actin

Fig 3 C

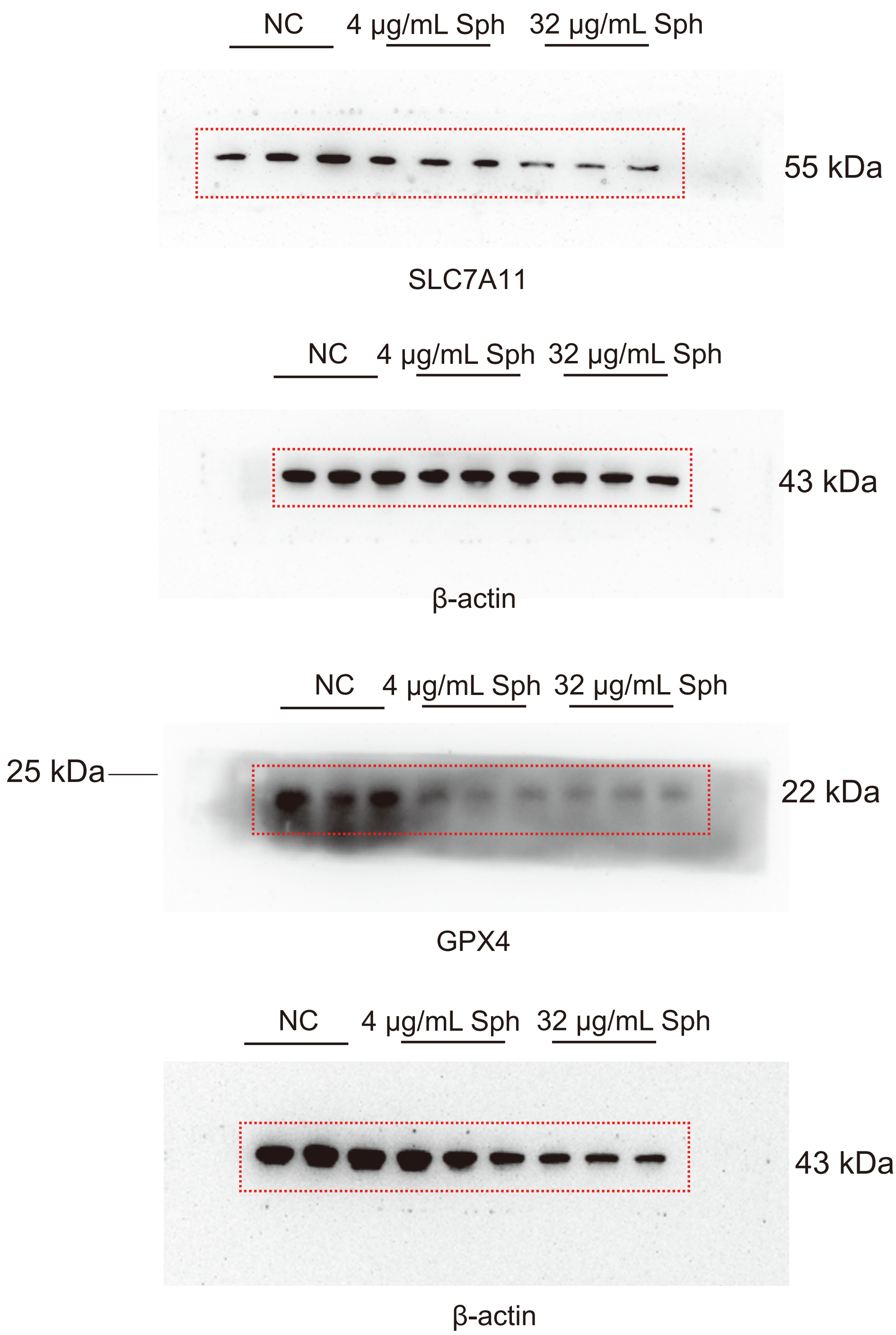
NC
4 μg/mL Sph
32 μg/mL Sph
55 kDa
SLC7A11
NC
4 μg/mL Sph
32 μg/mL Sph
43 kDa
β-actin
NC
4 μg/mL Sph
32 μg/mL Sph
25 kDa
22 kDa
GPX4
NC
4 μg/mL Sph
32 μg/mL Sph
43 kDa
β-actin

Fig 4 G

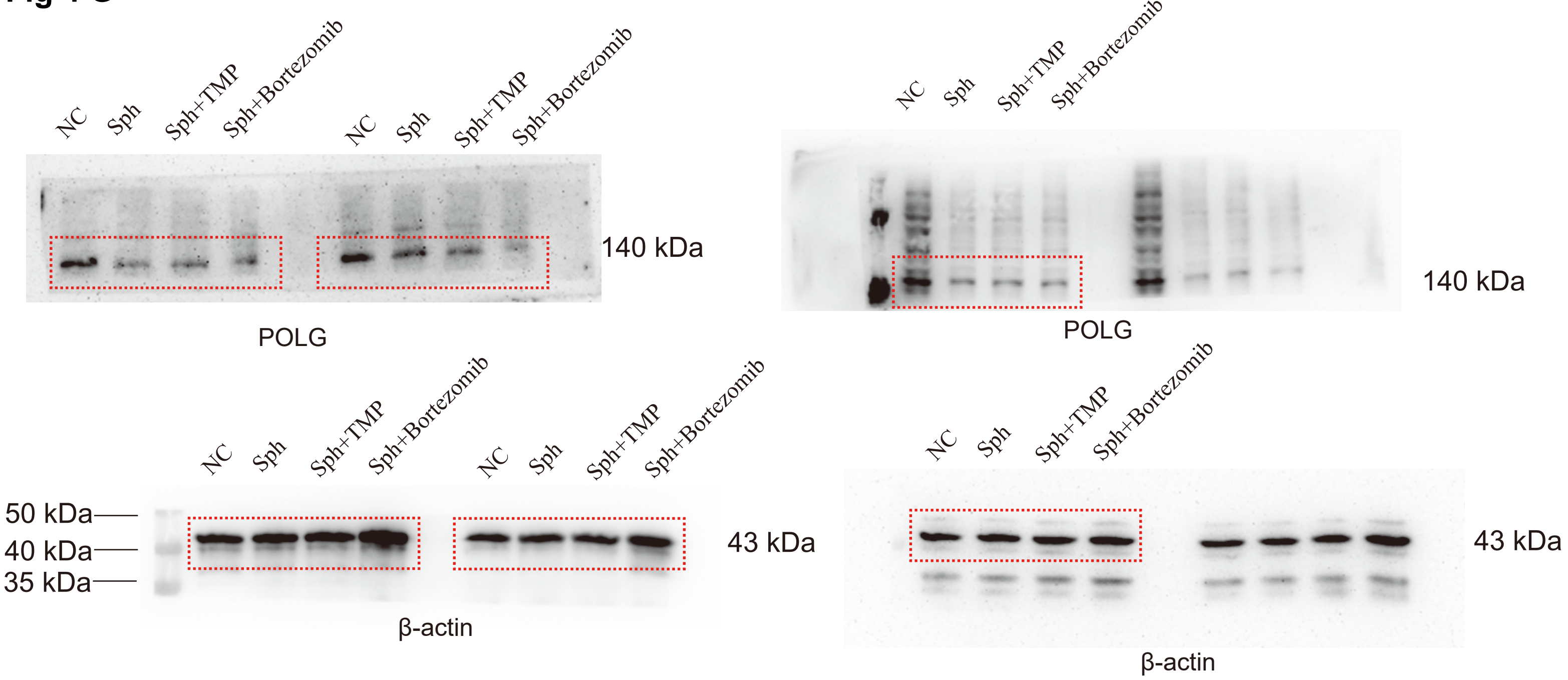
NC
Sph
Sph+TMP
Sph+Bortezomib
NC
Sph
Sph+TMP
Sph+Bortezomib
140 kDa
POLG
NC
Sph
Sph+TMP
Sph+Bortezomib
140 kDa
POLG
NC
Sph
Sph+TMP
Sph+Bortezomib
NC
Sph
Sph+TMP
Sph+Bortezomib
50 kDa
40 kDa
35 kDa
43 kDa
β-actin
NC
Sph
Sph+TMP
Sph+Bortezomib
43 kDa
β-actin

Fig 4 H

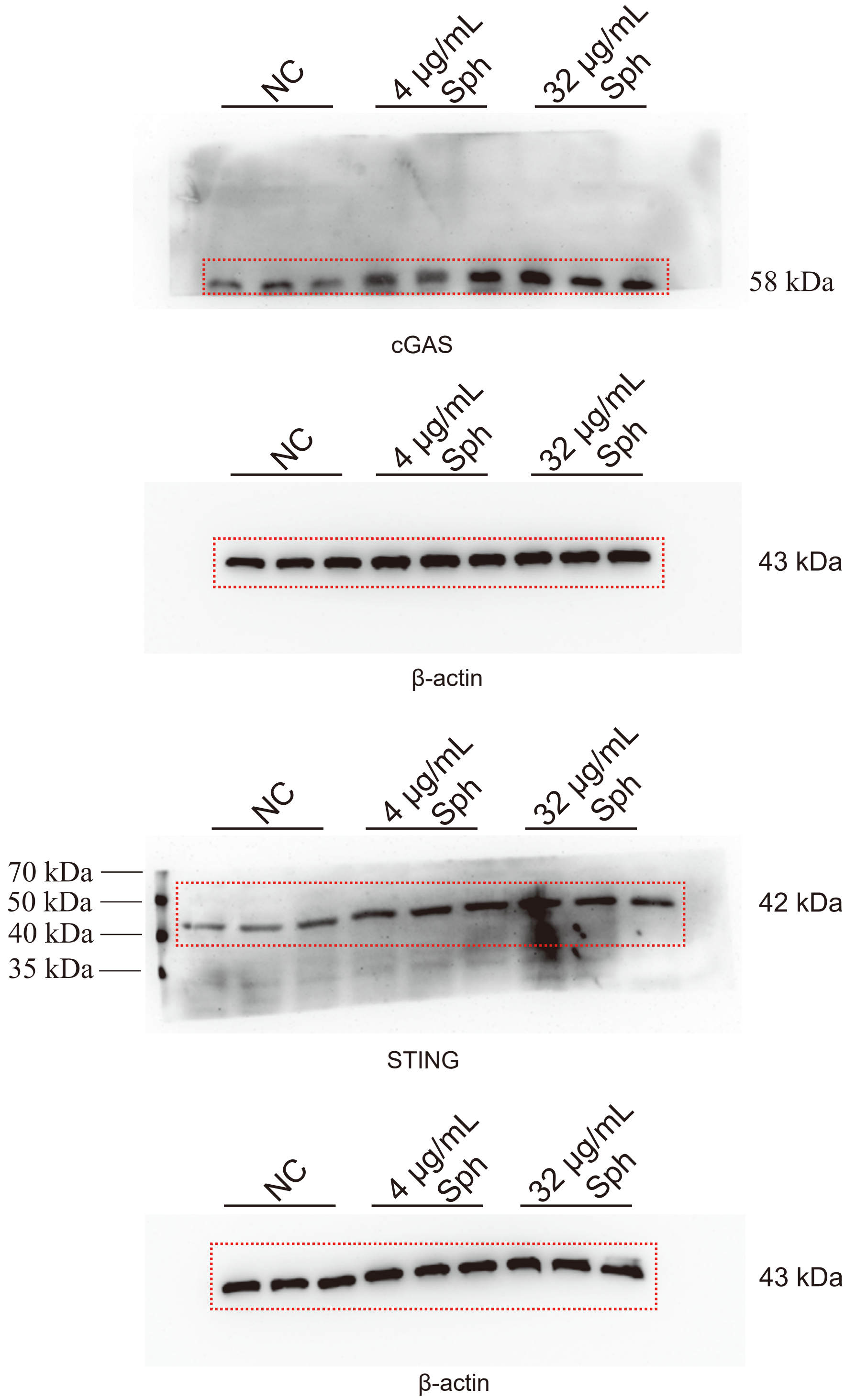
NC
4 μg/mL Sph
32 μg/mL Sph
58 kDa
cGAS
NC
4 μg/mL Sph
32 μg/mL Sph
43 kDa
β-actin
NC
4 μg/mL Sph
32 μg/mL Sph
70 kDa
50 kDa
40 kDa
35 kDa
42 kDa
STING
NC
4 μg/mL Sph
32 μg/mL Sph
43 kDa
β-actin

Fig 5 A

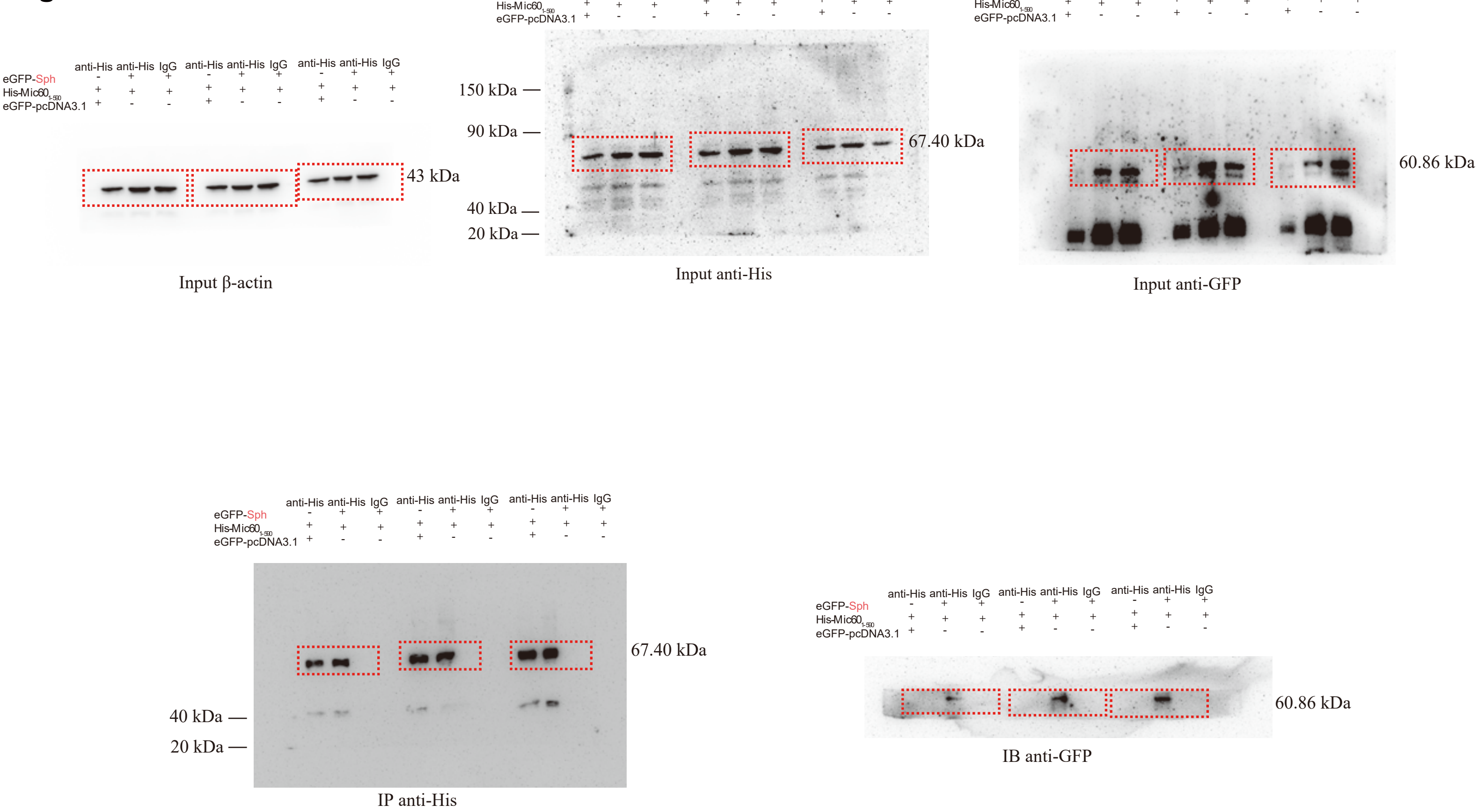
anti-His anti-His IgG
eGFP-Sph
His-Mic60 1-590
eGFP-pcDNA3.1
43 kDa
Input β-actin
150 kDa
90 kDa
40 kDa
20 kDa
67.40 kDa
Input anti-His
60.86 kDa
Input anti-GFP
IP anti-His
IB anti-GFP

Fig 5 B

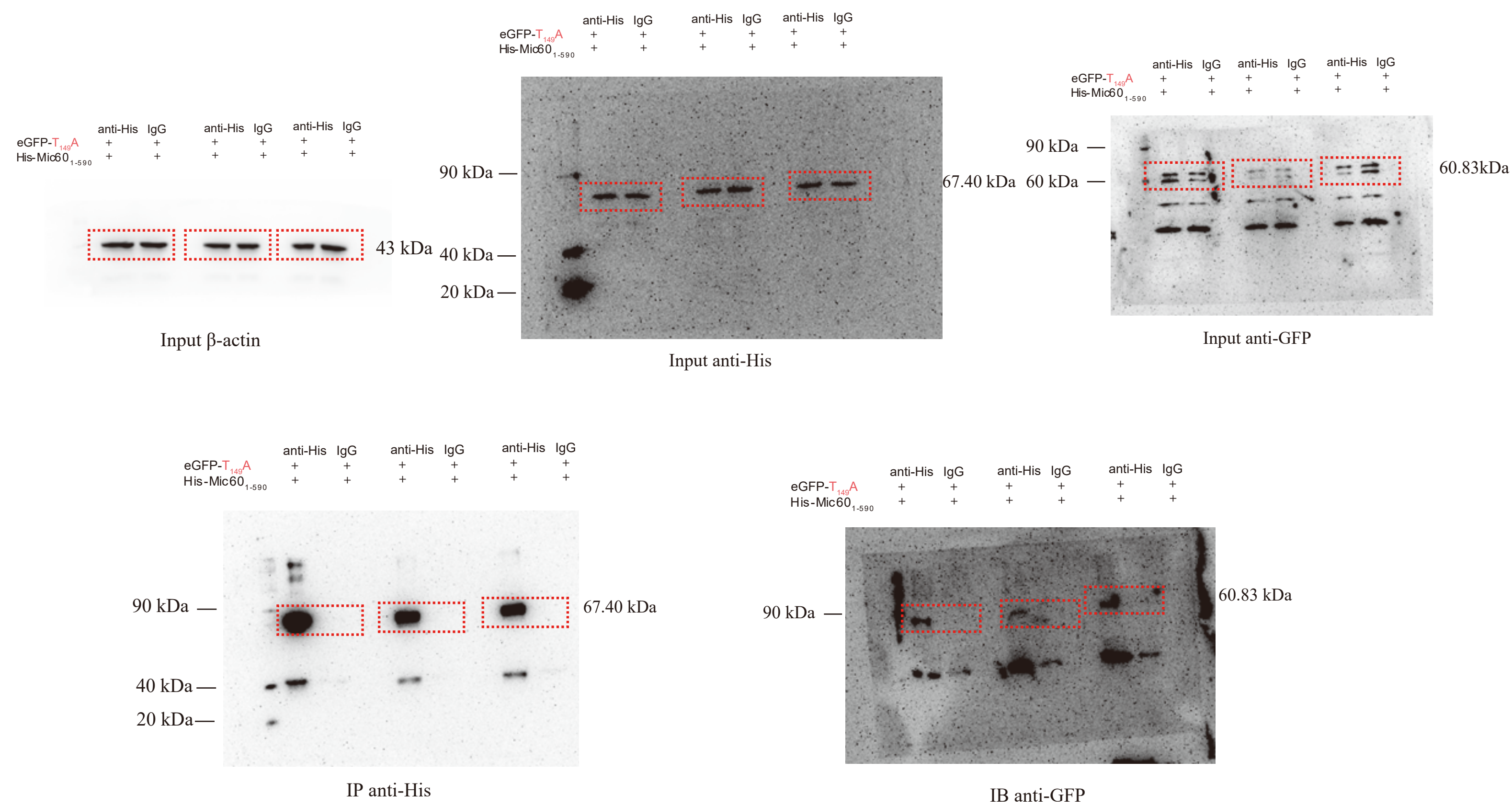
anti-His IgG
eGFP-T149A
His-Mic60 1-590
43 kDa
Input β-actin
90 kDa
40 kDa
20 kDa
67.40 kDa
Input anti-His
60 kDa
60.83kDa
Input anti-GFP
IP anti-His
60.83 kDa
IB anti-GFP

Fig 5 C

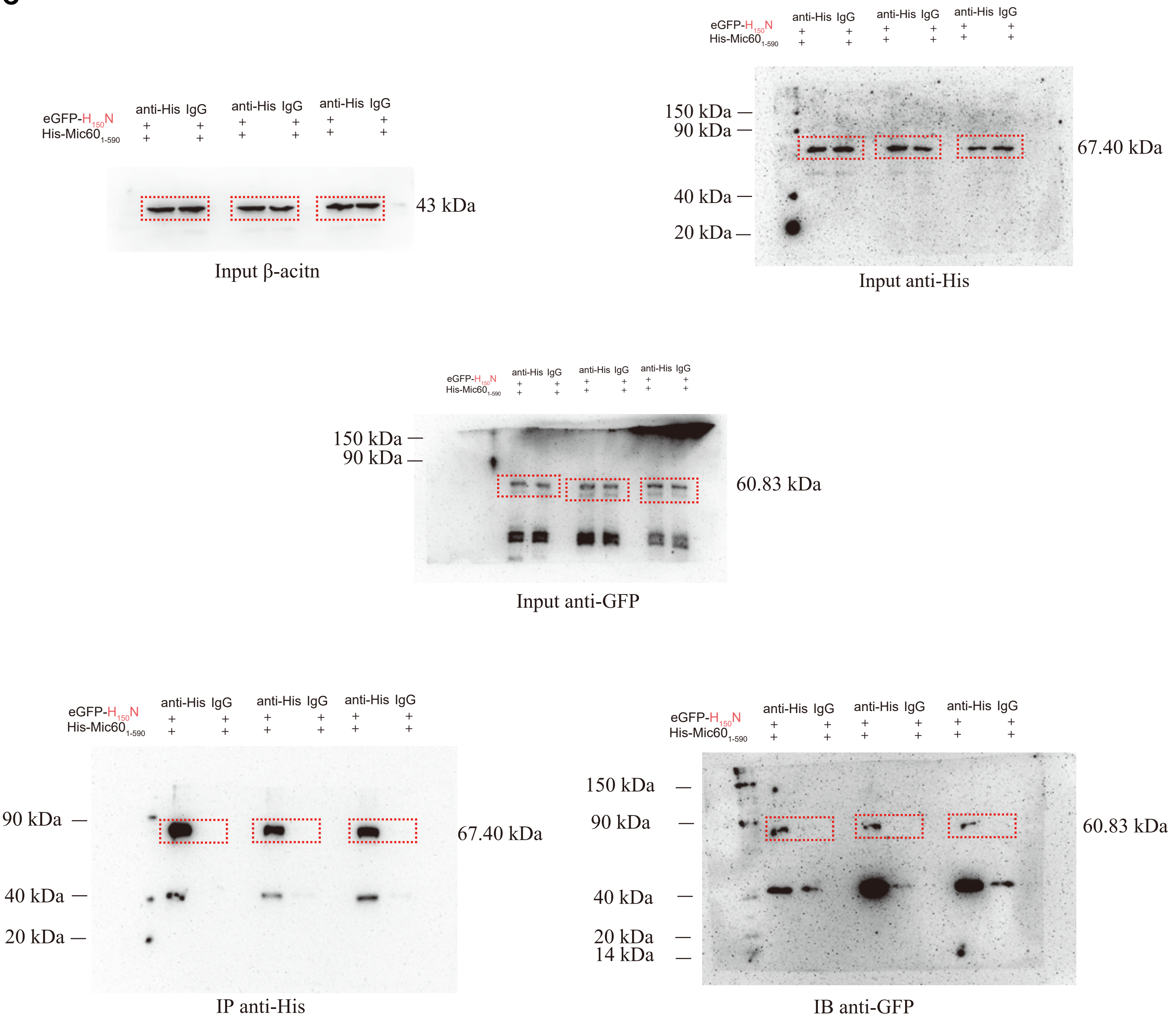
eGFP-H150N
His-Mic60 1-590
anti-His IgG
43 kDa
Input β-acitn
150 kDa
90 kDa
40 kDa
20 kDa
67.40 kDa
Input anti-His
60.83 kDa
Input anti-GFP
IP anti-His
14 kDa
IB anti-GFP

Fig 5 E

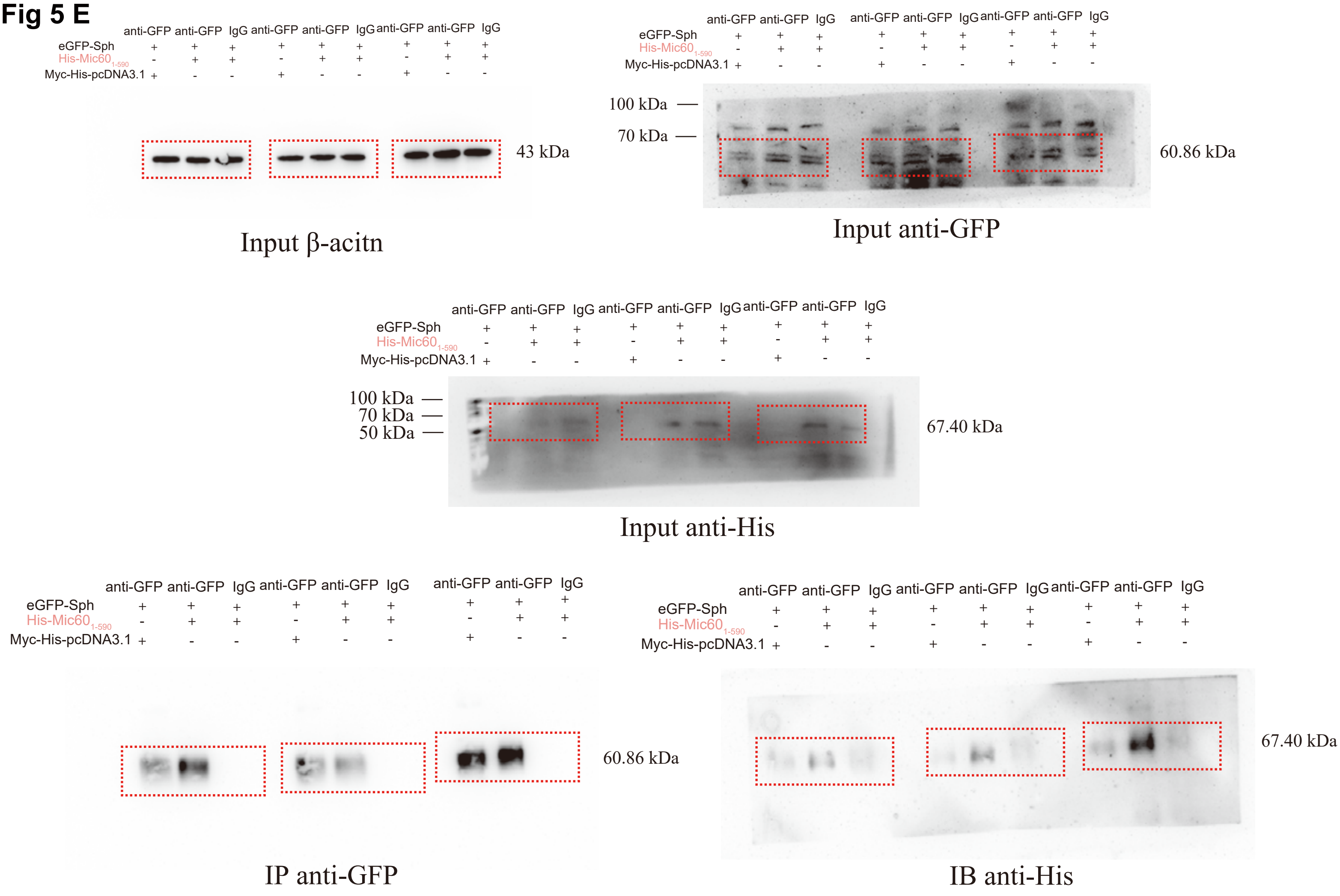

anti-GFP anti-GFP IgG anti-GFP anti-GFP IgG anti-GFP anti-GFP IgG
eGFP-Sph
His-Mic60$_{1\text{-}590}$
Myc-His-pcDNA3.1
43 kDa
Input β-acitn
100 kDa
70 kDa
60.86 kDa
Input anti-GFP
100 kDa
70 kDa
50 kDa
67.40 kDa
Input anti-His
60.86 kDa
IP anti-GFP
67.40 kDa
IB anti-His

Fig 5 F

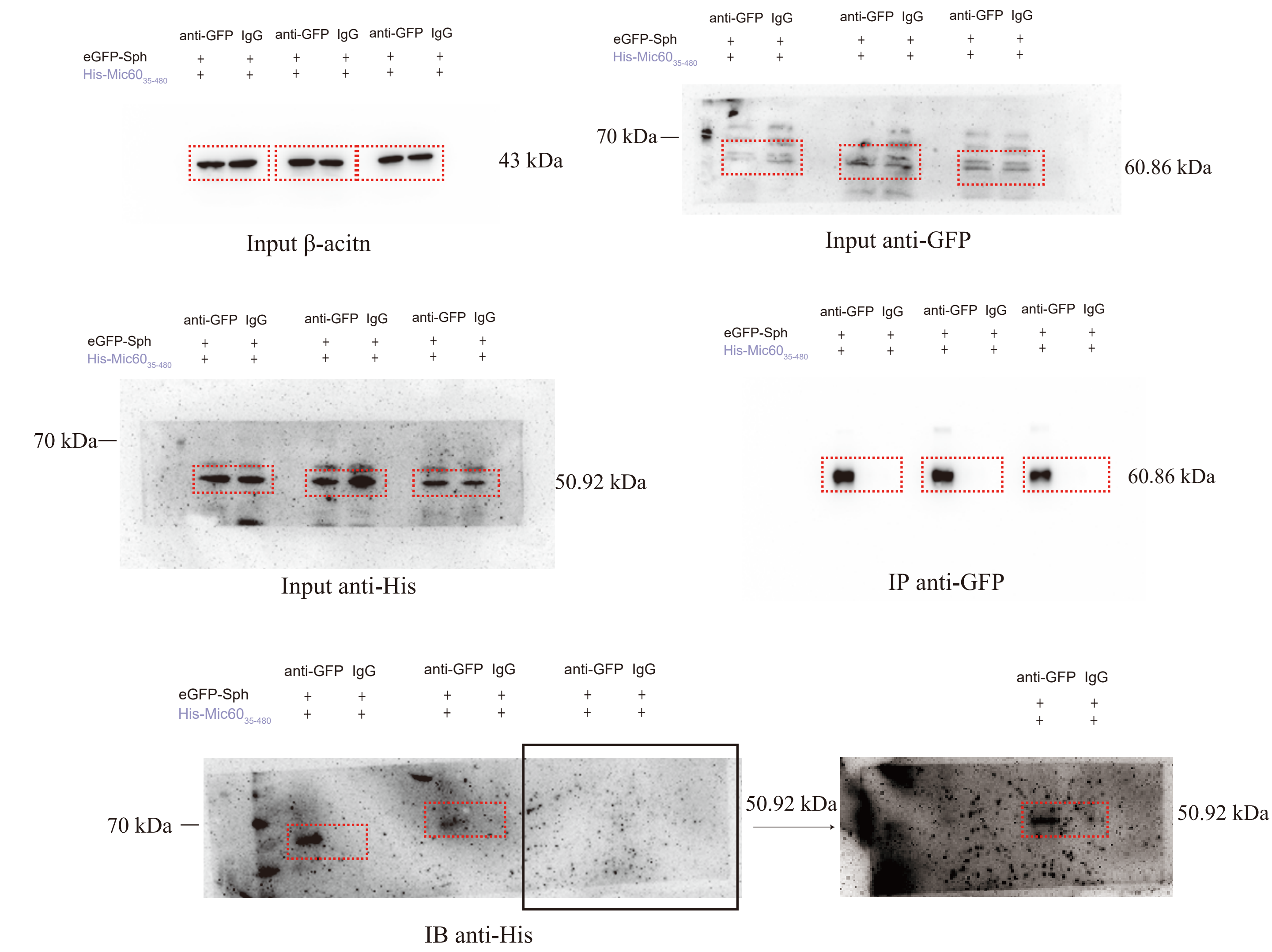

anti-GFP IgG anti-GFP IgG anti-GFP IgG
eGFP-Sph
His-Mic60$_{35\text{-}480}$
43 kDa
Input β-acitn
70 kDa
60.86 kDa
Input anti-GFP
70 kDa
50.92 kDa
Input anti-His
60.86 kDa
IP anti-GFP
70 kDa
50.92 kDa
50.92 kDa
IB anti-His

Fig 5 G

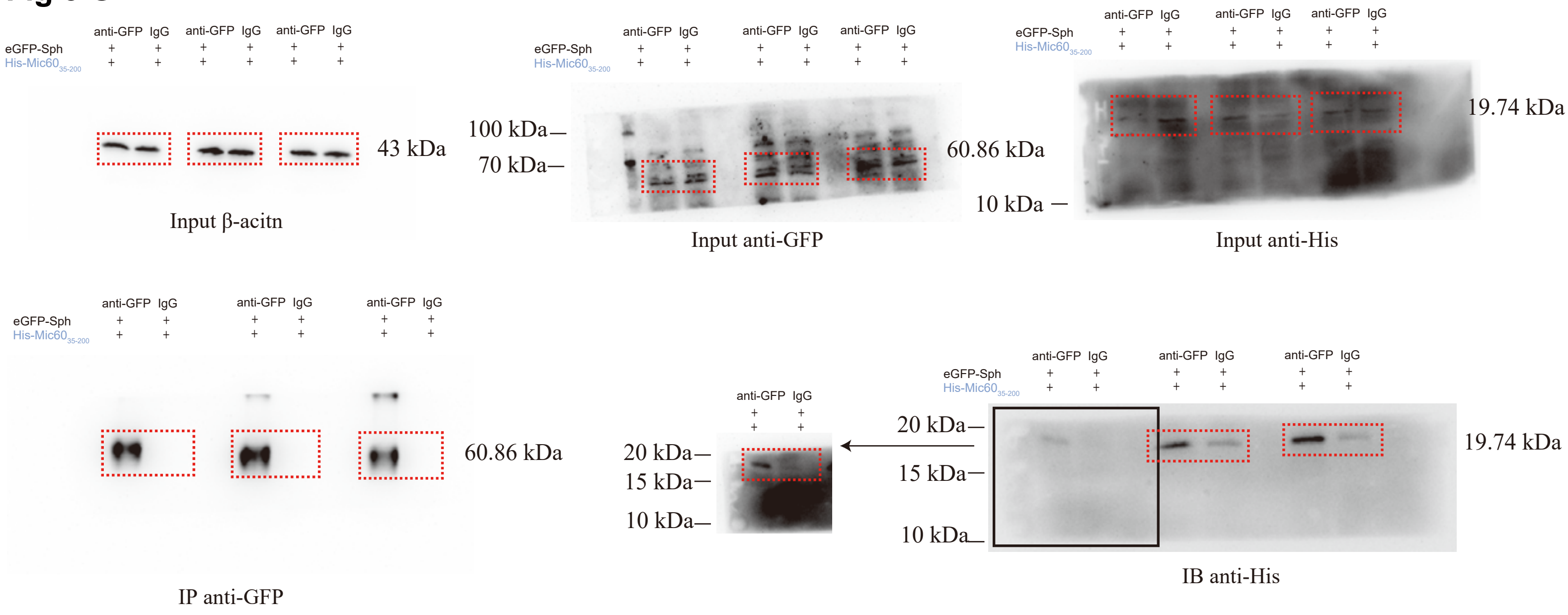

Fig 5 H

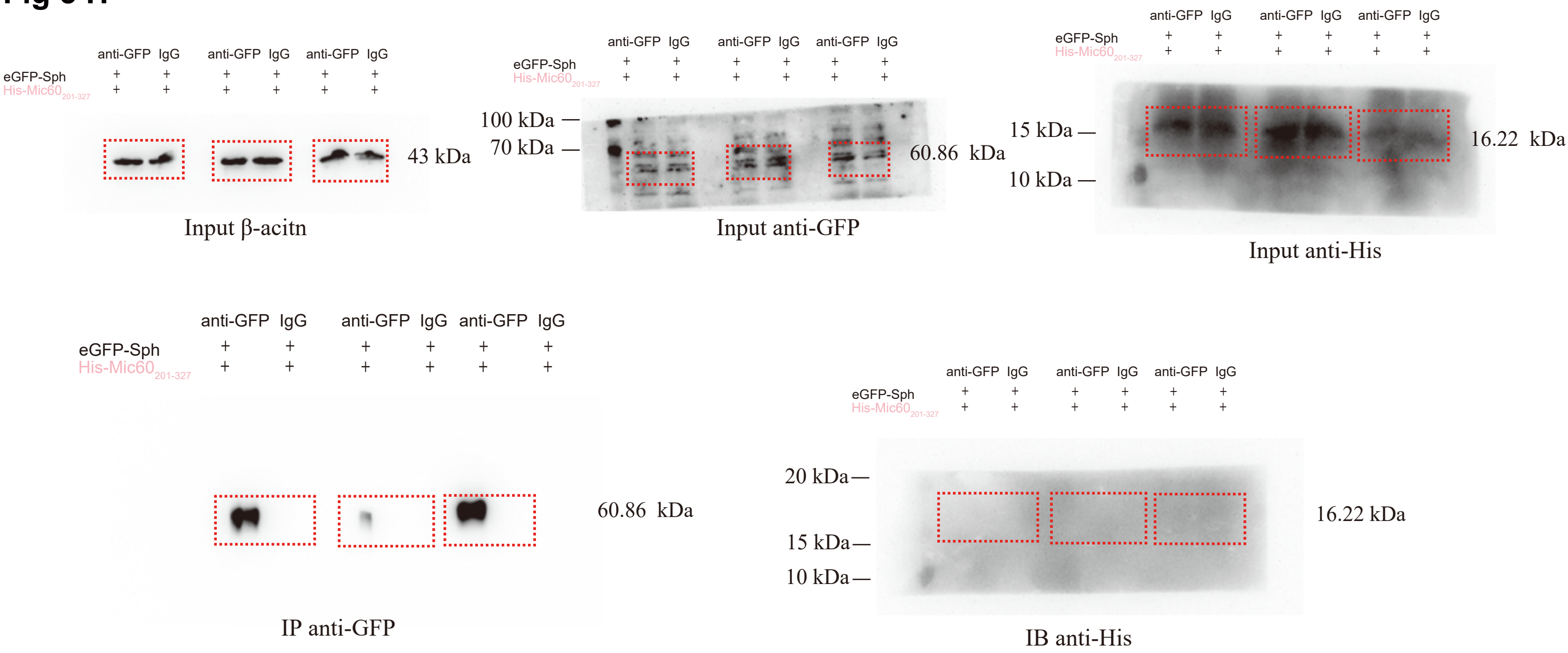

Fig 5 I

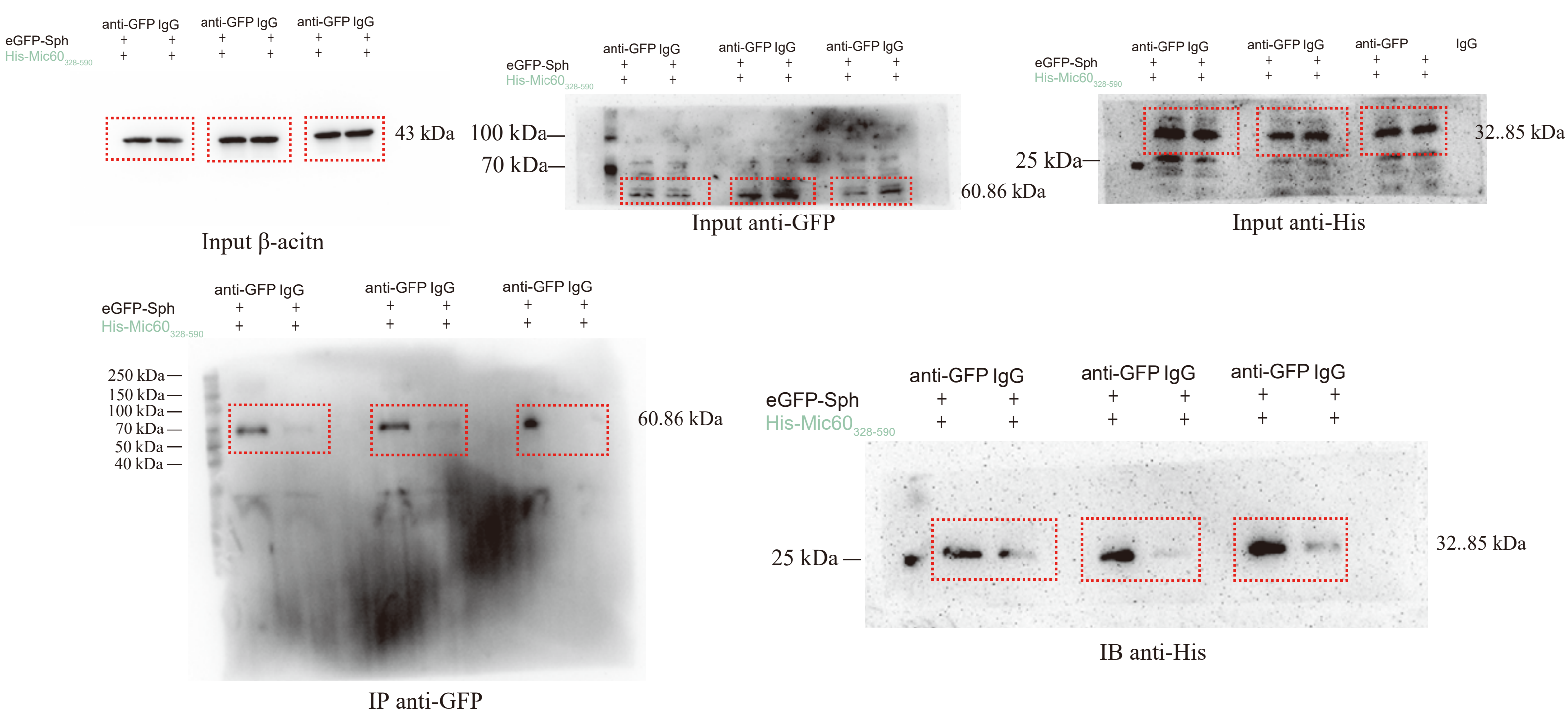

Fig 5 K

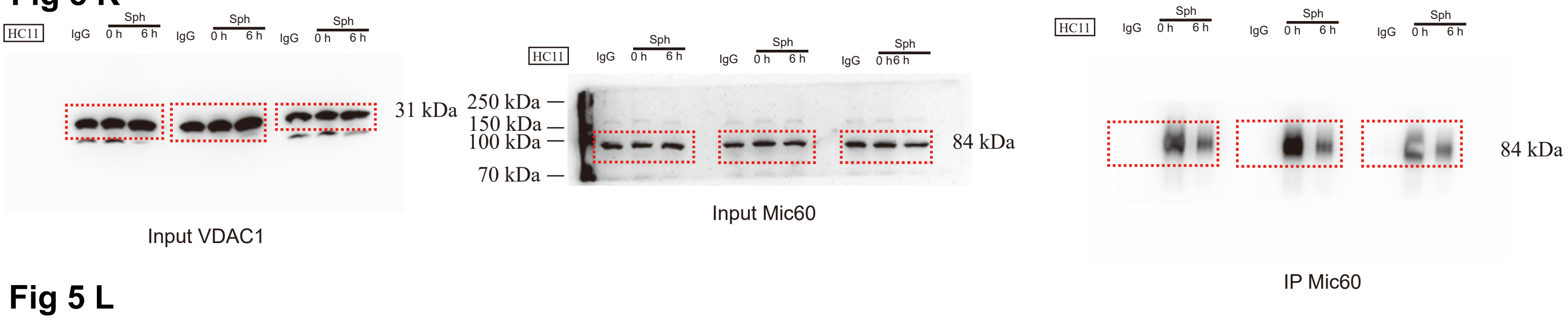

Fig 5 L

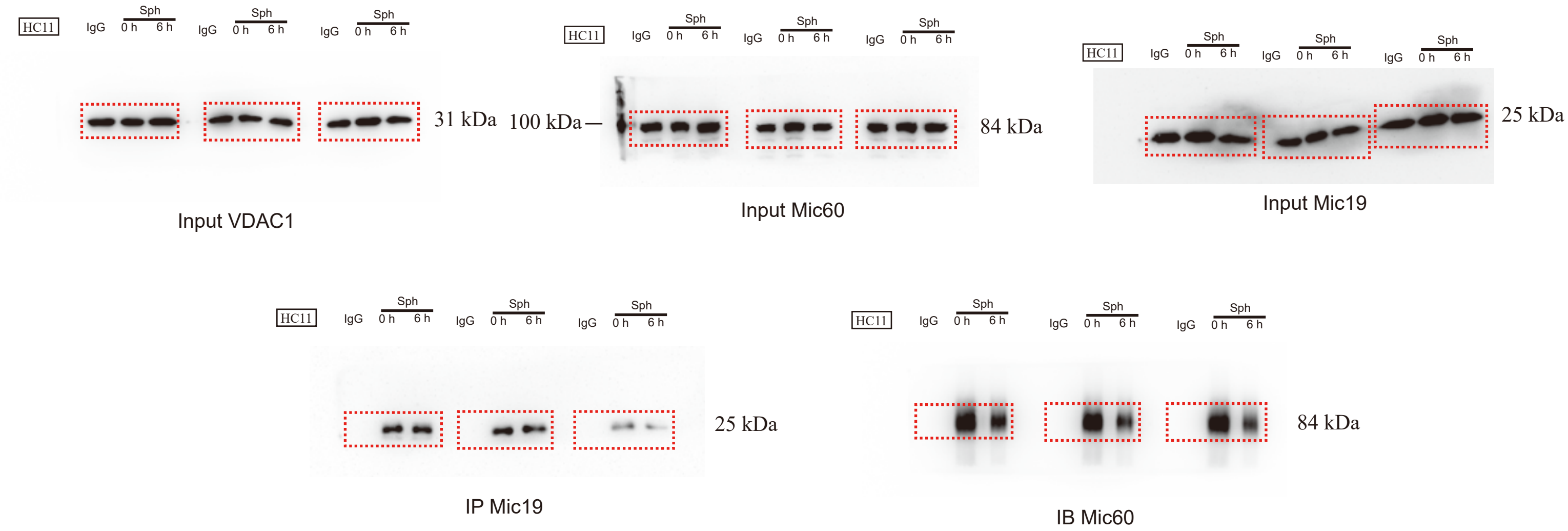

Fig 5 M

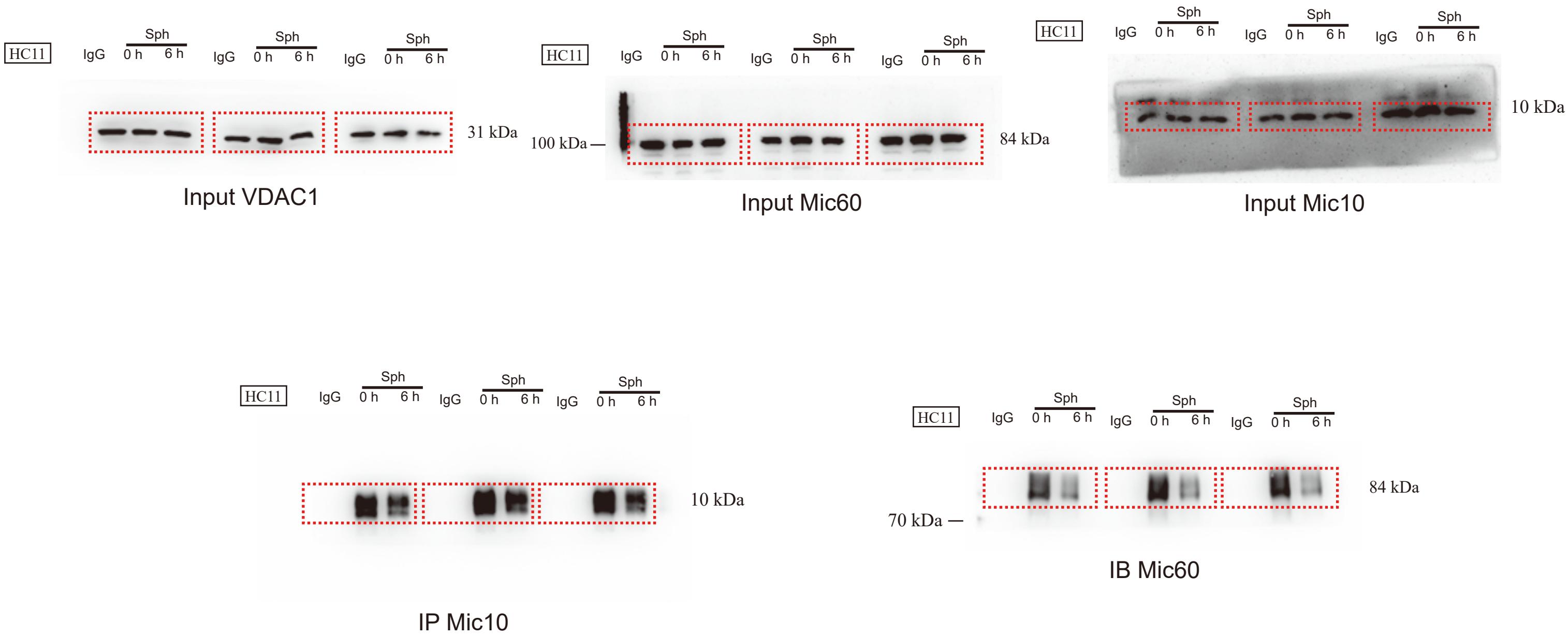

Fig 5 N

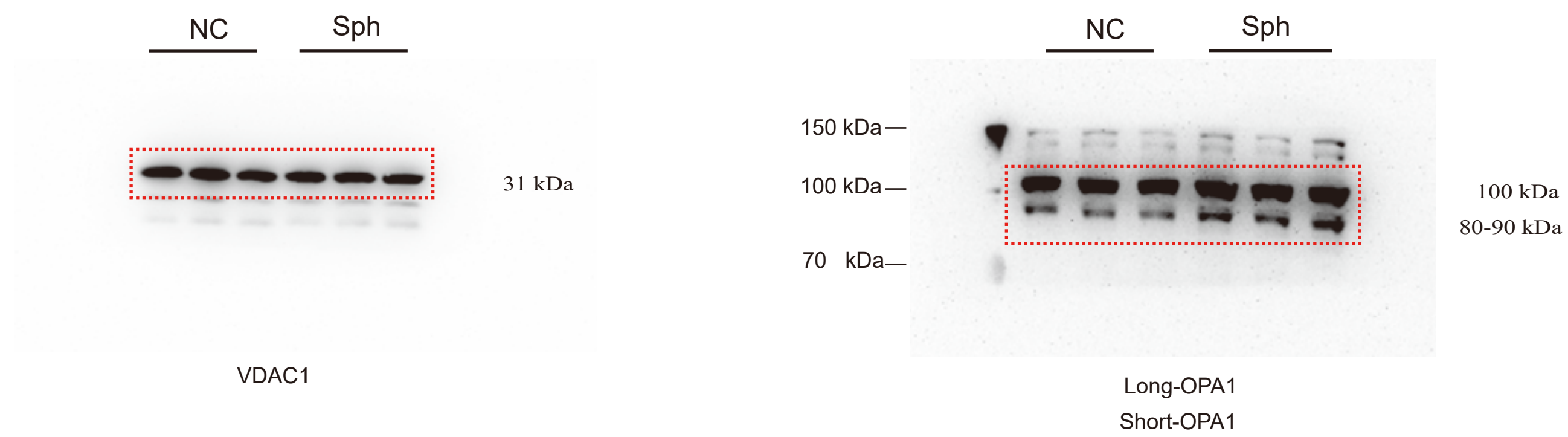

Fig 6 A

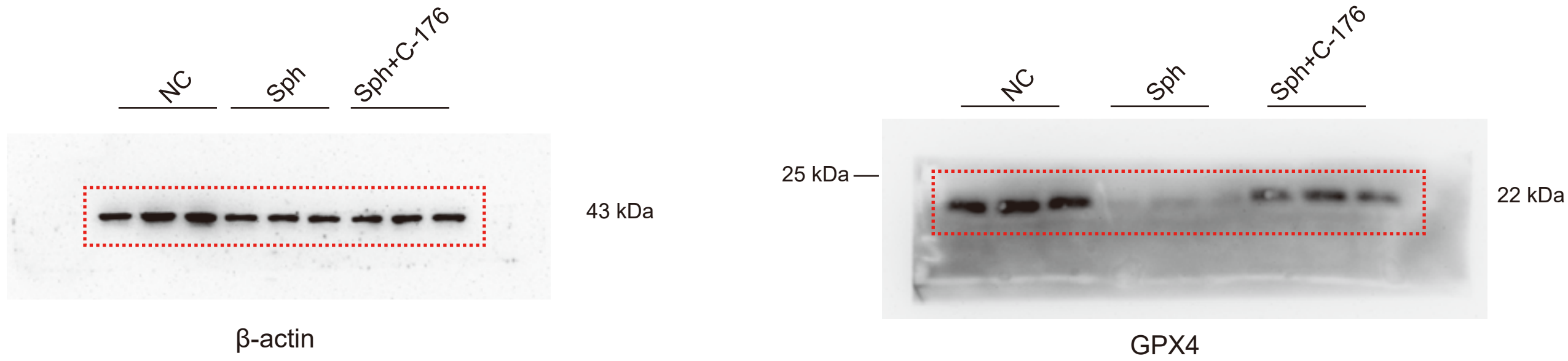

Fig 6 B

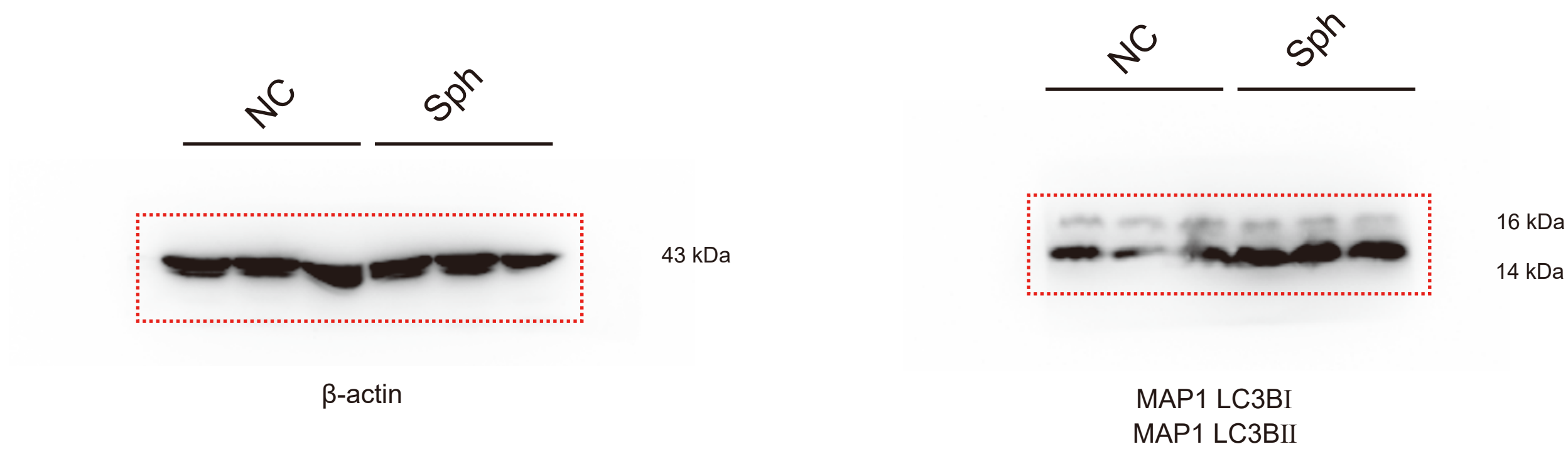

Fig 6 C

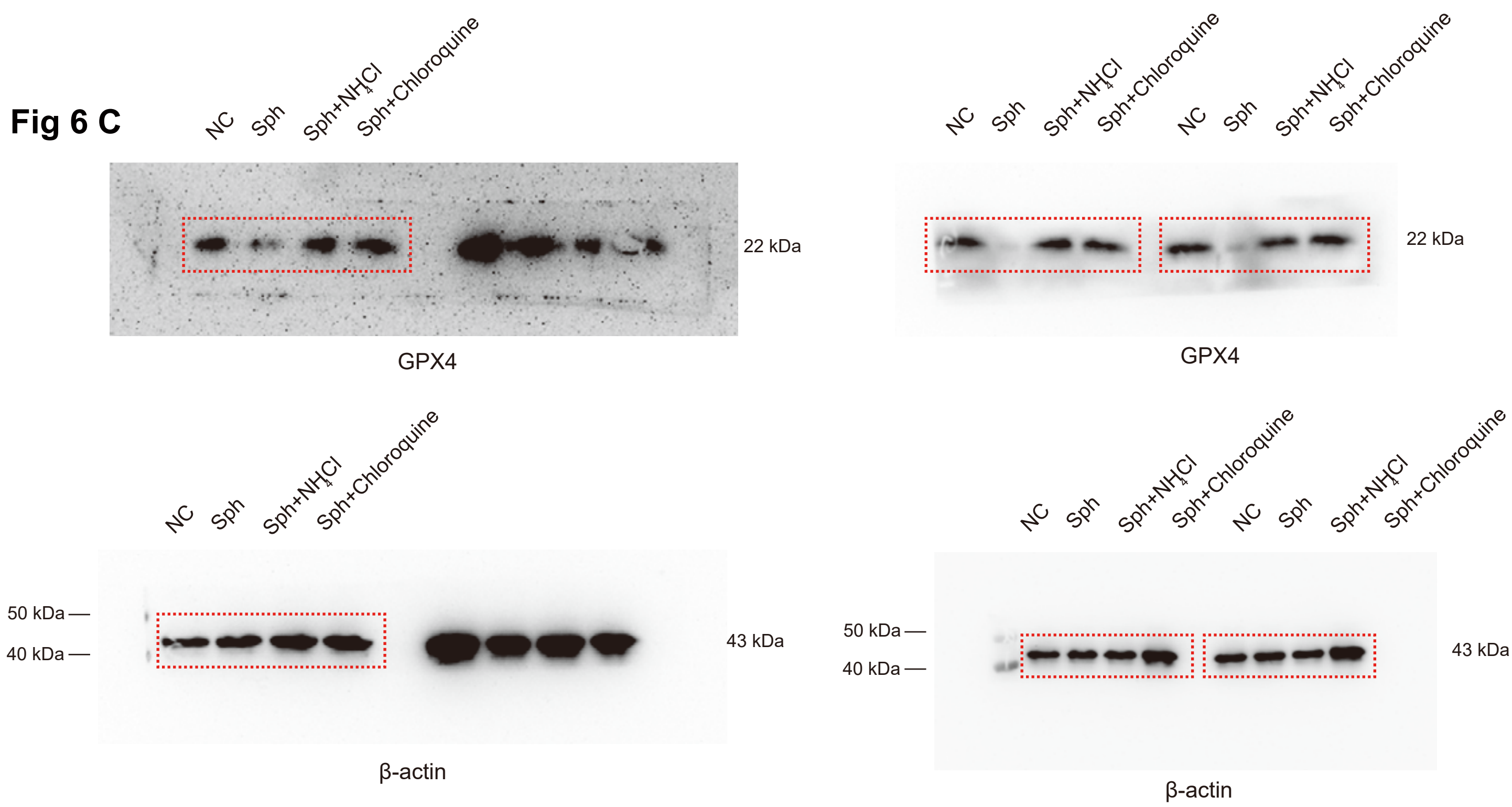

Fig 6 D

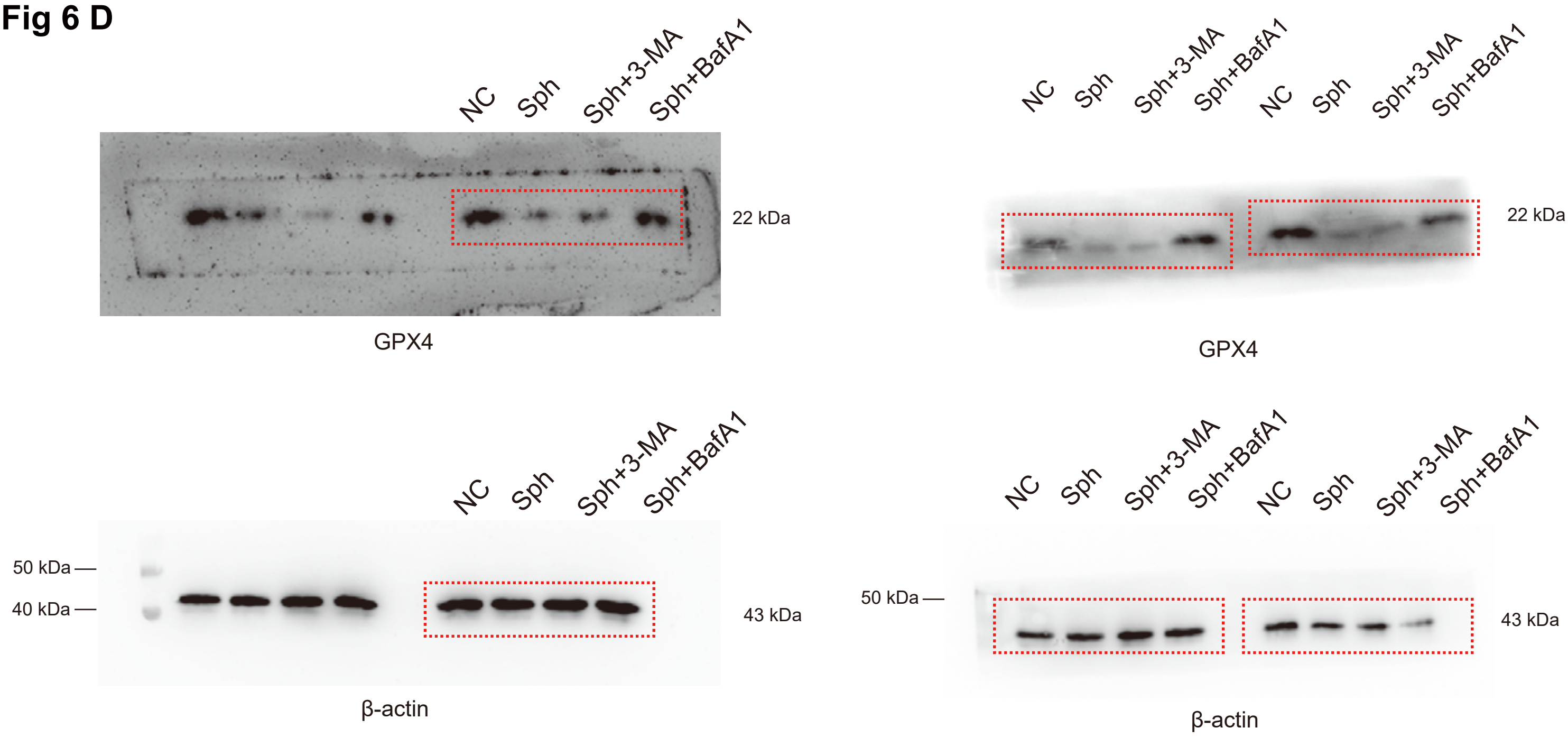

Fig 6 H

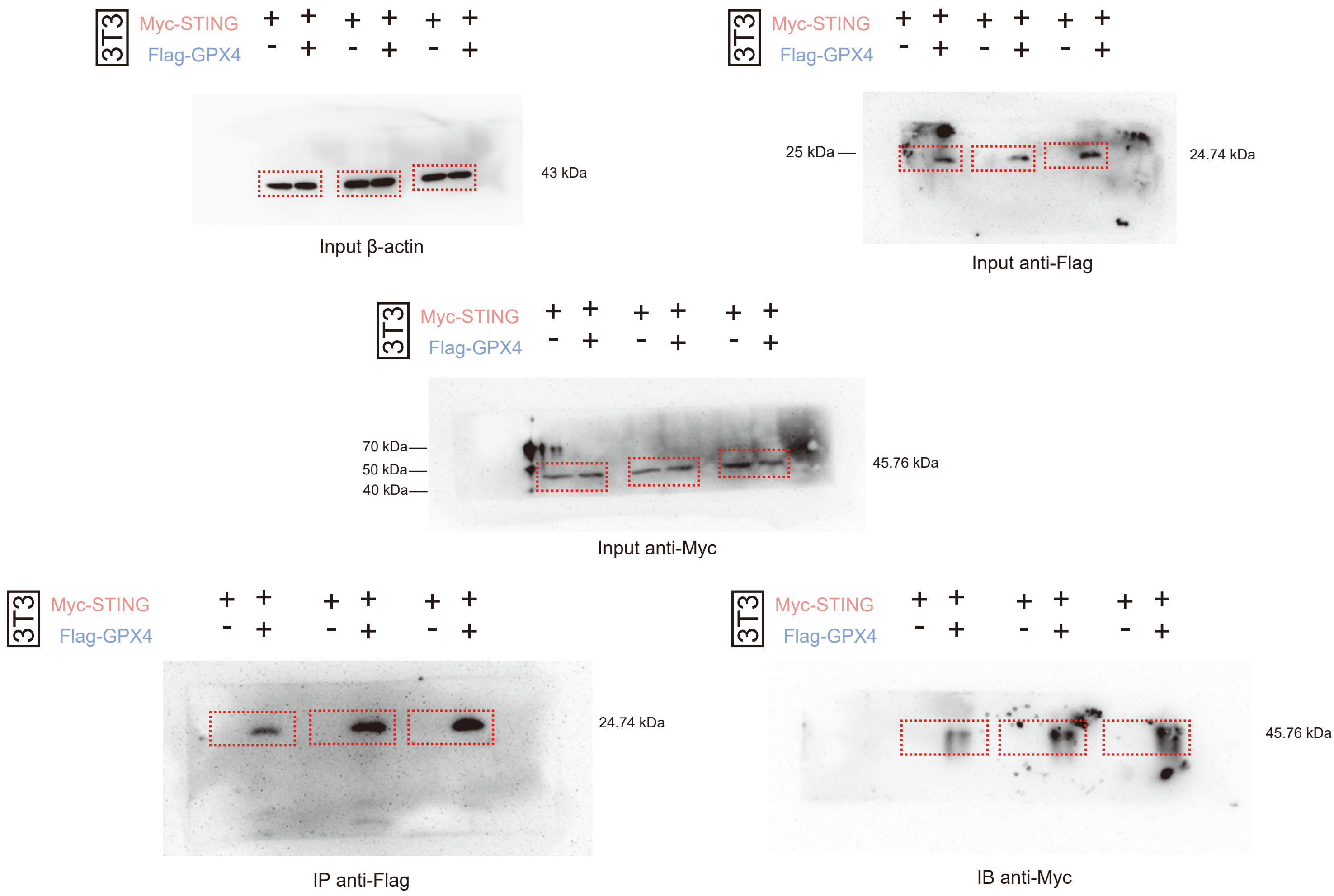
3T3
Myc-STING + + + + + +
Flag-GPX4 - + - + - +
43 kDa
Input β-actin
25 kDa
24.74 kDa
Input anti-Flag
70 kDa
50 kDa
40 kDa
45.76 kDa
Input anti-Myc
24.74 kDa
IP anti-Flag
45.76 kDa
IB anti-Myc

Fig 6 I

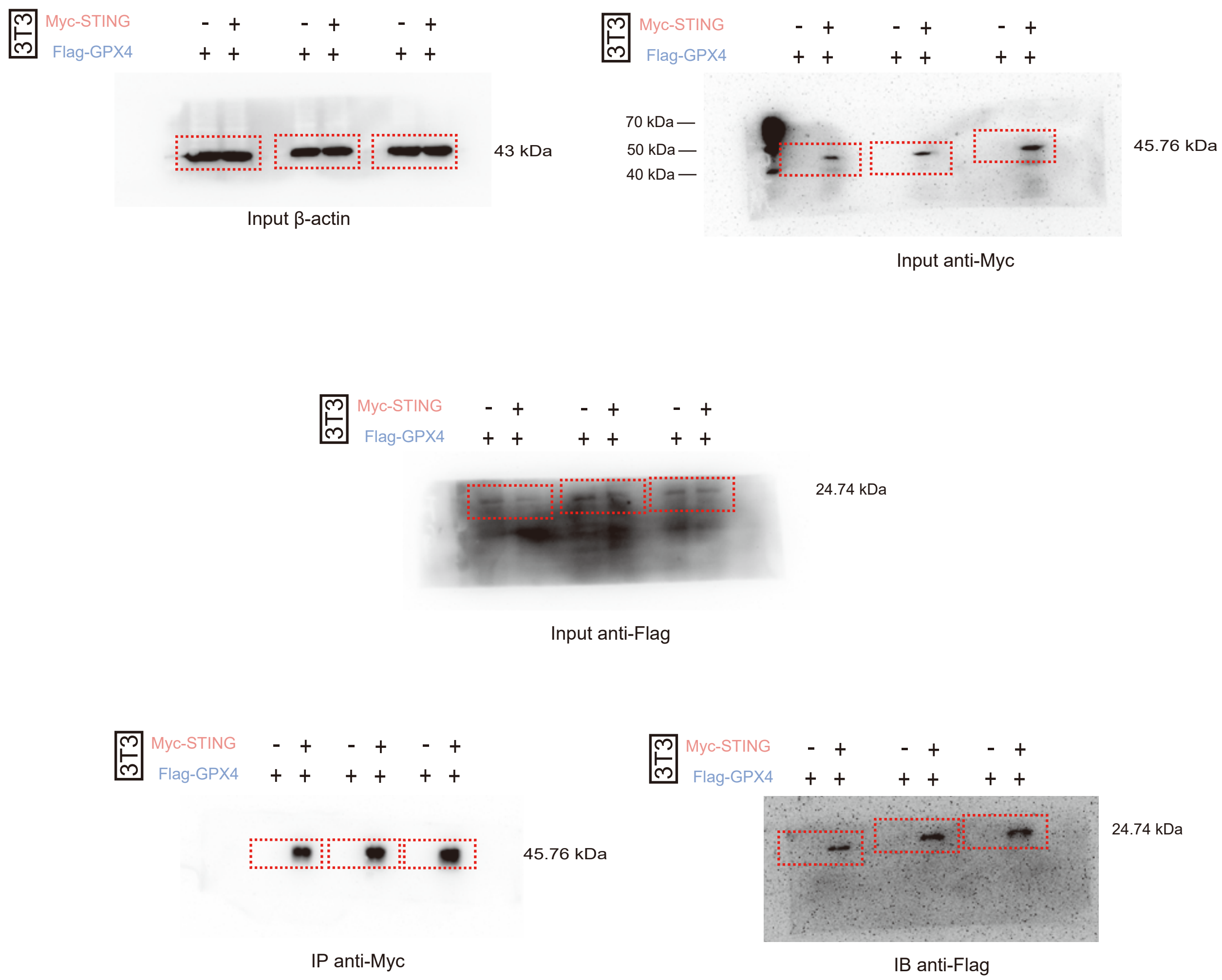
3T3
Myc-STING - + - + - +
Flag-GPX4 + + + + + +
43 kDa
Input β-actin
70 kDa
50 kDa
40 kDa
45.76 kDa
Input anti-Myc
24.74 kDa
Input anti-Flag
45.76 kDa
IP anti-Myc
24.74 kDa
IB anti-Flag

Fig s1 A

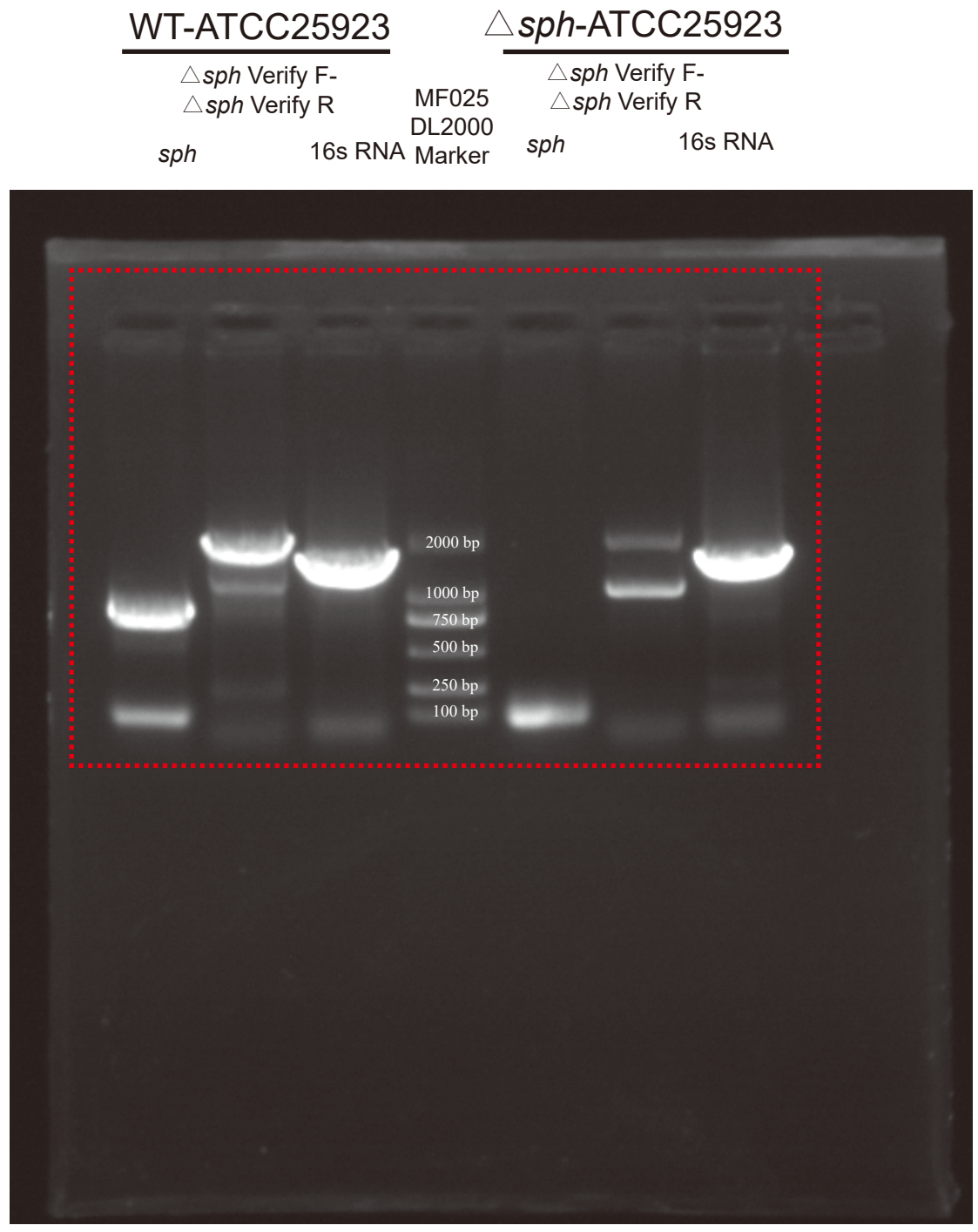

Fig s1 B

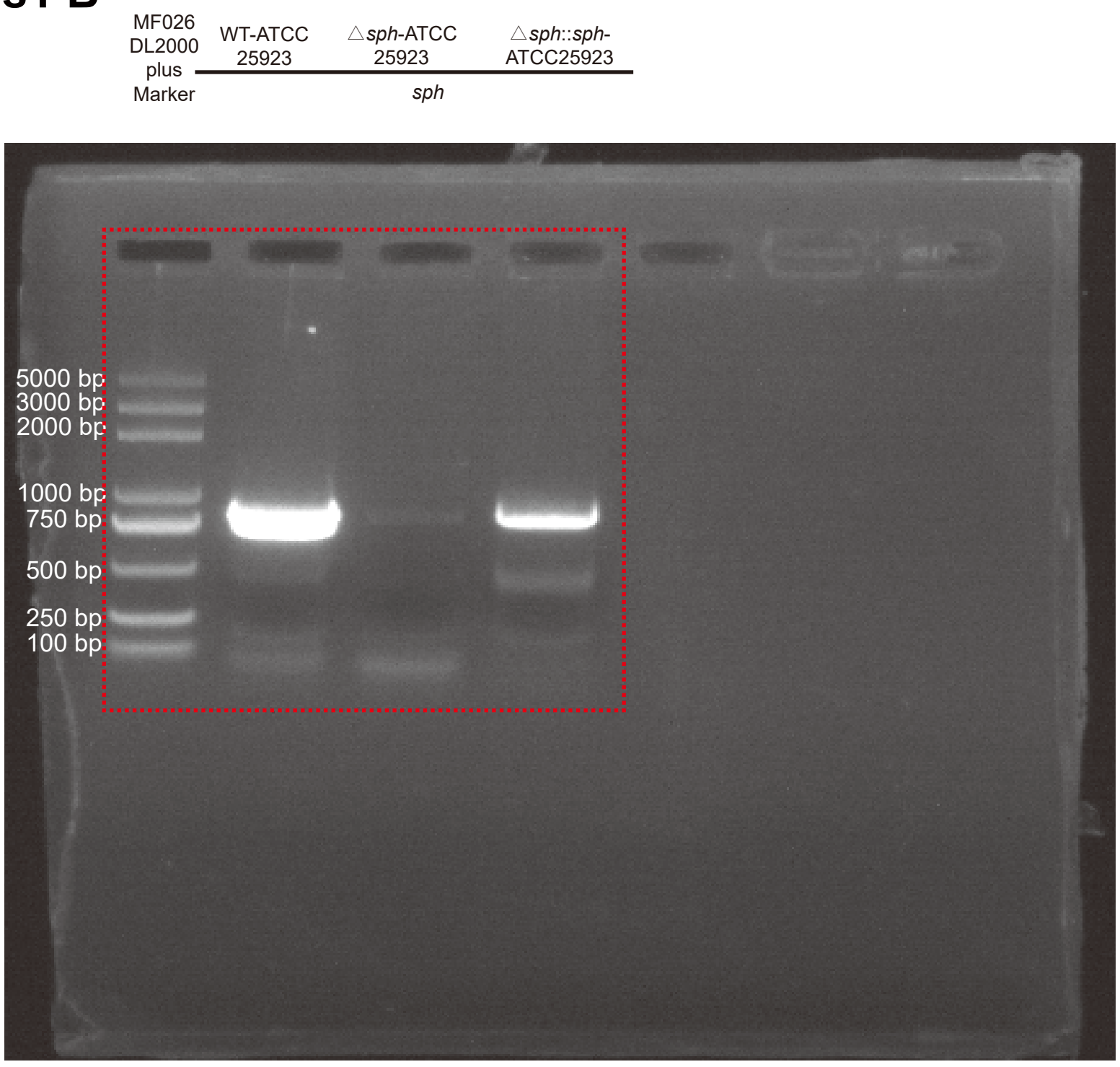

Fig s2 A

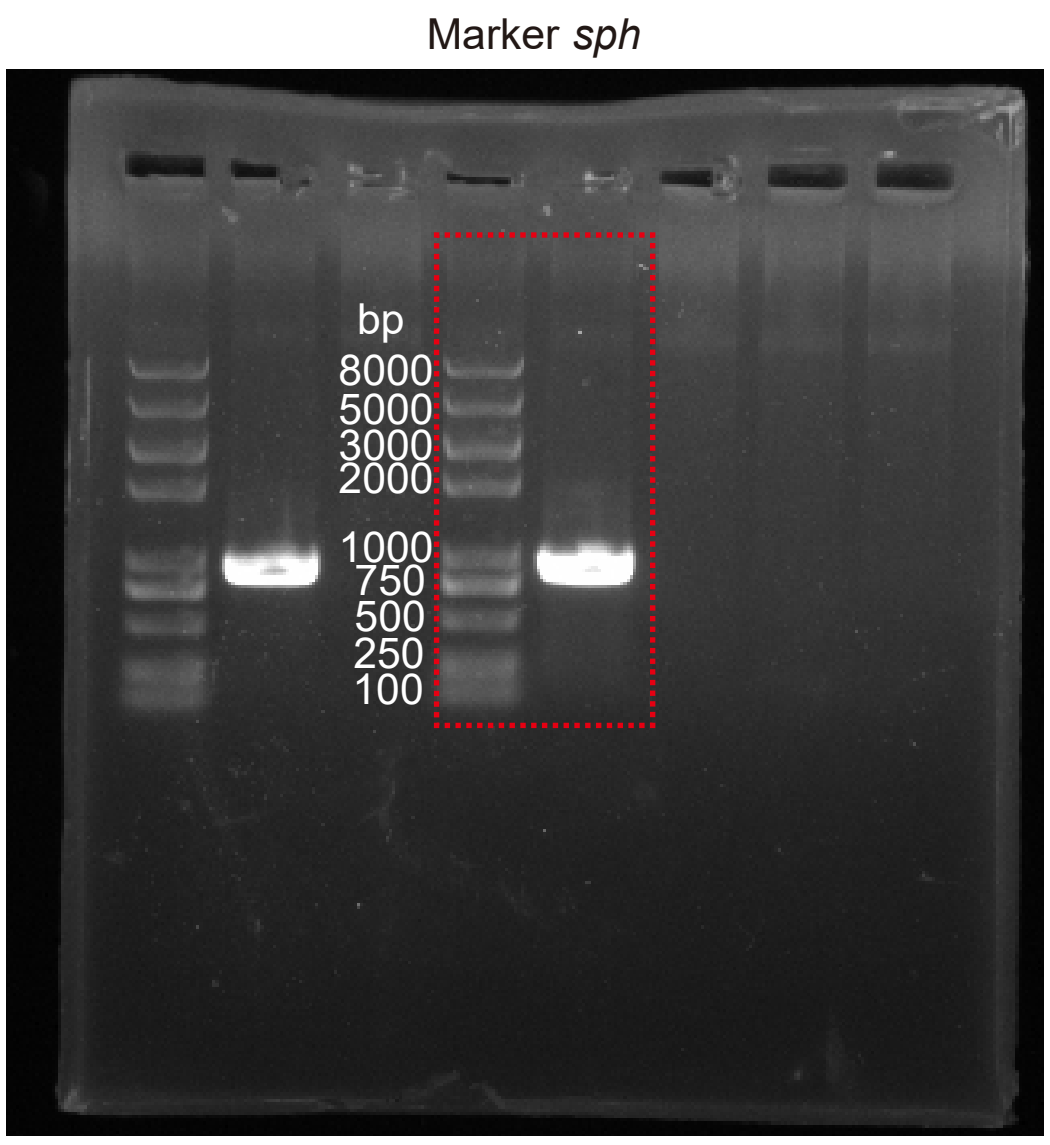

Fig s2 B

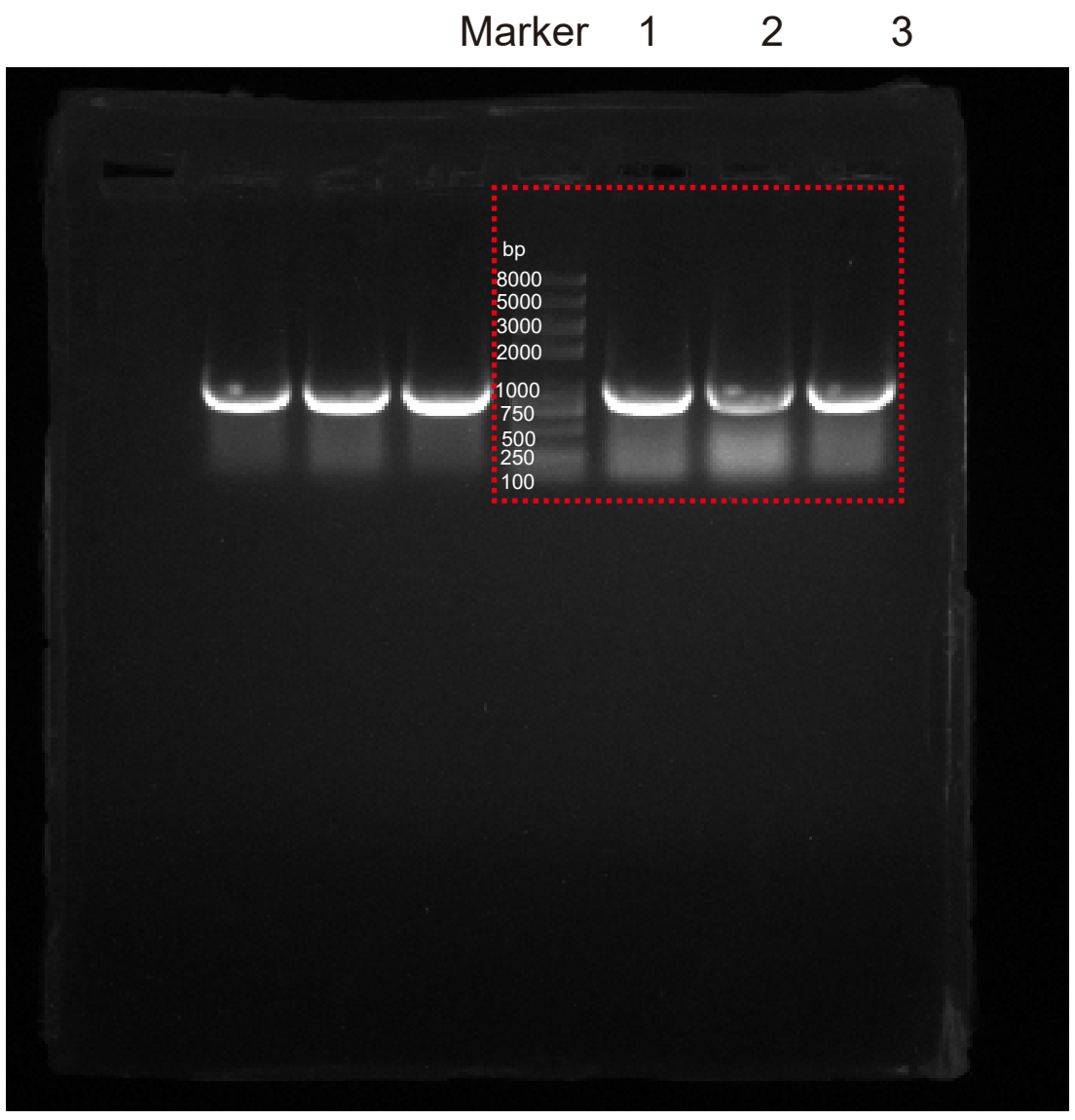

Fig s2 C

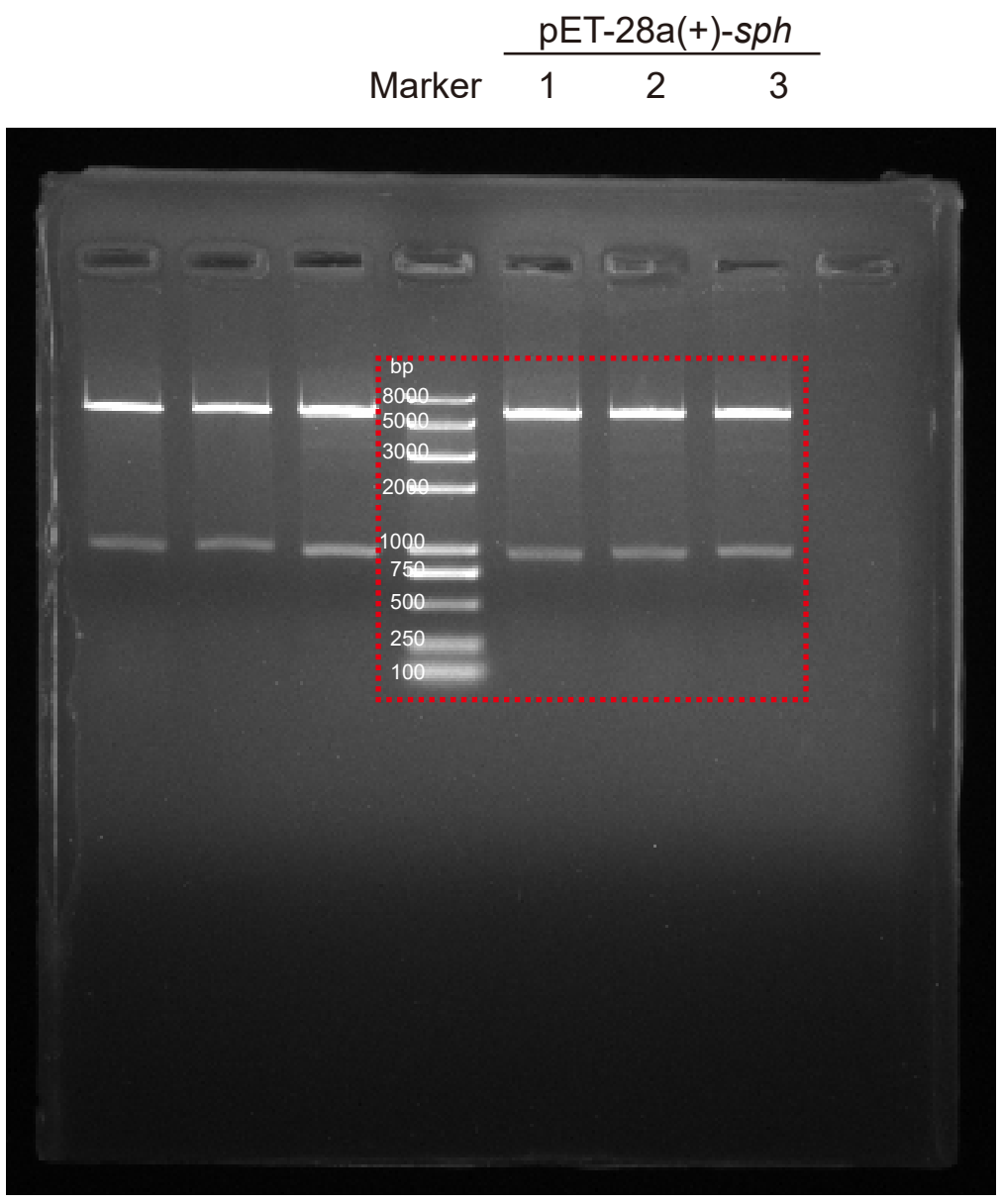

Fig s2 D

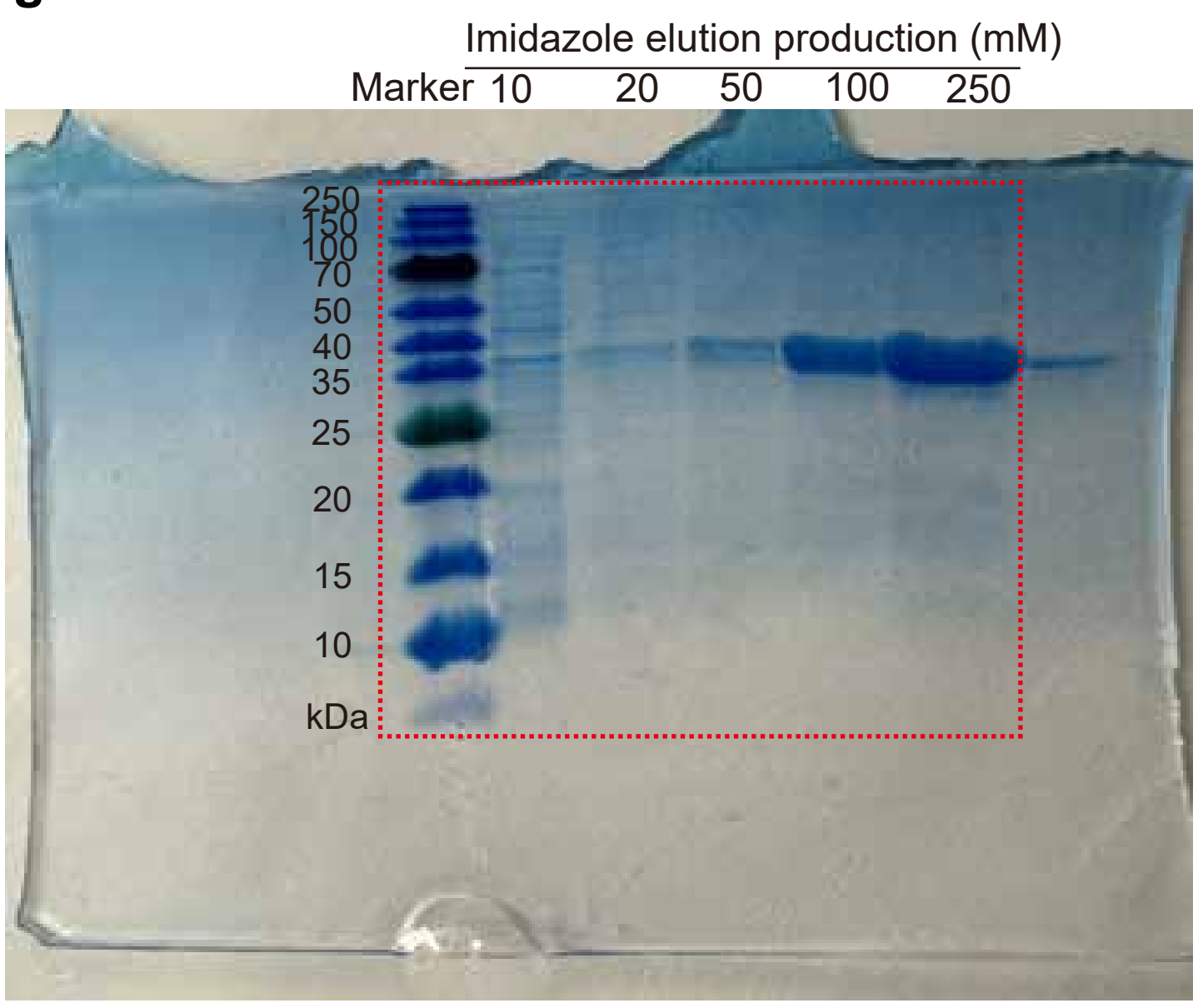

Fig s3 A

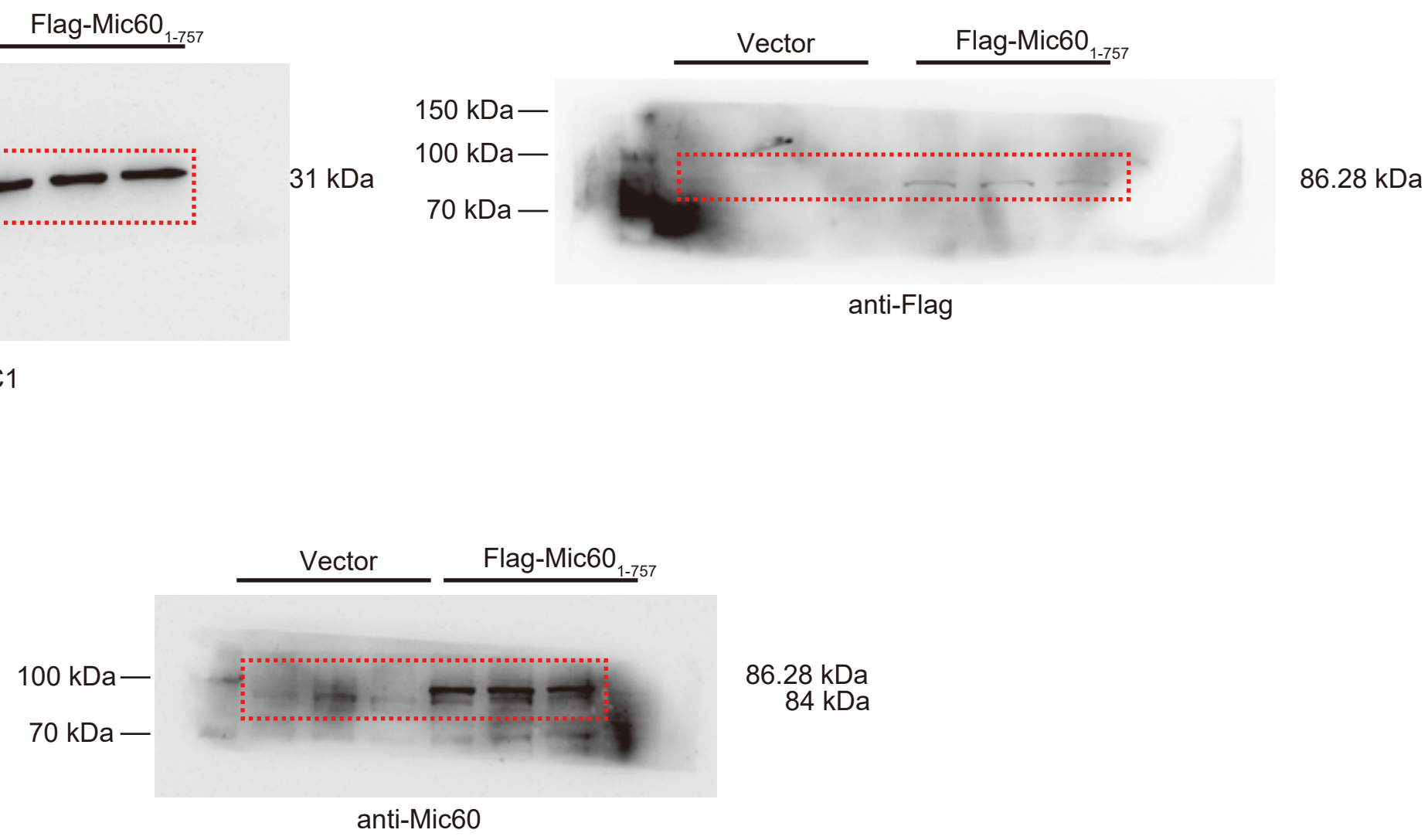

Fig s3 B

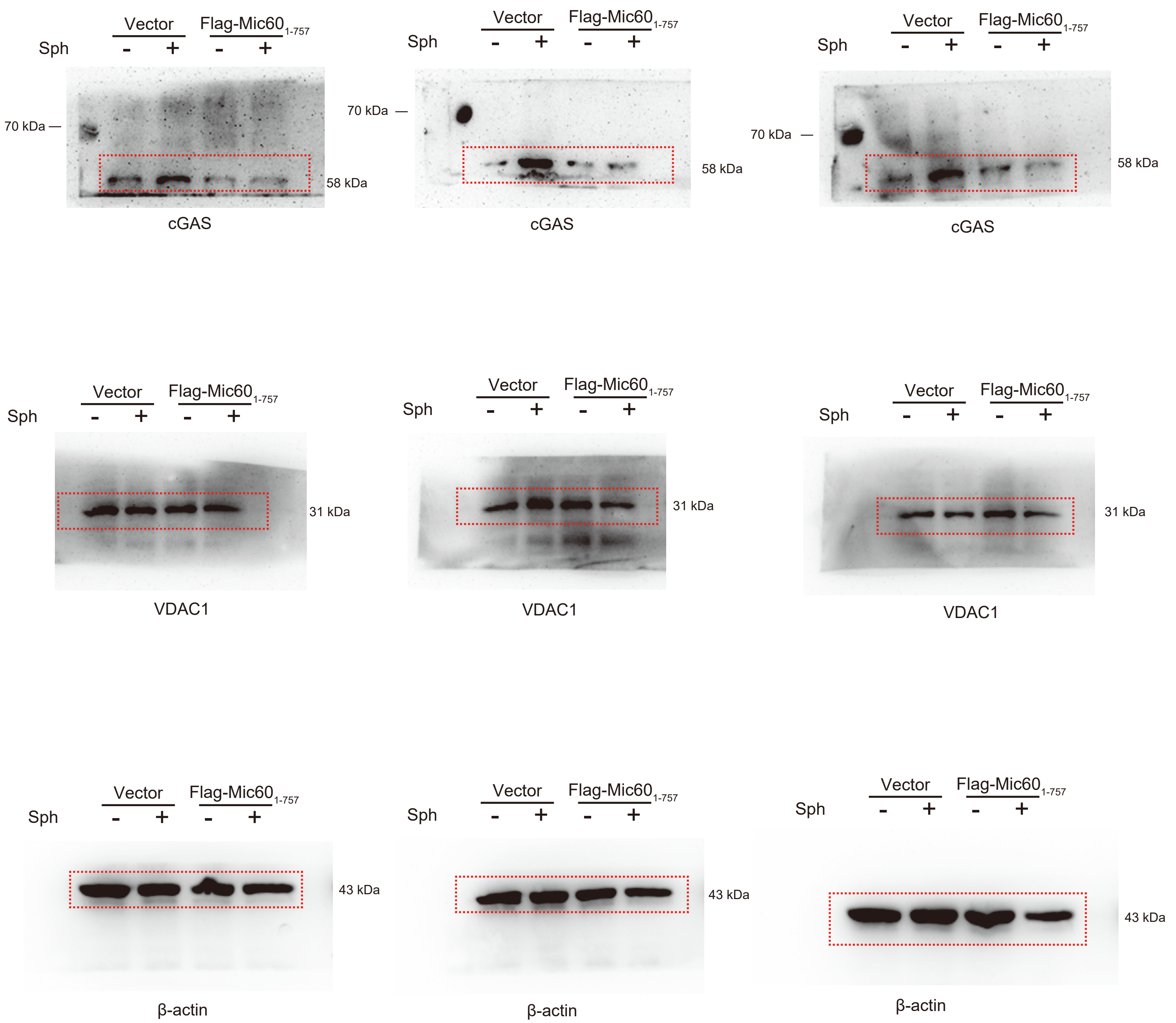

Fig s4 B

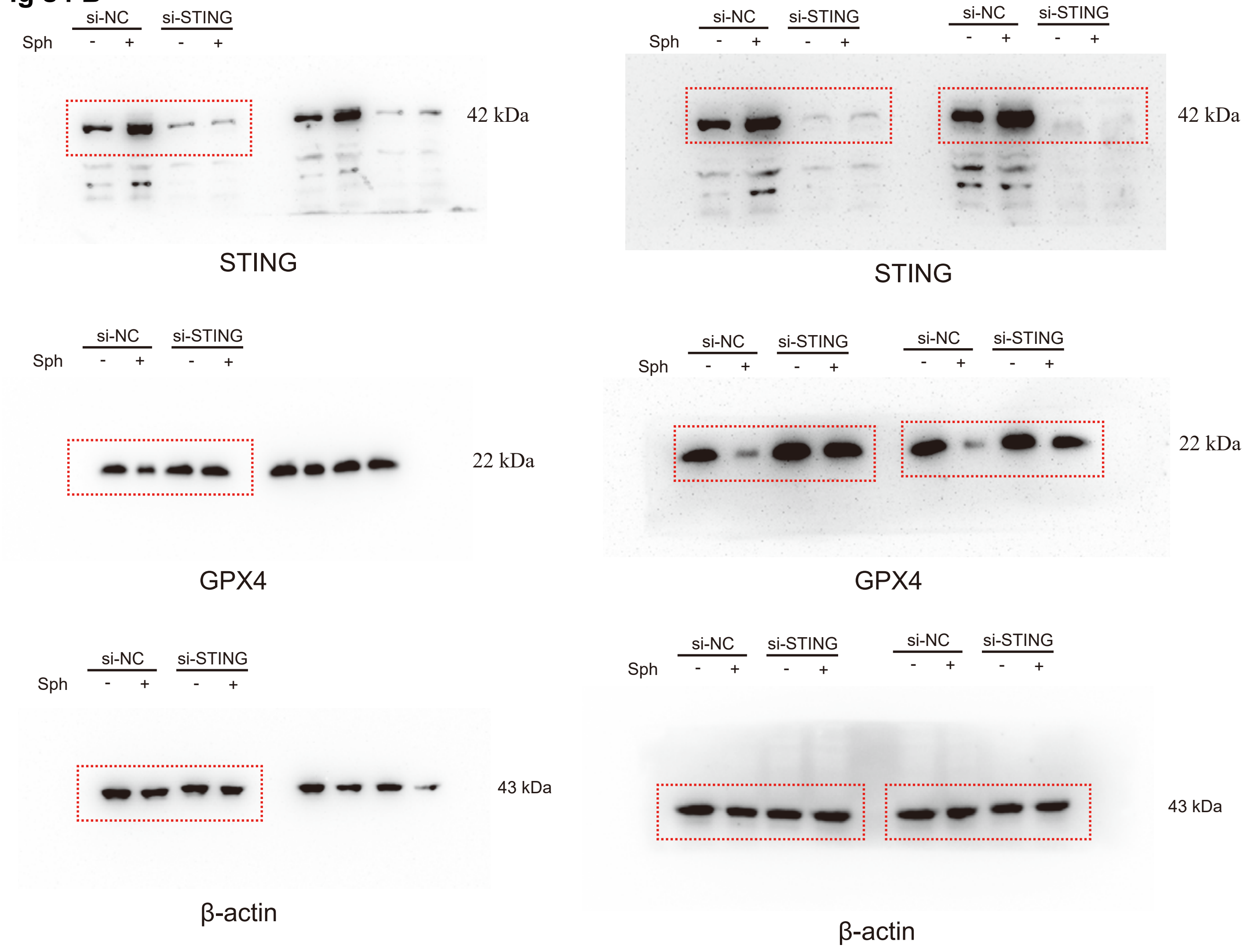

Fig s5 B

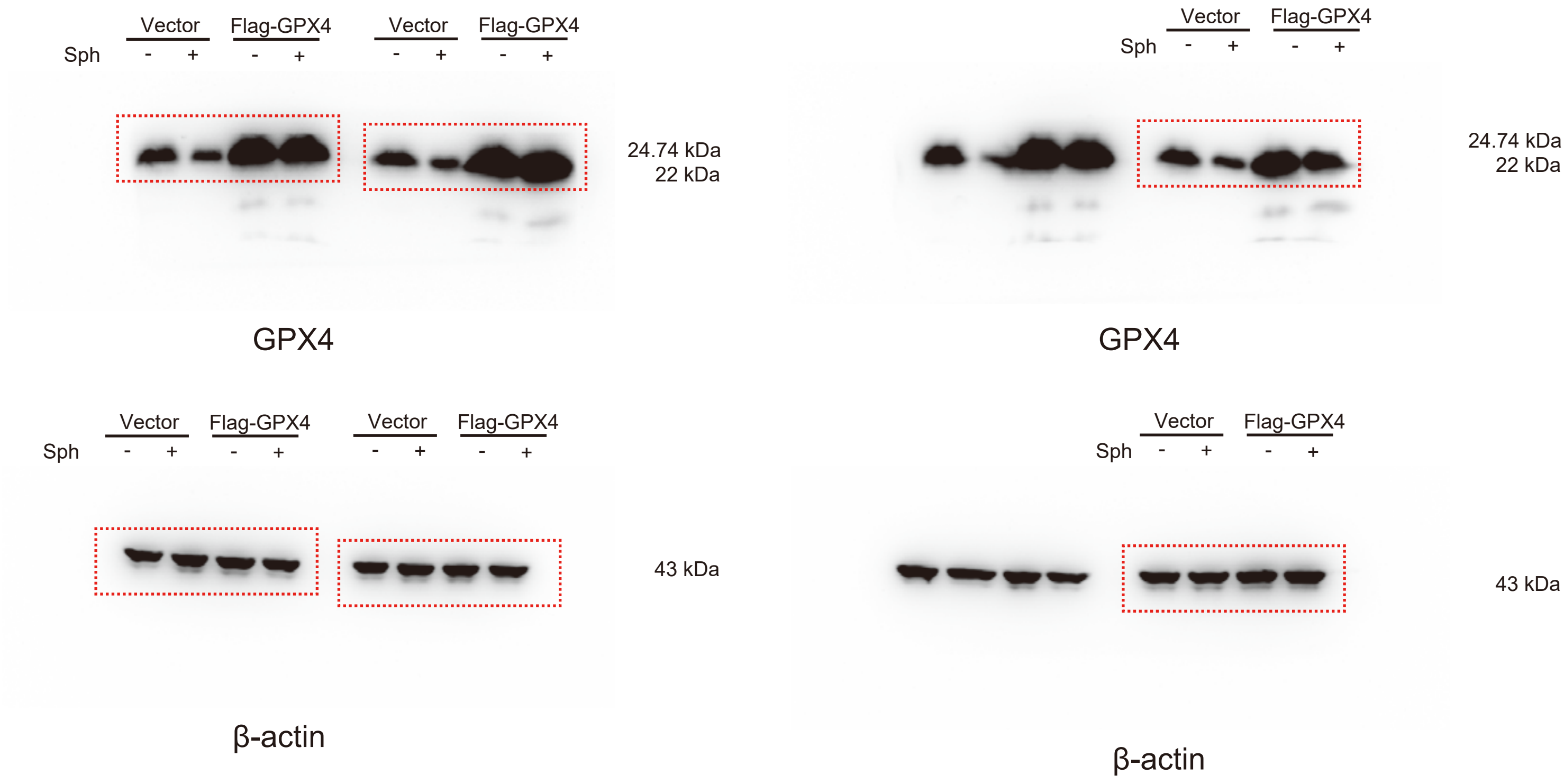

Supplement: Supplementary file 1 [file ppat.1014644.s007.pdf]
