# Supplementary material for: Sph-mediated mtDNA release and autophagy-dependent ferroptosis contribute to Staphylococcus aureus-induced mastitis
Source: PLoS Pathog. 2026 Sep 29;22(9):e1014644. doi: 10.1371/journal.ppat.1014644 (PMC13645233; doi:10.1371/journal.ppat.1014644)
Supplement: Supplementary file 2 [file ppat.1014644.s006.docx]

**Table A. Bacterial strains used in this study**

| **Strain Name** | **Relevant Characteristics/Description** | **Source / Reference** |
| --- | --- | --- |
| *S. aureus* ATCC25923 | Wild-type strain | American Type Culture Collection (ATCC) |
| *S. aureus* ATCC25923 Δ*sph* | Isogenic *sph* deletion mutant derived from S. aureus ATCC 25923 | This study |
| *S. aureus* ATCC25923 Δ*sph*::*sph* | Complemented strain generated by introducing the native *sph* gene into the Δ*sph* mutant | This study |
| *S. aureus* RN4220 | Restriction-deficient derivative of strain 8325-4, used as an intermediate host for plasmid propagation and transformation in *S. aureus* | MIAOLING PLASMID |
| *E. coli* DH5α | Cloning strain used for routine plasmid propagation and amplification | Laboratory Stock |
| *E. coli* BL21(DE3) | Expression strain used for recombinant protein production | Laboratory Stock |

**Table B. Primer Sequence Information**

| **Primer name** | **Restriction endonuclease** | **Primer sequence (5’-3’)** |
| --- | --- | --- |
| ATCC25923 *sph* F | *Bam* HⅠ | CGCGGATCCGCCGAATCTAAGAAAGATGATACTGGTTTG |
| ATCC25923 *sph* R | *Xho* Ⅰ | CCGCTCGAGCTATTTACTATAGGCTTTGATTGGATAATGATC |
| *sph* up F | *Xba* Ⅰ | GTCCATACCCATGGTCTAGAACGTATGGCTATAAAACAGGTGGAGAT |
| *sph* up R |  | ATATAAAAGGAGTGATAATGCACTCAACAAAAAACTCGCTTCGTTCT |
| *sph* down F |  | AGCGAGTTTTTTGTTGAGTGCATTATCACTCCTTTTATATAGCTTAC |
| *sph* down R | *Xho* Ⅰ | GTATATTTTTCTGACTCGAGCTGTCAGATTTCAATACTGATTTAACC |
| *sph* sgRNA F |  | GAAATTCGCAATTGAATTTGATTG |
| *sph* sgRNA R |  | AAACCAATCAAATTCAATTGCGAA |
| pCasSA Kan F |  | GATGGATTGCACGCAGGTTC |
| Overlap Verify F |  | ATAGTCCTGTCGGGTTTCGC |
| Overlap Verify R |  | CATCCGACGCTATTTGTGCC |
| Δ*sph* Verify F |  | AGAAGGATTTAATCCAGAATTTTTAGCTGT |
| Δ*sph* Verify R |  | GAATATCCATGATGTAATGAACTTGATGAT |
| 16S RNA F |  | TATGGAGAGTTTGATCCTGGCTCAGGATGAACGCT |
| 16S RNA R |  | AAGGAGGTGATCCAGCCGCACCTTCCGATACGGCT |
| Δ*sph:sph* PLI 50 F | *Sal* Ⅰ | CCGGTCGACAAATTATCTCTCCTTTTTTATGTAATTCGTGATTG |
| Δ*sph:sph* PLI 50 R | *Kpn* Ⅰ | CGGGGTACCGTTTTCGCAAGTTGCCTGCTGCGTATCACGTAGTA  AAATTATCTCTCCTTTTTTATGTAATTCGTGATTG |
| Mic60_1-590_F | *Bam* HⅠ | CGCGGATCCGCCACCATGCTGCGGGCCTGTCAGTTATCGGGTGTGACC |
| Mic60_1-590_R | *Xba* Ⅰ | TGCTCTAGAGATAGTAGGTGTTTCTGCAGATGAGGTCTTCAT |
| Mic60_35-480_F | *Bam* HⅠ | CGCGGATCCGCCACCATGACTTCAGGCAGCTCTGGGTTGACTACTGGCAAA |
| Mic60_35-480_R | *Xba* Ⅰ | TGCTCTAGATCTCATTTCATTTTCCATGGCATCTCTGACTTC |
| Mic60_35-200_F | *Bam* HⅠ | CGCGGATCCGCCACCATGACTTCAGGCAGCTCTGGGTTGACTACTGGCAAA |
| Mic60_35-200_R | *Xba* Ⅰ | TGCTCTAGATTCAGGTGGTCGCTCCCTTATAGAAGATGAGGA |
| Mic60_201-327_F | *Bam* HⅠ | CGCGGATCCGCCACCATGGAAGTTGCAGCTCGCCTTGCACAACAGGAAAAA |
| Mic60_201-327_R | *Xba* Ⅰ | TGCTCTAGATGCAGCAGTTATATGAGGCTTGGCCCCAGCAAC |
| Mic60_328-590_F | *Bam* HⅠ | CGCGGATCCGCCACCATGGAGGGTAAACTTCACAACATGATAGTTGATCTG |
| Mic60_328-590_R | *Xba* Ⅰ | TGCTCTAGAGATAGTAGGTGTTTCTGCAGATGAGGTCTTCAT |
| B.T *Mfn1* F |  | ACAGCACATGGAAAGATGCC |
| B.T *Mfn1* R |  | CCCTGTGCTTTGTGCTTTCT |
| B.T *Mfn2* F |  | AGAGGGCTCAGAGGAGAAGA |
| B.T *Mfn2* R |  | CTTGAGAAGCGGACACTTGG |
| B.T *Drp1* F |  | CAGAGAGCTCATCCTTCGGT |
| B.T *Drp1* R |  | TGCGACCATCTGGATCTACC |
| B.T *Fis1* F |  | CACAGAACAACCAGGCCAAA |
| B.T *Fis1* R |  | TGCCGTCTCCTTCAGGATTT |
| B.T *β-actin* F |  | TCACCAACTGGGACGACA |
| B.T *β-actin* R |  | GCATACAGGGACAGCACA |
| si-STING1 sense |  | GGAUCCGAAUGUUCAAUCATT |
| si-STING1 antisense |  | UGAUUGAACAUUCGGAUCCTT |
| NC-siRNA sense |  | UUCUCCGAACGUGUCACGUTT |
| NC-siRNA antisense |  | ACGUGACACGUUCGGAGAATT |

**Restriction sites were underlined**
